# Total chemical synthesis of SUMO-2-Lys63-linked diubiquitin hybrid chains assisted by removable solubilizing tags†
†Electronic supplementary information (ESI) available. See DOI: 10.1039/c7sc00488e



**DOI:** 10.1039/c7sc00488e

**Published:** 2017-04-05

**Authors:** Somasekhar Bondalapati, Emad Eid, Sachitanand M. Mali, Cynthia Wolberger, Ashraf Brik

**Affiliations:** a Schulich Faculty of Chemistry , Technion-Israel Institute of Technology , Haifa 3200008 , Israel . Email: abrik@technion.ac.il; b Department of Biophysics and Biophysical Chemistry , Johns Hopkins University School of Medicine , Baltimore , MD 21205 , USA

## Abstract

Small ubiquitin like modifier (SUMO) proteins are known to regulate many important cellular processes such as transcription and apoptosis. Recently, hybrid SUMO-ubiquitin chains containing SUMO-2 linked to Lys63-di-ubiquitin were found to play a major role in DNA repair. Despite some progress in understanding the role of these hybrid chains in DNA repair, there are various fundamental questions remaining to be answered. To further investigate the importance of hybrid SUMO-ubiquitin chains in DNA repair, the homogenous material of these chains, and their unique analogues, are needed in workable quantities. By applying advanced chemical strategies for protein synthesis, we report the first total chemical synthesis of four different SUMO-2-Lys63-linked di-ubiquitin hybrid chains, in which the di-ubiquitin is linked to different lysines in SUMO. In these syntheses, the usefulness of removable solubilizing tags is demonstrated, and two different approaches were examined in terms of reliability and efficiency. In the first approach, a poly-Arg tag was attached to the C-terminus of SUMO *via* a 3,4-diaminobenzoic acid cleavable linker, whereas in the second we attached the tag *via* a phenylacetamidomethyl linker, which can be cleaved by PdCl_2_. The comparison between these different strategies offers guidelines for future scale-up preparation of these analogues and other proteins, which currently use synthetic peptide intermediates that are difficult to handle and purify. The availability of the SUMO-ubiquitin hybrid chains opens up new opportunities for studying the role of these chains in DNA repair and other cellular processes.

## Abstract 2

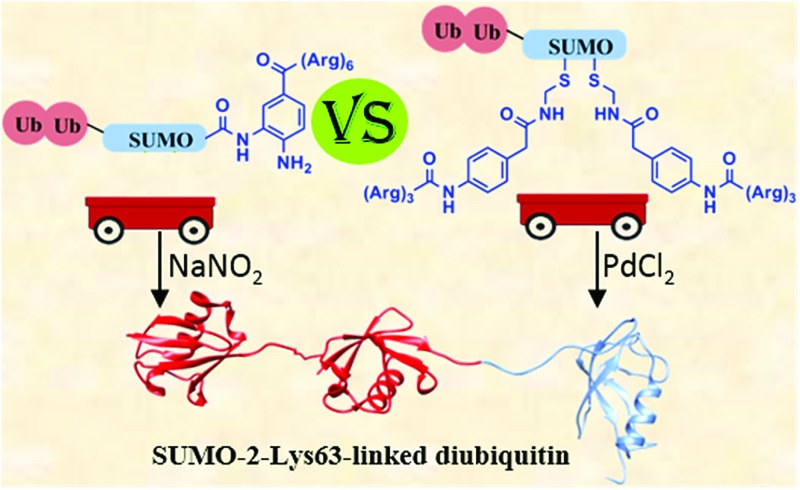
We report the first total chemical synthesis of four different SUMO-2-Lys63-linked di-ubiquitin hybrid chains, in which the di-ubiquitin is linked to different lysines in SUMO.

## Introduction

The covalent attachment of ubiquitin (Ub) and ubiquitin-like proteins to lysines in substrate proteins regulates a vast array of cellular processes.^1,2^ Ubiquitin is an 8.5 kDa, 76-amino acid protein, whereas the small ubiquitin-like modifier (SUMO) proteins have molecular weight ∼10 kDa and exist as four isoforms known as SUMO-1/2/3/4. SUMOylation regulates essential cellular processes such as transcription, mitochondrial dynamics and the DNA damage response.^1,2^ Analogous to ubiquitination, SUMOylation is also mediated by the E1, E2 and E3 enzymes, which catalyze formation of an isopeptide bond between the SUMO C-terminal Gly and the epsilon amino group of a Lys residue in a target protein. Like poly-ubiquitin chains, poly-SUMO-2/3 chains are also found in cells, where one SUMO is connected *via* its C-terminus to a Lys that is present in the consensus motif ΨKXD/E (where Ψ represents a large hydrophobic residue and X any amino acids) of the preceding SUMO.

It was initially believed that SUMO and Ub were independent in their homotypic signaling. However, mass spectrometry studies revealed the presence of ubiquitinated SUMO in cells,^3^ indicating the presence of mixed SUMO-ubiquitin polymers. More recently, hybrid chains consisting of Lys63-linked polyubiquitin conjugated to SUMO-2 chains were shown to play a role in DNA double strand break (DSB) repair by recruiting the BRCA1-A complex^4^ subunit RAP80. This subunit of the BRCA1-A complex was previously known to bind specifically to Lys63-linked di-Ub,^5,6^ and it was shown to also contain a SUMO-interacting motif, suggesting that RAP80 recognizes both ubiquitin and SUMO. Indeed, a hybrid chain containing Lys63-di-Ub linked to SUMO-2 binds 80-fold more tightly to RAP80 than SUMO-2 or Lys63-di-Ub alone.^4^ The enhanced affinity of the hybrid chain to RAP80 is mediated by the tandem SUMO interacting motif and the tandem ubiquitin interacting motifs present in RAP80.^7^

Despite some progress in understanding the involvement of the hybrid chains in DNA DSB repair, there are still many fundamental questions to be answered. For example, there are eight possible lysines in SUMO-2 to which ubiquitin can be conjugated (Lys5, Lys7, Lys11, Lys21, Lys33, Lys35, Lys42 and Lys 45), in addition to the SUMO N-terminus (Met1),^8^ yet it is not known which SUMO-ubiquitin linkage might be optimal for efficient binding to RAP80. Furthermore, the three dimensional structural topologies of the different types of hybrid chains are uncharacterized. Since additional proteins containing SUMO-interacting motifs in proximity to ubiquitin-binding motifs have been identified,^4^ it is possible that proteins other than RAP80 may also recognize hybrid chains in DNA damage or in other cellular events. At least one deubiquitinating enzyme has been identified that specifically removes ubiquitin from SUMO,^9^ although it is not known whether there are additional enzymes specific for the hybrid chains that could also potentially discriminate among the different chain types.

In order to investigate these important questions, homogenous material consisting of hybrid chains in workable quantities is needed. While hybrid chains containing ubiquitin fused to the N-terminus of SUMO can be prepared enzymatically,^4,8^ until this work the other hybrid chains remained inaccessible. Moreover, the probes and unique analogues based on these hybrid chains are often not easy or even possible to prepare by enzymatic approaches. Chemical synthesis should in principle overcome these challenges and make it possible to prepare a desired hybrid chain and its desired analogues. While the synthesis of native SUMO-1/2/3 using ligation approaches^10–12^ and SUMO-linked to Ubc9 *via* a triazole bond has been achieved,^13^ the synthesis of hybrid chains remains inaccessible. We report here for first time the preparation of four different SUMO-2-Lys63-linked-di-Ub analogues by employing current state of the art chemical methods for protein synthesis.^14^ In these syntheses, we encountered major problems in the handling and purification of synthetic peptide intermediates, which gave rise to inefficiencies. To overcome these problems, we examined various synthetic strategies to assist in these syntheses, mainly the use of two methods for attaching solubilizing tags at different positions in SUMO to facilitate synthesis. This approach enabled us to compare the reliability of these methods and select the most efficient one for future synthesis.

## Results and discussion

**Scheme 1:**
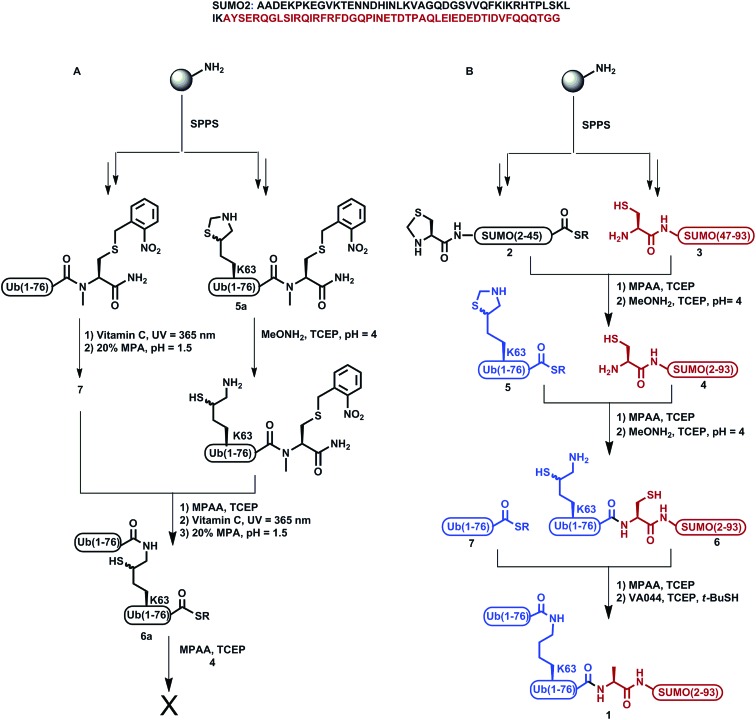
Synthesis of SUMO-2-Ala-diUb(K63), **1** *via* (A) the convergent approach; (B) the sequential ligation strategy.

We previously demonstrated the total chemical- and semi-synthesis of different ubiquitin conjugates with increasing complexity, such as free tetra-Ub chains,^15^ doubly modified H2B histone with glycan and Ub units,^16^ and tetra-ubiquitinated proteins.^17–19^ This has facilitated biochemical, biophysical and functional analyses of these conjugates.^18–20^ Equipped with various synthetic tools for protein synthesis of such complex conjugates, we decided to examine the total chemical synthesis of SUMO-2 linked to Lys63-di-Ub *via* its N-terminus, di-Ub(K63)-SUMO-2 (**1**, Scheme 1), using a convergent approach. Towards this goal, we strategically divided the 93 amino acids of SUMO-2 into two fragments, Thz-SUMO(2-45)-SR, **2**, (Thz: thiazolidine) and Cys-SUMO(47-93)C49S, **3**, (Scheme 1). The N-terminal Met was mutated to Thz to enable ligation with Ub and Ala46 was mutated to Cys to facilitate native chemical ligation (NCL).^21^ As shown in Scheme 1, this Cys residue is converted to alanine during the desulfurization reaction, thus restoring the native sequence. The native Cys49 was mutated to Ser, which is structurally closer to Cys, to prevent its conversion to Ala during desulfurization in the later stage of the synthesis. Fragment **2** was synthesized using Fmoc-SPPS and an *N*-acylurea approach followed by thiolysis after peptide deprotection and cleavage from the resin.^22^ Fragment **3** was also synthesized employing Fmoc-SPPS. This fragment exhibited anomalous behavior in the HPLC purification step, but despite this we were able to successfully obtain the fragment in 8% isolated yield. Having both fragments in hand, we then carried out the ligation reaction, followed by Thz conversion to Cys to obtain Cys-SUMO(2-93), **4**.^23,24^ On the other hand, di-Ub(K63*)-COSR was synthesized employing isopeptide chemical ligation between the two Ub building blocks; namely Ub(K63*)-*N*-methyl-Cys^25^ **5a**, bearing δ-mercaptolysine (K*)^26^ at position 63, and Ub-COSR **7**. This was followed by switching the C-terminal *N*-methyl-Cys to the corresponding thioester of 3-mercapto propionic acid (MPA), **6a**, as we previously reported for the synthesis of Lys48-linked di-Ub.^15^ We then attempted final ligation between Cys-SUMO(2-93) **4** and di-Ub(K63*)-COSR **6a**. However, we did not observe product formation even after 24 h incubation, likely due to the poor solubility of di-Ub(K63*)-COSR in the ligation buffer.

We then moved to examine a sequential ligation strategy. We synthetized Ub(K63*)-COSR analogue, **5**, employing the *N*-methyl-Cys approach^25^ and subjected it to ligation with fragment **4**. Subsequently, δ-mercaptolysine in Ub was deprotected to furnish Ub(K63*)-Cys-SUMO(2-93), **6**, in 28% yield. This was then ligated with Ub-COSR, **7**, to give di-Ub(K63*)-Cys-SUMO(2-93). A final one-pot desulfurization^27^ was then performed to produce the desired diUb(K63)-Ala-SUMO(2-93), **1**, in 23% yield (ESI S23†).

It is important to mention that, in this synthesis, the first ligation between fragments **4** and **5** and the subsequent mercaptolysine deprotection proceeded smoothly, which is clearly evident from analytical HPLC and mass analysis of these reactions. However, the HPLC purification process of the resultant ligation product **6** was very challenging, as we observed dragging of the product—eluting over ∼10 min—which led to an overlap of the impurities and the desired product being obtained in a very low yield (ESI S20†). In addition, we reencountered the same purification problem while isolating the di-Ub(K63)-Ala1-SUMO **1** conjugate. Although this approach was successful in obtaining the final product, the low yield of the final product (<2%) and the difficulties in the purification steps make it very difficult to apply in the synthesis of the analogue and other analogues based on the hybrid chains.

We speculated that the peptide behaviors in HPLC analysis and purification might be due to the hydrophobic nature of the synthetic intermediates and decided to install a removable solubilizing tag in fragment **3** during SPPS. This could in principle increase the solubility, lower the dragging of the peptide fragments, and ease the handling and purification of the intermediates and final conjugate. A variety of methods have been developed to aid the synthesis of hydrophobic proteins or those whose synthetic intermediates are difficult to purify and use in the ligation.^28–33^ In considering this problem, we were inspired by the recent work of Liu and coworkers, in which the 3,4-diaminobenzoic acid (Dbz) linker^22^ was employed to attach a poly-Arg tag to the C-terminus of a hydrophobic peptide fragment to increase its solubility in the ligation buffer, and assist in its purification.^34^ Subsequently, the Dbz linked to the (Arg)_6_ tag was switched to the thioester by treatment with sodium nitrite followed by thiolysis with exogenous thiol to enable the next ligation. We also chose to utilize the Dbz linker to install an (Arg)_6_ tag at the C-terminus of Cys-SUMO(47-93) hoping to control the anomalous behavior of this fragment during the preparative HPLC purification and improve the overall yield. We also hoped this would ease the handling of the SUMO-Ub conjugates in the purification steps. In our case, the tag could be removed by converting the Dbz into the more reactive triazole intermediate followed by hydrolysis.

**Scheme 2:**
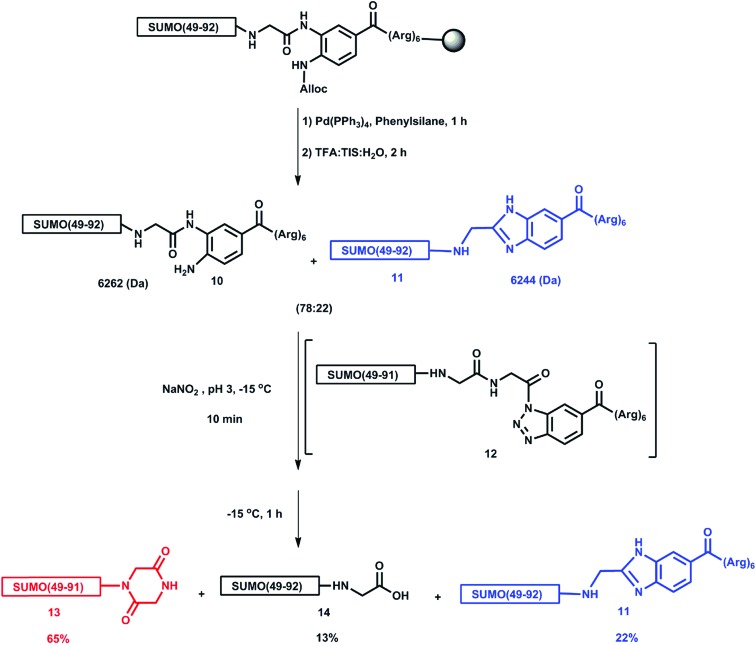
Hydrolysis products of the mixture containing **10** and **11**.

We first attempted the synthesis of a model peptide bearing a Dbz linked (Arg)_6_ tag at the SUMO C-terminus, SUMO(49-93)-Dbz-(Arg)_6_ **10**, using allyloxycarbonyl (Alloc) protection on the Dbz during SPPS. After peptide elongation, the alloc protection was removed and the peptide was cleaved from the resin to obtain **10**. The mass analysis of the reaction mixture showed two masses of 6262.2 Da and 6244.1 Da in a ratio of 78 : 22 (ESI S25†). We presumed that under strong acidic conditions, the free amine of the Dbz might have condensed with the carbonyl of the less sterically hindered C-terminal Gly to give a SUMO-(49-92)-NHCH_2_-benzimidazole side product **11** with a mass of 6244.1 Da (Scheme 2). This assumption was further supported by analyzing the resulting mixture of the removal step of the Dbz-(Arg)_6_ tag. Upon treatment with 60 equiv. of NaNO_2_ at –15 °C for 10 minutes we observed the formation of a benzotriazole intermediate **12**. Extending the reaction for an extra hour at the same temperature provided a mixture containing SUMO(49-91)-piperazine-2,5-dione **13**, the desired hydrolyzed product SUMO(49-93)-COOH **14** and the unreacted **11** (ESI S27†). We reasoned that, since the peptide benzotriazole **12** is highly reactive, it spontaneously undergoes hydrolysis and intramolecular nucleophilic attack by the amide of the subsequent Gly on the activated C-terminus, also leading to the formation of piperazine-2,5-dione **13**.

To control the formation of the benzimidazole side product **11**, we synthesized SUMO(49-93)-Dbz-(Arg)_6_ while protecting the free amine with a propargyloxycarbonyl (Proc) group. The peptide was cleaved from the resin with subsequent removal of the Proc group using PdCl_2_^24^ on the crude peptide in Gn·HCl to obtain SUMO(49-93)-Dbz-(Arg)_6_ **10** without the formation of any side products. The Proc group was chosen instead of Alloc for protection because its removal in aqueous solution using palladium is much more efficient. In addition, to avoid piperazine-2,5-dione **13** formation, we chose to use mercaptoethanol in this reaction that could rapidly form a thioester by intercepting the benzotriazole intermediate **12**. To our delight, adding an excess of mercaptoethanol to the benzotriazole **12**, and continuing the reaction for an additional hour, led to the quantitative formation of the thioester, SUMO(49-93)-COSCH_2_CH_2_OH. Subsequent elevation of the reaction pH to 9,^35^ while keeping it for an additional hour at room temperature, provided the desired SUMO(49-93)-COOH, (ESI, S30†).

**Scheme 3:**
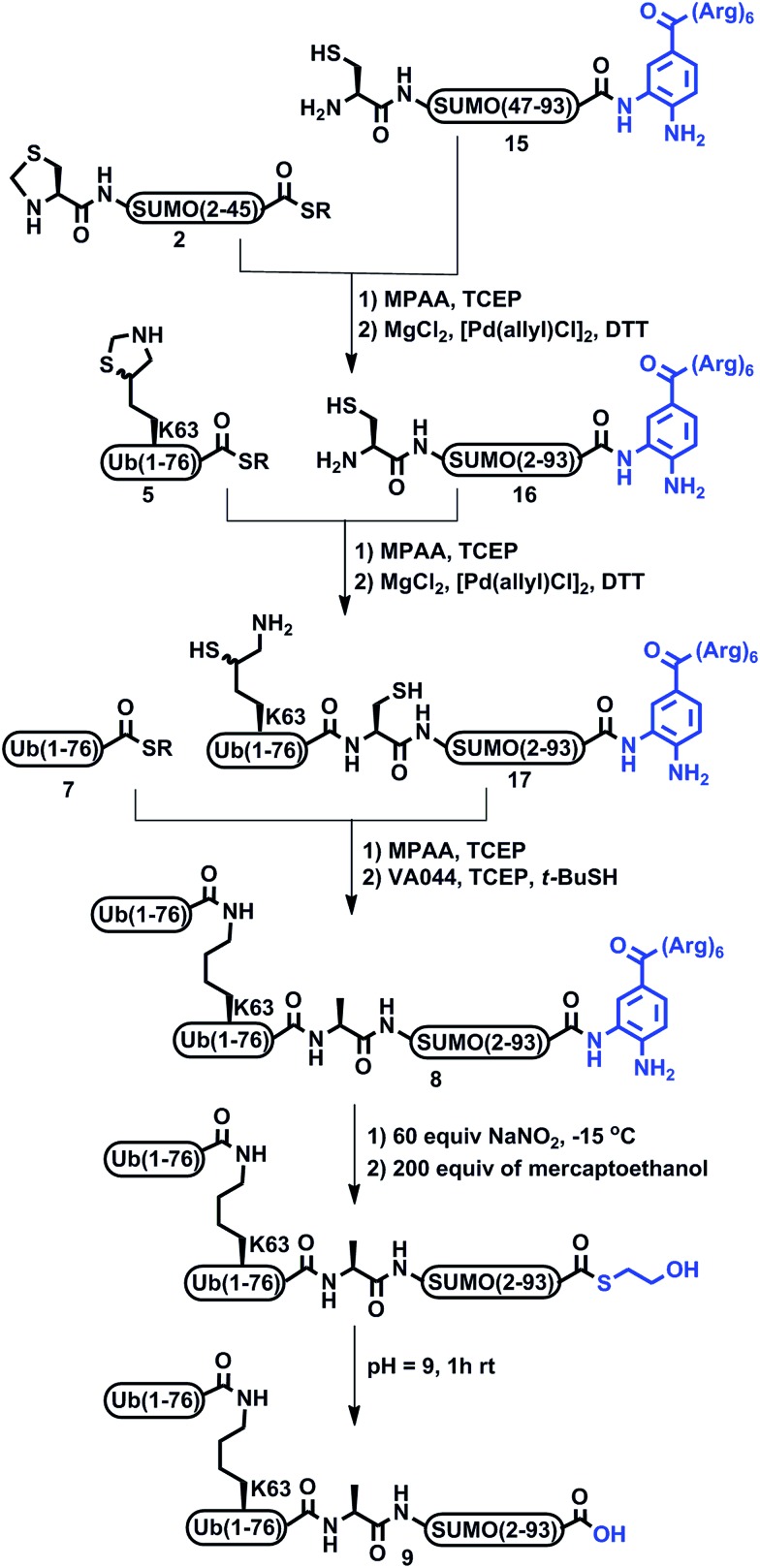
Synthesis of di-Ub(K63)-Ala-SUMO using Dbz anchored poly-Arg tag and its removal.

**Fig. 1:**
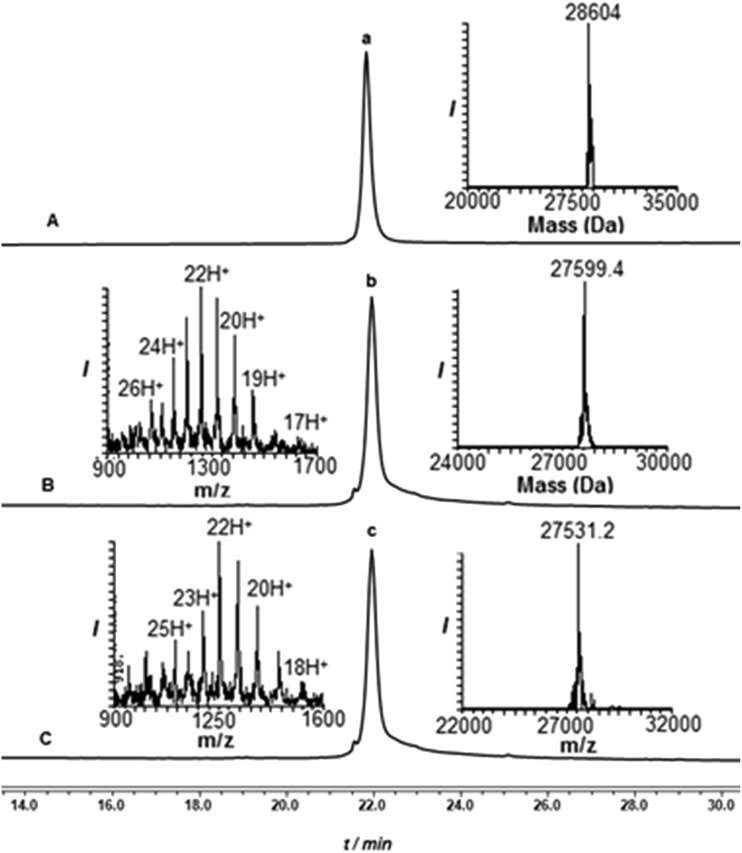
HPLC and mass analysis of removal of the Dbz-(Arg)_6_ tag from **8** (peak a). (A) At time zero before treatment with 60 eq. NaNO_2_. (B) 1 h after the addition of 200 eq. of MPA following treatment with NaNO_2_. (C) After 1 h at pH 9 and room temperature (peak c corresponds to desired product **9**).

Encouraged by these results with the model peptide, we then implemented this approach for the synthesis of di-Ub(K63)-Ala-SUMO(2-93). We first synthesized Cys-SUMO(47-93)-Dbz-(Arg)_6_ **15** and ligated it with fragment **2**, which was followed by Thz removal to obtain Cys-SUMO(2-93)-Dbz-(Arg)_6_ **16** (Scheme 3). At this stage of this work, and in a parallel project, we discovered the efficient removal of Thz by palladium complexes,^24^ hence we began to employ Pd[(allyl)Cl]_2_ for Thz removal instead of the traditional conditions of using methoxylamine in pH 4. Polypeptide **16** was then subjected to ligation with Ub(K63*)-COSR **5**, bearing protected δ-mercaptolysine at position 63, followed by Thz-removal to furnish Ub-(K63*)-Cys-SUMO(2-93)-Dbz-(Arg)_6_ **17**. It should be mentioned that, although HPLC analysis with and without the tag gave a similar pattern, **17** exhibited much less dragging in preparative HPLC in comparison with the conjugate without a tag, and the product was isolated in 35% yield. The next ligation with **7** also proceeded smoothly, and subsequent desulfurization afforded **8**, which upon the removal of the solubilizing tag, and when employing the above established conditions, afforded the desired product **9** in 2–3% overall yield (Fig. 1) starting from fragment **15**.

Although the incorporation of a Dbz based solubilizing tag assisted in isolating the SUMO and SUMO-Ub conjugates, the key fragment Cys-SUMO(47-93)-Dbz-(Arg)_6_ **15** was obtained in very low yield (∼6%). In addition, the requirement for multiple steps to remove the tag rendered this approach low yielding and operationally difficult, in particular when dealing with small-scale synthesis. This prompted us to search for other alternative approaches for enhancing the solubility of the peptide fragments. During the course of validating the applicability of a Dbz based solubilizing tag, our laboratory has invented a phenylacetamidomethyl (Phacm) based removable solubilizing tag applicable to protein synthesis.^28^ This newly developed linker is stable under SPPS and NCL conditions and can be cleaved quantitatively using PdCl_2_ after completing the synthesis of the target protein. Therefore, we decided to examine the effect of the Phacm based solubilizing tag in the synthesis of di-Ub(K63)-Ala-SUMO(2-93) **1**. One obvious advantage of this approach is the opportunity to distribute the solubilizing tag along the polypeptide sequence, in contrast with the previous method, which is mainly limited to the C-terminus. We therefore wondered if installing the two Phacm linked solubilizing tags across the SUMO chain would improve the behavior of the SUMO-Ub conjugates during purification and the overall yield.

**Scheme 4:**
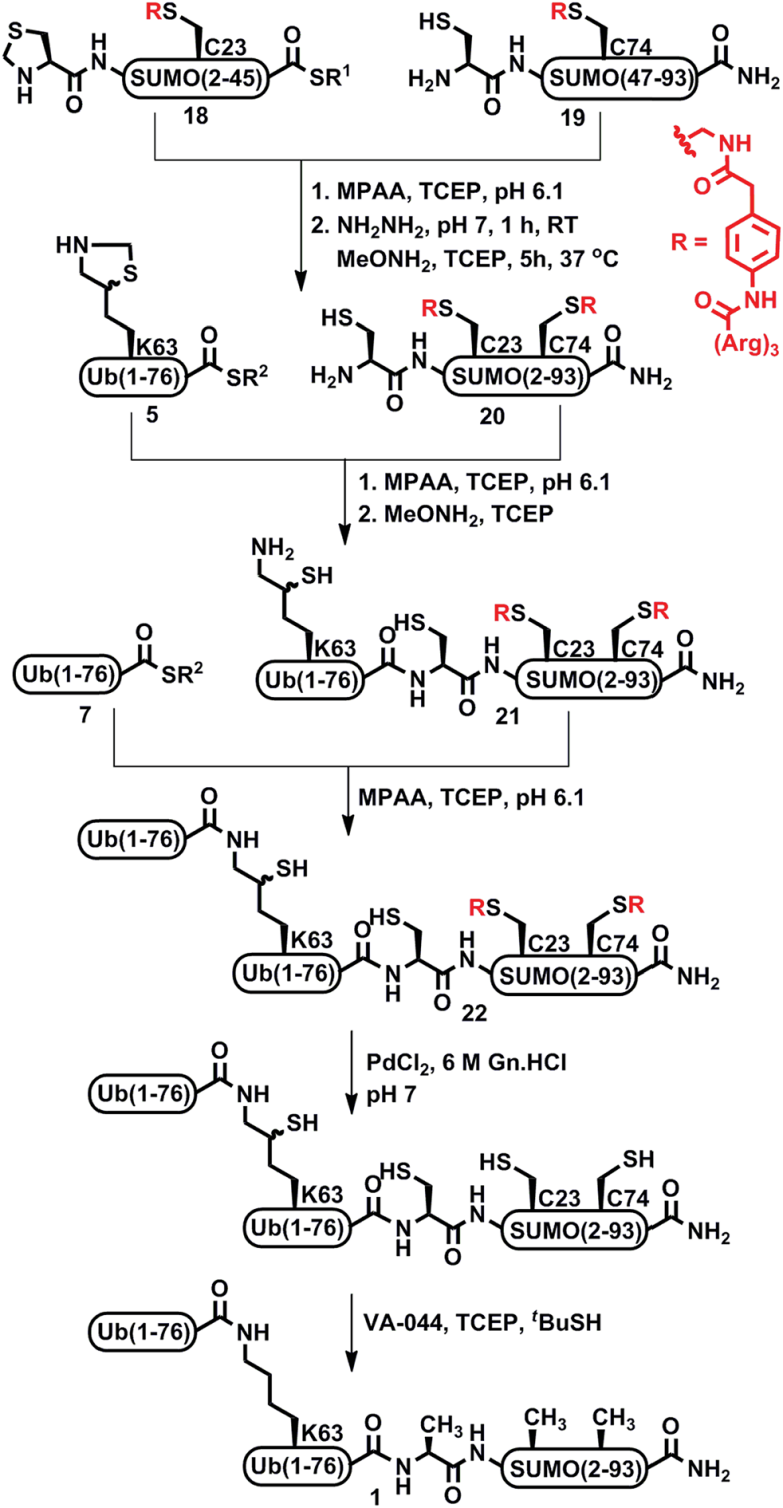
Synthesis of di-Ub(K63)-A1-SUMO-(2-93) assisted by Phacm linked (Arg)_3_ tags.

To examine this approach, we synthesized two SUMO fragments, Thz-SUMO-(2-45)-COSR and Cys-SUMO-(47-93), harboring a solubilizing tag, where Ala 23 and 74 will be converted temporarily to Alloc protected Phacm [abbreviated as Thz-SUMO-(2-45 A23C*)-COSR, **18** and Cys-SUMO-(47-93 A74C*), **19**], as shown in Scheme 4. Both fragments were obtained in good yields and purity when employing SPPS (ESI†). Notably, while in the previous approach fragment **15** was difficult to obtain in good yield, the equivalent fragment **19** was efficiently prepared and isolated in ∼10% yield compared to 6% for fragment **15**. Having the two fragments in hand, we then proceeded with NCL, followed by Thz opening with MeONH_2_ to obtain Cys-SUMO-(2-93, A23C*, A74C*) **20**. Subsequent ligation with **5** and Thz-opening provided Ub(K63*)-Cys-SUMO-(2-93, A23C*, A74C*) **21**. Gratifyingly, with this strategy we achieved complete control of the dragging of the peptide during purification and obtained the resultant conjugate in very good yield and high purity. This is also clearly evident from the comparison of the preparative HPLC patterns of the Ub(K63*)-SUMO conjugate with and without tags (ESI, S53†). Further, di-Ub-(K63*)-Cys-SUMO(2-93, A23C*, A74C*) **22** was obtained from the ligation between **21** and **7**.

**Fig. 2:**
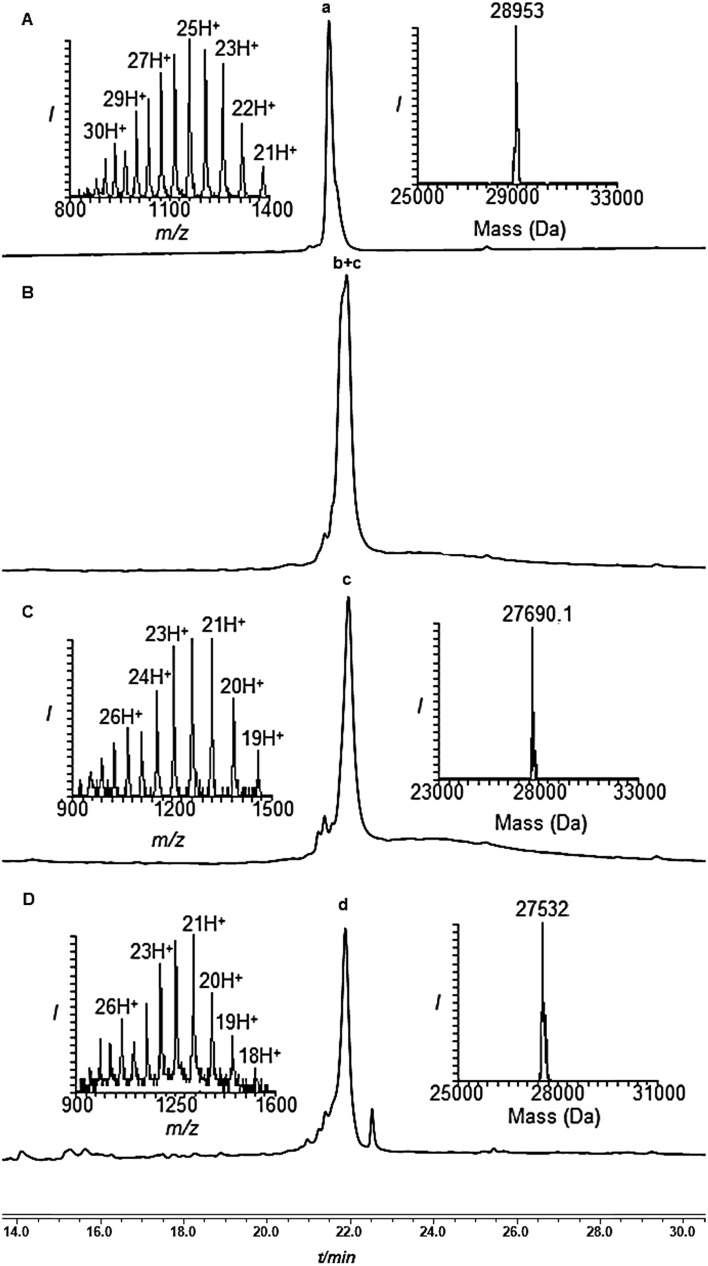
Removal of the solubilizing tag from di-Ub(K63)-Cys-SUMO-(2-93 A23C*, A74C*) using PdCl_2_. (A) Analytical HPLC and mass trace analysis of di-Ub(K63)-Cys-SUMO-(2-93 A23C*, A74C*) (peak a, observed mass 28 953 ± 3.2 Da, calcd 28 957.0 Da) and PdCl_2_ at time zero. (B) Removal after 1 h: peak b + c corresponds to removal of one and two tags with observed masses of 28 322.0 Da and 27 690.1 Da, respectively. (C) Removal after 2 h: peak c corresponds to the complete removal of the two tags with the observed mass of 27 690.1 ± 3.5 Da, calcd 27 695.5. (D) After 8 h of desulfurization of the dialyzed reaction mixture: peak d corresponds to the complete removal of the two tags with the observed mass of 27 532.0 ± 1.1 Da, calcd 27 535.2 Da.

Subsequently, the two Phacm linked solubilizing tags were removed quantitatively by treating with PdCl_2_ in Gn·HCl buffer at pH 7 for 2 h (Fig. 2). The reaction was quenched with an excess of DTT, dialyzed against Gn·HCl at pH 7, and subjected to desulfurization to produce the desired product **1** in 39% yield and 6% overall yield starting from fragment **19** (ESI, S51†), which is two-fold better compared to synthesis based on the Dbz tag. We successfully applied a similar strategy to synthesize di-Ub(K63)-Lys11-SUMO-2, di-Ub(K63)-Lys33-SUMO-2 and di-Ub(K63)-Lys42-SUMO-2. These analogues were then folded in Tris buffer solution of pH ∼ 7.3 and their secondary structures were examined by circular dichroism, which suggested correct folding of these chains (ESI, S54†). We are currently building on our optimized synthetic approach to prepare workable quantities of these chains and use them in a variety of biochemical, proteomic and structural studies, which will be reported in due course.

In summary, we have accomplished total chemical synthesis of four different di-Ub(K63)-SUMO-2 analogues. During our attempts at synthesis, major handling and purification problems were encountered, which forced us to attach poly-Arg tag(s) to SUMO to assist in overcoming these obstacles. Two strategies were examined, in the first approach a poly-Arg tag was attached to the C-terminus of SUMO *via* a Dbz-cleavable linker. In the second approach, we attached the poly-Arg tags *via* the newly developed Phacm linker, which is cleaved by PdCl_2_ upon completion of the synthesis. Although both were successful in overcoming the above-described challenges, the approach based on the Phacm linker turned out to be more efficient and reliable, and it required fewer steps. The current study and the developed synthetic approaches open new horizons for studying the role of the hybrid chains in DNA repair and should enable studies that otherwise are difficult or impossible to conduct. Moreover, the synthetic lessons learned here should assist in the synthesis of other challenging proteins.

## References

[cit1] Sriramachandran A. M., Dohmen R. J. (2014). Biochim. Biophys. Acta.

[cit2] Wilkinson K. A., Henley J. M. (2010). Biochem. J..

[cit3] Tatham M. H., Geoffroy M. C., Shen L., Plechanovova A., Hattersley N., Jaffray E. G., Palvimo J. J., Hay R. T. (2008). Nat. Cell Biol..

[cit4] Guzzo C. M., Berndsen C. E., Zhu J., Gupta V., Datta A., Greenberg R. A., Wolberger C., Matunis M. J. (2012). Sci. Signaling.

[cit5] Sobhian B., Shao G., Lilli D. R., Culhane A. C., Moreau L. A., Xia B., Livingston D. M., Greenberg R. A. (2007). Science.

[cit6] Kim H., Chen J., Yu X. (2007). Science.

[cit7] Sims J. J., Cohen R. E. (2009). Mol. Cell.

[cit8] Tatham M. H., Plechanovova A., Jaffray E. G., Salmen H., Hay R. T. (2013). Biochem. J..

[cit9] Lecona E., Rodriguez-Acebes S., Specks J., Lopez-Contreras A. J., Ruppen I., Murga M., Munoz J., Mendez J., Fernandez-Capetillo O. (2016). Nat. Struct. Mol. Biol..

[cit10] Drobecq H., Boll E., Senechal M., Desmet R., Saliou J. M., Lacapere J. J., Mougel A., Vicogne J., Melnyk O. (2016). Bioconjugate Chem..

[cit11] Melnyk O., Vicogne J. (2016). Tetrahedron Lett..

[cit12] Wucherpfennig T. G., Pattabiraman V. R., Limberg F. R., Ruiz-Rodriguez J., Bode J. W. (2014). Angew. Chem., Int. Ed..

[cit13] Sommer S., Weikart N. D., Brockmeyer A., Janning P., Mootz H. D. (2011). Angew. Chem., Int. Ed..

[cit14] Bondalapati S., Jbara M., Brik A. (2016). Nat. Chem..

[cit15] Kumar K. S., Bavikar S. N., Spasser L., Moyal T., Ohayon S., Brik A. (2011). Angew. Chem., Int. Ed..

[cit16] Seenaiah M., Jbara M., Mali S. M., Brik A. (2015). Angew. Chem., Int. Ed..

[cit17] Haj-Yahya M., Fauvet B., Herman-Bachinsky Y., Hejjaoui M., Bavikar S. N., Karthikeyan S. V., Ciechanover A., Lashuel H. A., Brik A. (2013). Proc. Natl. Acad. Sci. U. S. A..

[cit18] Hemantha H. P., Bavikar S. N., Herman-Bachinsky Y., Haj-Yahya N., Bondalapati S., Ciechanover A., Brik A. (2014). J. Am. Chem. Soc..

[cit19] Singh S. K., Sahu I., Mali S. M., Hemantha H. P., Kleifeld O., Glickman M. H., Brik A. (2016). J. Am. Chem. Soc..

[cit20] Morgan M. T., Haj-Yahya M., Ringel A. E., Bandi P., Brik A., Wolberger C. (2016). Science.

[cit21] Dawson P. E., Muir T. W., Clark-Lewis I., Kent S. B. (1994). Science.

[cit22] Blanco-Canosa J. B., Dawson P. E. (2008). Angew. Chem., Int. Ed..

[cit23] Bang D., Kent S. B. (2004). Angew. Chem., Int. Ed..

[cit24] Jbara M., Maity S. K., Seenaiah M., Brik A. (2016). J. Am. Chem. Soc..

[cit25] Erlich L. A., Kumar K. S., Haj-Yahya M., Dawson P. E., Brik A. (2010). Org. Biomol. Chem..

[cit26] Kumar K. S., Spasser L., Erlich L. A., Bavikar S. N., Brik A. (2010). Angew. Chem., Int. Ed..

[cit27] Moyal T., Hemantha H. P., Siman P., Refua M., Brik A. (2013). Chem. Sci..

[cit28] Maity S. K., Mann G., Jbara M., Laps S., Kamnesky G., Brik A. (2016). Org. Lett..

[cit29] Jacobsen M. T., Petersen M. E., Ye X., Galibert M., Lorimer G. H., Aucagne V., Kay M. S. (2016). J. Am. Chem. Soc..

[cit30] Olschewski D., Becker C. F. (2008). Mol. BioSyst..

[cit31] Wucherpfennig T. G., Rohrbacher F., Pattabiraman V. R., Bode J. W. (2014). Angew. Chem., Int. Ed..

[cit32] Zheng J. S., He Y., Zuo C., Cai X. Y., Tang S., Wang Z. A., Zhang L. H., Tian C. L., Liu L. (2016). J. Am. Chem. Soc.

[cit33] Johnson E. C., Kent S. B. (2007). Tetrahedron Lett..

[cit34] Wang J. X., Fang G. M., He Y., Qu D. L., Yu M., Hong Z. Y., Liu L. (2015). Angew. Chem., Int. Ed..

[cit35] Gates Z. P., Stephan J. R., Lee D. J., Kent S. B. H. (2013). Chem. Commun..

