# Supplementary material for: Total chemical synthesis of SUMO-2-Lys63-linked diubiquitin hybrid chains assisted by removable solubilizing tags
Source: Chem Sci. 2017 Apr 5;8(5):4027–34. doi: 10.1039/c7sc00488e (PMC5434752; doi:10.1039/c7sc00488e)
Supplement: Supplementary file 1 [file SC-008-C7SC00488E-s001.pdf]

Electronic Supplementary Material (ESI) for Chemical Science.
This journal is © The Royal Society of Chemistry 2017

Supporting Information

Total Chemical Synthesis of SUMO-2-Lys63-linked diubiquitin Hybrid Chains Assisted by Removable Solubilizing Tags

Somasekhar Bondalapati†[1], Emad Eid†[1], Sachitanand M. Mali [1], Cynthia Wolberger[2], Ashraf Brik*[1].

[1] Schulich Faculty of Chemistry, Technion-Israel Institute of Technology, Haifa 3200008, Israel.

[2] Department of Biophysics and Biophysical Chemistry, Johns Hopkins University School of Medicine, Baltimore, MD 21205, USA.

† These authors contributed equally.

Corresponding author: abrik@technion.ac.il

General methods……………………………………………………………….……………….…S4

Synthesis of Thz-SUMO(2-45)-MMP 2………………………….……….....….…S5

Synthesis of Cys-SUMO(47-93) 3 ……………………………….…………..…S7

Synthesis of Cys-SUMO(2-93) 4 …………………………………..………..…...S9

Synthesis of Ub(K63*)-N-methyl-Cys ………………………………………....S12

Synthesis of Ub-MPA (MPA: Mercapto propionic acid) 7 ……………………S14

Synthesis of di-Ub(K63*)-MPA ……………………………………………………S15

Ligation reaction between Cys-SUMO(2-93) 4 and di-Ub(K63*)-MPA ……...S18

Synthesis of Ub(K63*)-MPA 5 ………………………………………………..S19

Synthesis of Ub(K63*)-Cys-SUMO(2-93) 6 ……………………...……..…S20

Synthesis of diUb(K63)-Ala-SUMO(2-93) 1..……………………………………S23

Synthesis of model peptide SUMO(49-93)-Dbz-(Arg)$_6$ 10 ……………………..S25

Cleavage of Dbz-(Arg)$_6$ from SUMO(49-93)-Dbz-(Arg)$_6$ …………………....S27

Synthesis of SUMO(49-93)-Dbz-6(Arg) 10 using proc protection …………….S29

Cleavage of Dbz-(Arg)$_6$ linker on model peptide SUMO(49-93) ……………...S30

Synthesis of Cys-SUMO(47-93)-Dbz-(Arg)$_6$ 15 ………………………….….…….S32

Synthesis of Cys-SUMO(2-93)-Dbz-Arg$_6$ ………….……………………………S33

Synthesis of Ub(K63*)-Cys-SUMO(2-93) -Dbz-Arg$_6$ …….………….…………S35

Synthesis of di-Ub(K63*)-Ala-SUMO(2-93)-Dbz-Arg$_6$ 8 ……………………....S37

Cleavage of Dbz-(Arg)$_6$ tag from di-Ub(K63)-Ala-SUMO(2-93)-Dbz-Arg$_6$ 8....S39

Synthesis of Cys-SUMO-(47-93, A74C*) ……………………………………..S42

Synthesis of Thz-SUMO-(2-45, A23C*)-MMP 18 ……………………...……S43

Synthesis of Cys-SUMO(2-93) bearing two Phacm tags 20 ………………....S45

Synthesis of Ub(K63*)-Cys-SUMO(47-93) bearing two Phacm tags 21 ...……S47

Synthesis of di-Ub(K63)-Cys-SUMO(2-93) bearing two Phacm tags 22 …..….S49

One pot Phacm removal and desulfurization of di-Ub(K63)-Ala-SUMO-(2-93)...

…………………………………………………………………………………...S51

HPLC overlay of Ub(K63)-SUMO-2 purification.……………………….…………..S53

CD of SUMO, SUMO-Ub, SUMO-diUb and K63-diUb....…………….…..……S54

CD and mass analysis of di-Ub(K63)-Lys11-SUMO-2....…..…………..………S54

CD and mass analysis of di-Ub(K63)-Lys33-SUMO-2....…..…………..………S55

CD and mass analysis of di-Ub(K63)-Lys42-SUMO-2....…..…………..………S56

Experimental Section

Materials and methods

1. General methods:

SPPS was carried out manually in syringes, equipped with Teflon filters, purchased from Torviq or by using an automated peptide synthesizer (CS336X, CSBIO). Analytical grade N,N-Dimethylformamide (DMF) was purchased from Biolab. Commercial reagents were used without further purification. Resins were purchased from Creosalus and all protected amino acids were purchased from GL Biochem. The activating reagents [(2-(1H-benzotriazol-1-yl)-1,1,3,3-tetramethyluronium hexafluorophosphate (HBTU), hydroxybenzotriazole (HOBt),[(6-chlorobenzotriazol-1-yl)oxy-(dimethylamino)methylidene]- dimethyl-azanium hexafluorophosphate (HCTU), 1-[Bis(dimethylamino)methylene]-1H-1,2,3-triazolo[4,5-b]pyridinium 3-oxid hexafluorophosphate (HATU)] were purchased from Luxembourg Bio Technologies. Unless otherwise mentioned, all reactions were carried out at room temperature.

List of the protected amino acids used in peptides synthesis: Fmoc-Gly-OH, Fmoc-Ala-OH, Fmoc-Val-OH, Fmoc-Leu-OH, Fmoc-Ile-OH, Fmoc-PheOH, Fmoc-Pro-OH, Fmoc-His(Trt)-OH, Fmoc-Asn(Trt)-OH, Fmoc-Gln(Trt)-OH, Fmoc-Arg(Pbf)-OH, Fmoc-Lys(Boc)-OH, Fmoc-Tyr(^{t}Bu)-OH, Fmoc-Ser(^{t}Bu)-OH, Fmoc-Thr(^{t}Bu)-OH,, Fmoc-Asp(O^{t}Bu)-OH, Fmoc-Glu(O^{t}Bu)-OH, Fmoc-Trp(Boc)

-OH, Boc-Cys(Trt)-OH, Boc-Thz-OH, Boc-Nle-OH Fmoc-Nle-OH, Fmoc-Leu-Ser(ψMe,MePro)-OH, Fmoc-Asp(OtBu)-(Dmb)Gly-OH, Fmoc-Ile-Thr(ψMe,MePro)-OH, Fmoc-Leu-Thr(ψMe,MePro)-OH,

3-Amino-4-methylamino-benzoic acid (Fmoc-MeDbz),[1] Fmoc-N-methyl-Cys,[2] Fmoc-Cys(4-Allyloxycarbonylamino-Phacm)[3] was prepared using the reported procedure.

Reverse phased high-pressure liquid chromatography (RP-HPLC)

Analytical HPLC was performed on a Thermo instrument (Dionex Ultimate 3000) using analytical columns Xbridge (waters, BEH300 C4, 3.5µm, 4.6 × 150 mm) and XSelect (waters, CSH C18, 3.5 µm, 4.6 × 150 mm) at flow rate of 1.2 mL/min. Preparative HPLC

was performed on a Waters instrument using Jupiter 5 μm, C18/C4 300 Å, 250 × 22.4 mm and XSelect (waters, C18, 10 μm, 19 × 250 mm) at flow rate of 15 mL/min. Semi preparative HPLC was performed on a Thermo Scientific instrument (Spectra System SCM1000) using Jupiter C4 10 μm, 300 Å, 250 × 10 mm column, at flow rate of 4 mL/min. All synthetic products were purified by HPLC and characterized by mass spectrometry using LCQ Fleet Ion Trap (Thermo Scientific). All calculated masses have been reported as an average isotope composition.

Buffer A: 0.1% TFA in water; buffer B: 0.1% TFA in acetonitrile.

Circular Dichroism Analysis: CD spectra were recorded using a Chriascan qCD spectropolarimeter. CD spectra were measured at 10 μM protein concentration in a TRIS buffer solution having 50 mM TRIS base and 150 mM NaCl at pH~7.3.

Synthesis of Thz-SUMO(2-45)-MMP 2:

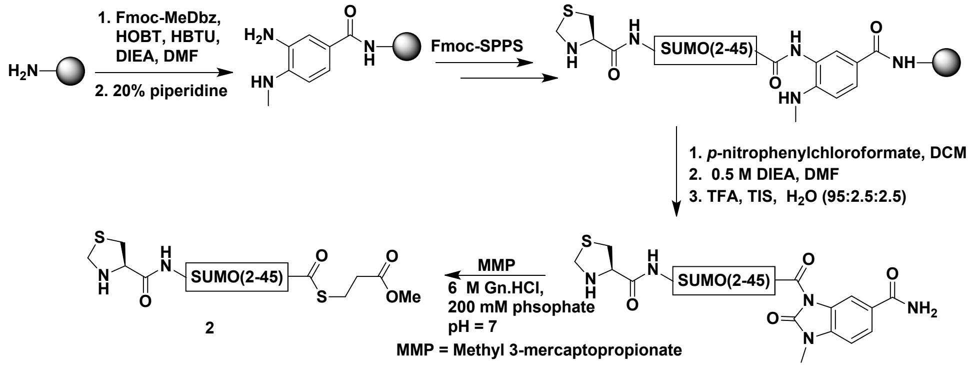

The Fmoc-MeDbz was coupled with 4 equiv of HBTU, 4 equiv of HOBt and 8 equiv of DIEA on Fmoc deprotected Rink amide resin for 2 h. This was followed by Fmoc deprotection and manual coupling of Lys45 with 4 equiv of AA, 4 equiv of HCTU and 8 equiv of DIEA for 45 min (2 cycles). The subsequent Fmoc-removal and the coupling of remaining amino acids were achieved using automated peptide synthesizer in presence of 4 equiv of AA, 8 equiv of DIEA and 4 equiv of HCTU of the initial loading of the resin. Dmb protected dipeptide Asp-Gly(Dmb) was manually coupled at Asp26-Gly27 with 2.5

equiv of Fmoc-Asp(O^{t}Bu)-(Dmb)Gly-OH, 2.5 equiv of HATU and 5 equiv of DIEA. The N-terminal Met was mutated to Thz.

MeDbz cyclization: The resin was washed with DCM and a solution of *p*-nitrophenyl chloroformate (100 mg, 5 equiv) in 4 mL of dry DCM was added, shaken for 30 min at 25 ^oC and washed with DCM (3 × 5 mL). This step was repeated two more times. Following this, the resin was washed with DCM (3 × 5 mL) and DMF (3 × 5 mL), and a solution of 0.5 M DIEA in DMF (5 mL) was added and shaken for 10 min. this step was repeated for another two times to ensure complete cyclization. Finally, the resin was washed with DMF (3 × 5 mL).

Cleavage of the peptide from resin: Cleavage of the peptide from resin: A mixture of trifluoroacetic acid (TFA): triisopropylsilane (TIS): water (H_2O) (95:2.5:2.5) was added to the dried peptide-resin and the reaction mixture was shaken for 2 h at RT. The resin was removed by filtration and was washed with additional TFA (2 × 2 mL). To precipitate the peptide, the combined filtrate was added drop-wise to 10-fold volume of cold ether followed by centrifugation, decanting of ether and by dissolution of residue in acetonitrile-water for freeze drying in the lyophilizer.

Switching of Nbz to MMP thioester: The crude SUMO(1-45)-MeNbz (50 mg, 9.5×10^{-3} mmol) was dissolved in 6 M Gn•HCl buffer(1.5 mL, 6 mM). This mixture was treated with MMP (120 μL, 1.2 mM)) for 1 h at 37 ^oC (pH 7). The product was purified by preparative HPLC, using a gradient of 0-60%B over 40 min to afford Thz-SUMO(2-45)-MMP in 16% yield (8 mg).

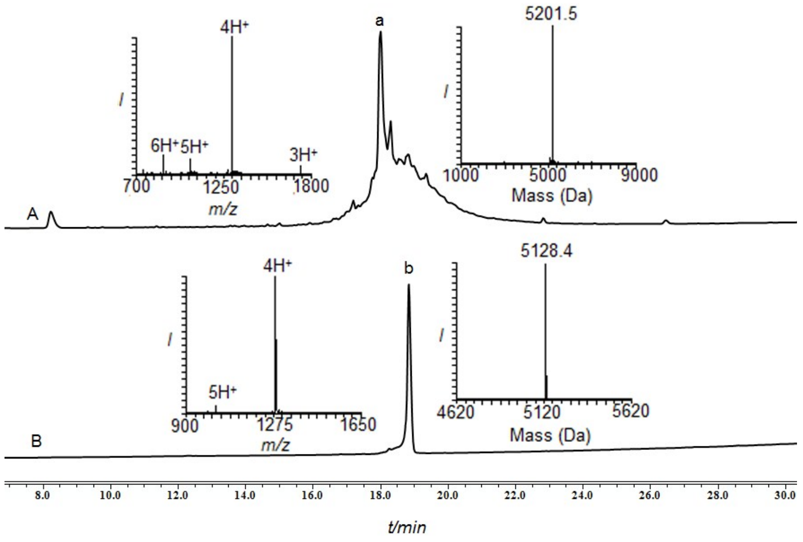

Figure S1: Synthesis of Thz-SUMO(2-45)-MMP: A) Analytical HPLC of crude Thz-SUMO(2-45)-Nbz (peak a with observed mass 5201.5 ± 0.6 Da, (calcd 5199.9 Da). B) Analytical HPLC and mass analysis of purified Thz-SUMO(2-45)-MMP: peak b with observed mass 5128.4 ± 0.2 Da, (calcd 5128.7 Da).

Synthesis of Cys-SUMO(47-93) 3:

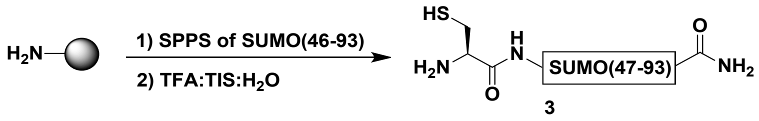

The SPPS of Cys-SUMO(47-93) was carried out on Rink amide resin, Fmoc- protection on preswollen resin was removed by treatment with 20% piperidine and the Gly93 of SUMO was coupled to Rink amide resin manually using 4 equiv of HCTU, 8 equiv of DIEA to the initial loading of the resin for 45 min (2 cycles). The remaining amino acids were coupled using peptide synthesizer in presence of 4 equiv of AA, 8 equiv of DIEA and 4 equiv of HCTU to the initial loading of the resin. The Dmb dipeptide Asp-Gly was manually coupled at position Asp63-Gly64 by using 2.5 equiv of the dipeptide Fmoc-Asp(O^{t}Bu)-(Dmb)Gly-OH with 2.5 equiv of HATU and 5 equiv of DIEA for 2 h. The coupling of the amino acids from Phe62 to Glu49 were carried out by double coupling. Cys at position 48 was mutated to Ser. Analytical cleavage was performed using regular cleavage cocktail to ensure complete couplings. Samples were analyzed using analytical

HPLC C18 column, with a gradient of 0-60%B over 30 min. Finally, the peptide was cleaved from the resin with the cleavage cocktail as mentioned above for Thz-SUMO(2-45)-Nbz and purified using preparative HPLC C18 column with a gradient of 0-60%B over 30 min. Overall yield 5% (26 mg).

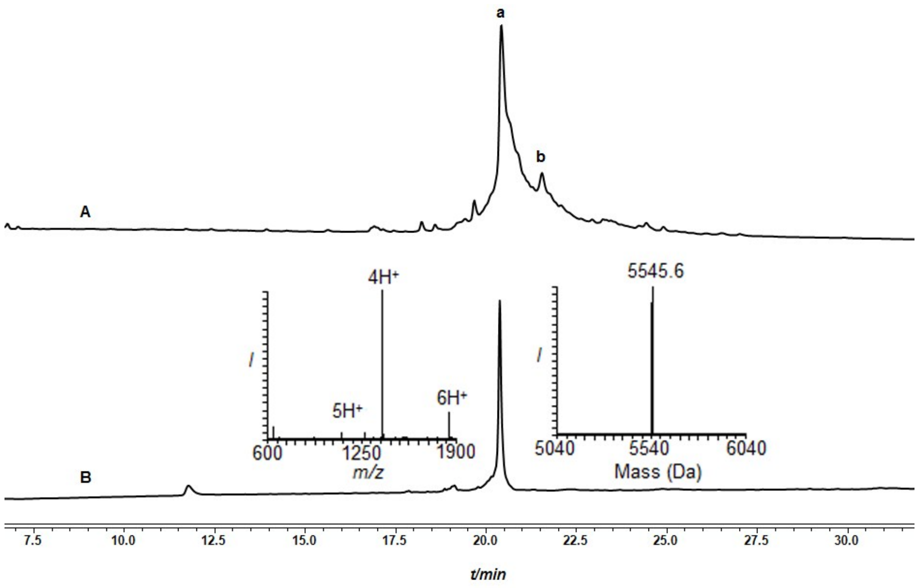

Figure S2: Synthesis of Cys-SUMO(47-93): A) Analytical HPLC of crude Cys-SUMO(47-93) peak a corresponds to Cys-SUMO(47-93) with observed mass 5545.6 ± 0.2 Da; calcd 5545.0 Da, peak b corresponds to unidentified mass. B) Analytical HPLC and mass analysis of purified Cys-SUMO(47-93).

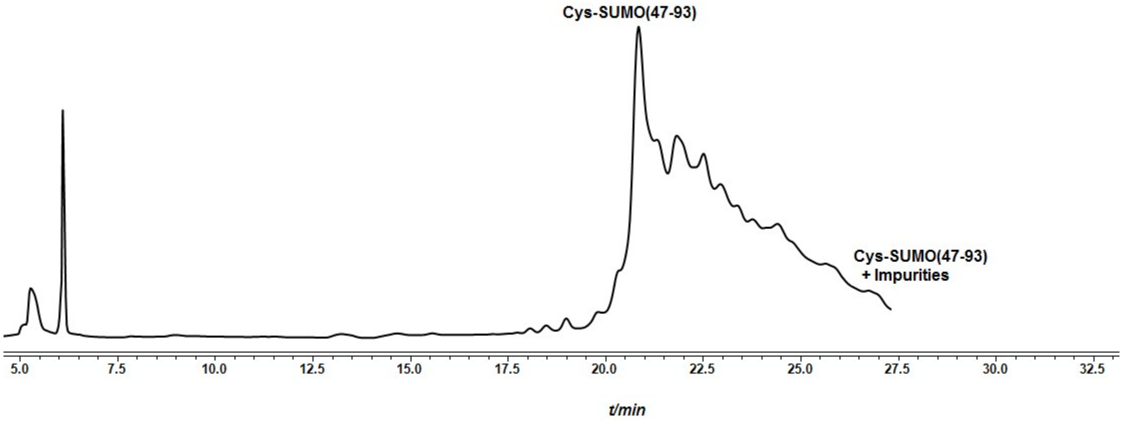

Figure S3: Preparative HPLC Cys-SUMO(47-93) in C18 column. The tail of the peak corresponds to major Cys-SUMO(47-93) with other peptide impurities of masses 3145 Da and 2520.

Synthesis of Cys-SUMO(2-93) 4:

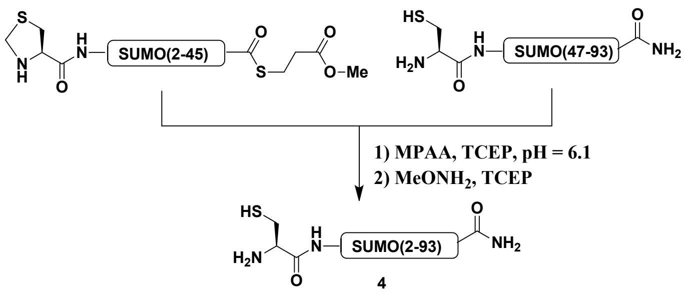

Thz-SUMO(2-45)-MMP (8.4 mg, 1.6×10^{-3} mmol) and Cys-SUMO(46-93) (10 mg, 1.8×10^{-3} mmol) were dissolved in 6 M Gn•HCl, 200 mM phosphate buffer (900 µL, 2 mM) containing MPAA (30 mg, 0.18 mmol) and TCEP (25 mg, 0.09 mmol) at pH 7.2. The reaction was kept at 37 °C for 3 h. After the completion of ligation, $MeONH_2$ (5.2 mg, 0.063 mmol) and TCEP (10.3 mg, 0.03 mmol) were added to the ligation mixture and the reaction was incubated at 37 °C for 12 h. The progress of the reaction was monitored by analytical HPLC using C4 column with a gradient of 5-55% B over 45 min. The product was purified using a C4 semi-preparative column and with the same gradient of solvent system to obtain Cys-SUMO(2-93) in 40 % yield (7.5 mg).

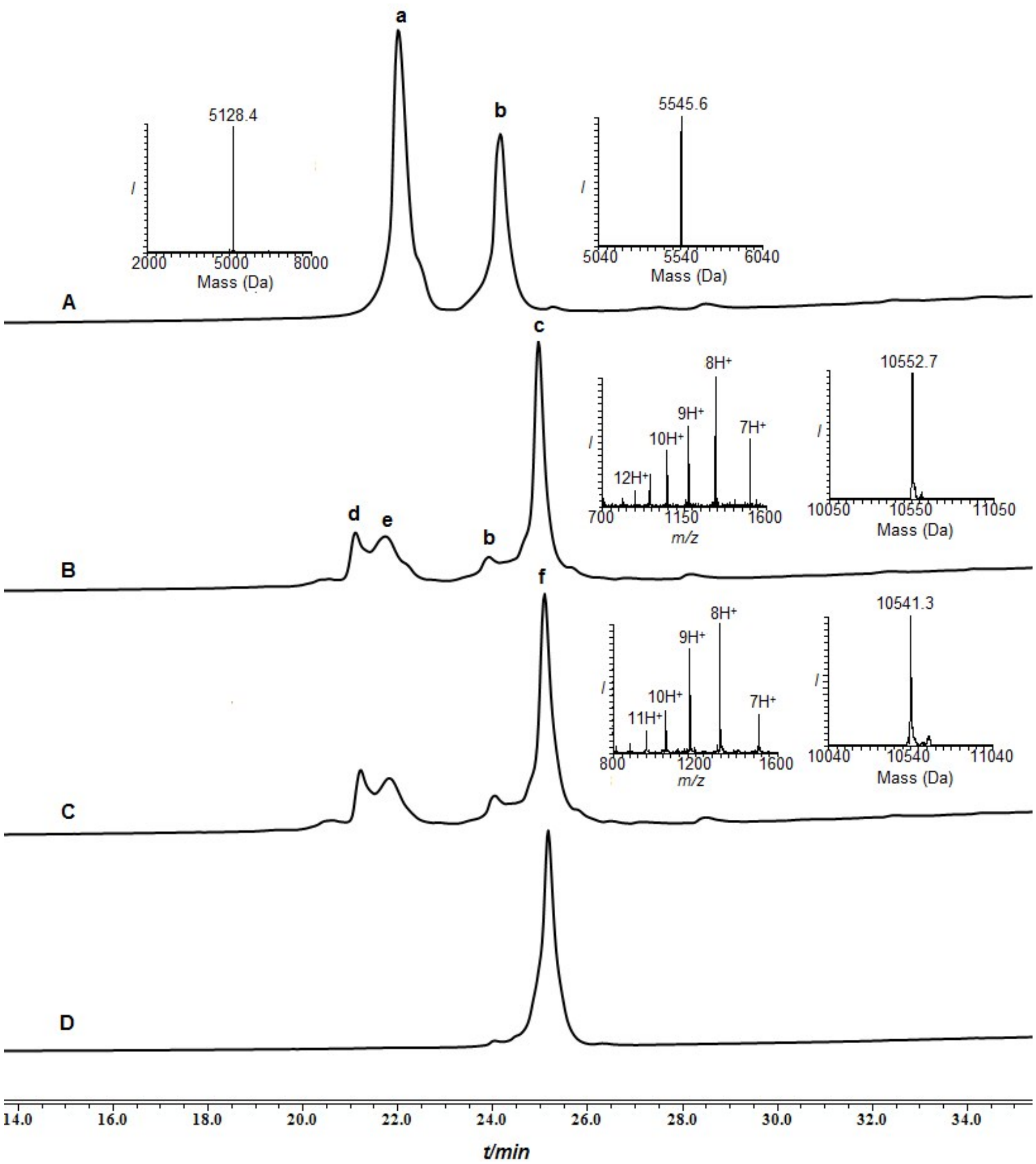

Figure S4: Synthesis of Cys-SUMO(2-93): A) Analytical HPLC and mass traces of the ligation between Thz-SUMO(2-45)-MMP and Cys-SUMO(47-93), peak a corresponds to Thz-SUMO(2-45)-MMP with observed mass 5128.4 ± 0.2 Da, calcd 5128.7 Da and peak b corresponds to Cys-SUMO(47-93) with observed mass 5545.6 ± 0.1 Da, calcd 5545.0 Da at time zero. B) Ligation after 3 h, peak c corresponds to desired ligation product Thz-SUMO(2-93) with the observed mass of 10552.7 ± 0.4 Da, calcd 10553.7 Da, peak d corresponds to hydrolyzed product Thz-SUMO(2-45)-COOH from Thz-SUMO(2-45)-MMP and peak e corresponds to Thz-SUMO(2-45)-CO-lactam. C) Thz-opening after 12h, peak f corresponds to thz-opened Cys-SUMO(2-93) with the observed mass of 10541.3 ± 0.2 Da, calcd 10541.7 Da. D) Analytical HPLC of purified thz-opened Cys-SUMO(2-93).

Synthesis of Cys-SUMO(2-93) 4: The ligation reaction was performed at pH 6.1 under the same molar concentrations of peptide fragments and reagents described above. The resultant Cys-SUMO(2-93) was isolated in 48% yield (9.2 mg).

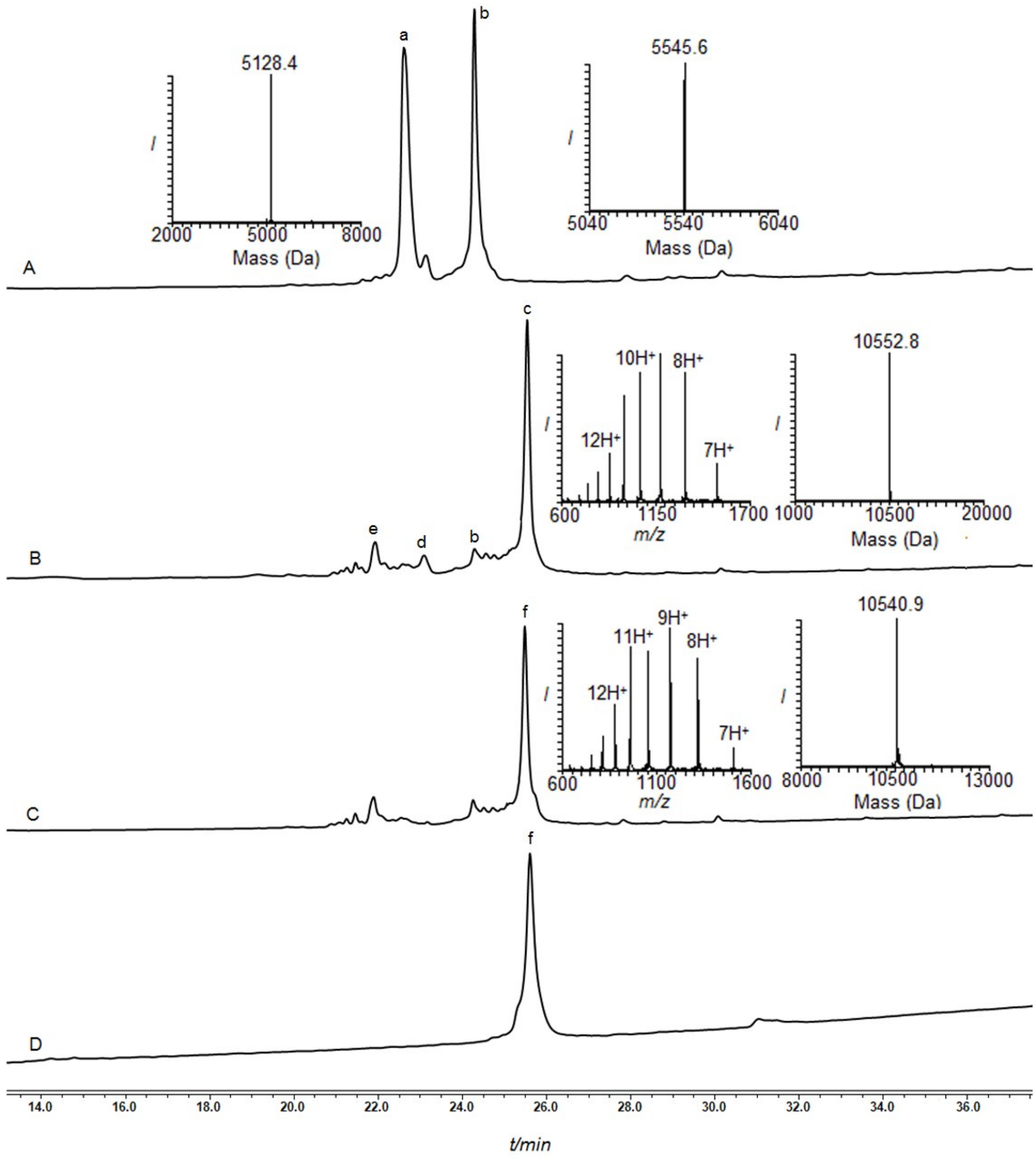

Figure S5: Synthesis of Cys-SUMO(2-93): A) Analytical HPLC and mass traces of the reaction between Thz-SUMO(2-45)-MMP and Cys-SUMO(47-93), peak a corresponds to Thz-SUMO(2-45)-MMP with the observed mass 5128.4 ± 0.2 Da; calcd 5128.7 Da and peak b corresponds to Cys-SUMO(47-93) with the observed mass of 5545.6 ± 0.1 Da; calcd 5545.0 Da at time zero; B) Ligation after 5 h, Peak c corresponds to the desired ligation product Thz-SUMO(2-93) with the observed mass of 10552.8 ± 0.4 Da, calcd 10553.7 Da, peak d corresponds to the hydrolyzed product Thz-SUMO(2-45)-COOH

from Thz-SUMO(2-45)-MMP and peak e corresponds to Thz-SUMO(2-45)-CO-lactam. C) Thz-opening after 12h, peak f corresponds to thz-opened Cys-SUMO(2-93) with the observed mass of 10540.3 ± 0.9 Da, calcd 10541.7 Da. D) Analytical HPLC of purified thz-opened Cys-SUMO(2-93).

Synthesis of Ub(K63*)-N-methyl-Cys:

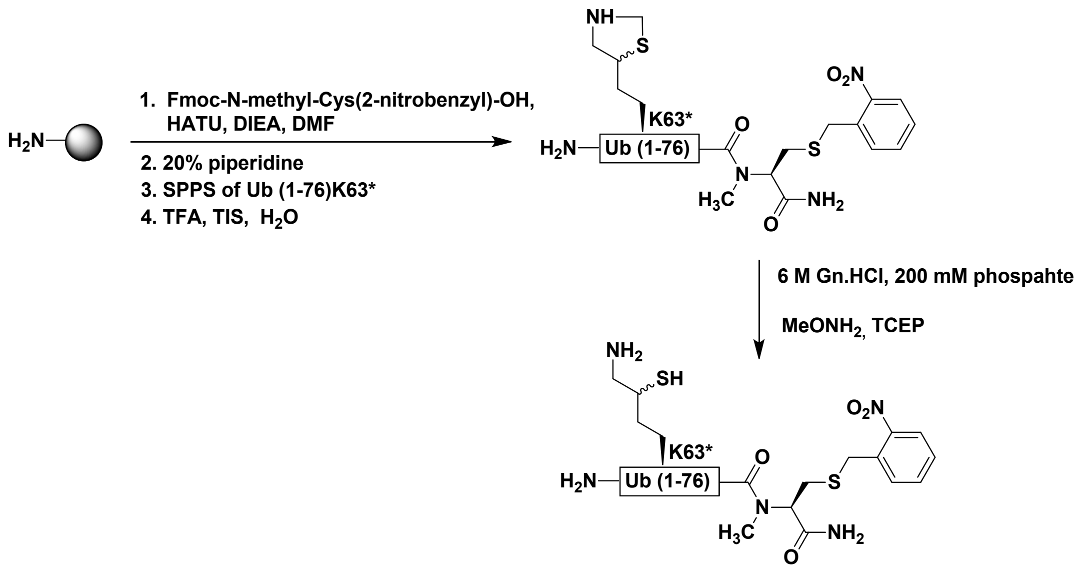

SPPS: The synthesis of peptide Ub(K63*)-N-methyl-Cys was carried out on Rink amide resin (0.44 mmol/g, 0.1 mmol scale). Fmoc-N-methyl-Cys(2-nitrobenzyl)-OH (4 equiv) was coupled to Rink amide resin manually using 4 equiv of HATU, 8 equiv of DIEA to the initial loading of the resin for 1 h. Fmoc deprotection was achieved by treatment of the resin with 20% piperidine for 3-7-3 min cycle. The first residue of Ub sequence (Gly76) was coupled using HATU (4 equiv) and DIEA (8 equiv) for 45 min (2 cycles). The remaining amino acids were coupled using peptide synthesizer in presence of 4 equiv of AA, 8 equiv of DIEA and 4 equiv of HCTU of the initial loading of the resin. The coupling was kept for 45 min and Fmoc-deprotection was achieved using 20% piperidine with 5 and 10 min cycles. Pseudoproline dipeptide Leu-Thr, Ile-Thr and Leu-Ser and Dmb dipeptide Asp-Gly were manually coupled, using 2.5 equiv to the resin initial loading for 2 h, at position Leu8-Thr9, Ile14-Thr15, Leu56-Ser57 and Asp52-Gly53 junctions using Fmoc-Leu-Thr($\psi^{Me,Me}$Pro)-OH, Fmoc-Ile-Thr($\psi^{Me,Me}$Pro)-OH, Fmoc-Leu-Ser($\psi^{Me,Me}$pro)-OH, and Fmoc-Asp(O^{t}Bu)-Dmb)Gly-OH respectively. Analytical cleavage was performed using cocktail containing TFA:TIS:H_2O (95:2.5:2.5) to ensure

complete coupling. The Thz (thiazolidine) protected δ-mercaptolysine was also manually coupled at position 63 using 1.5 equiv of this residue and HOBT-HBTU(2.5 equiv)/DIEA(5 equiv) to the initial loading of the resin. The coupling was performed for 2 h followed by analytical cleavage to ensure complete coupling. Met1 in ubiquitin was replaced with Nle1 to avoid oxidation during synthesis and handling.

Cleavage of the Ub(K63*)-N-methyl-Cys peptide from resin: The procedure used for Thz-SUMO(2-45)-Nbz in page **S6** was followed.

Thz-deprotection : The crude peptide (50 mg, 5.68×10^{-3} mmol) was dissolved in 6 M Gn•HCl (pH ~7) containing 200 mM phosphate buffer (1.8 mL, 3 mM). This mixture was treated with methoxylamine (14 mg, 0.17 mmol) and TCEP (24 mg, 0.085 mmol) at pH 4 for 12 h to unmask the δ- mercaptolysine. The reaction was followed using an analytical column. HPLC purification using a linear gradient of 0-60% B over 45 min afforded the Ub(K63*)-N-methyl-Cys in ~18 % yield (~9 mg).

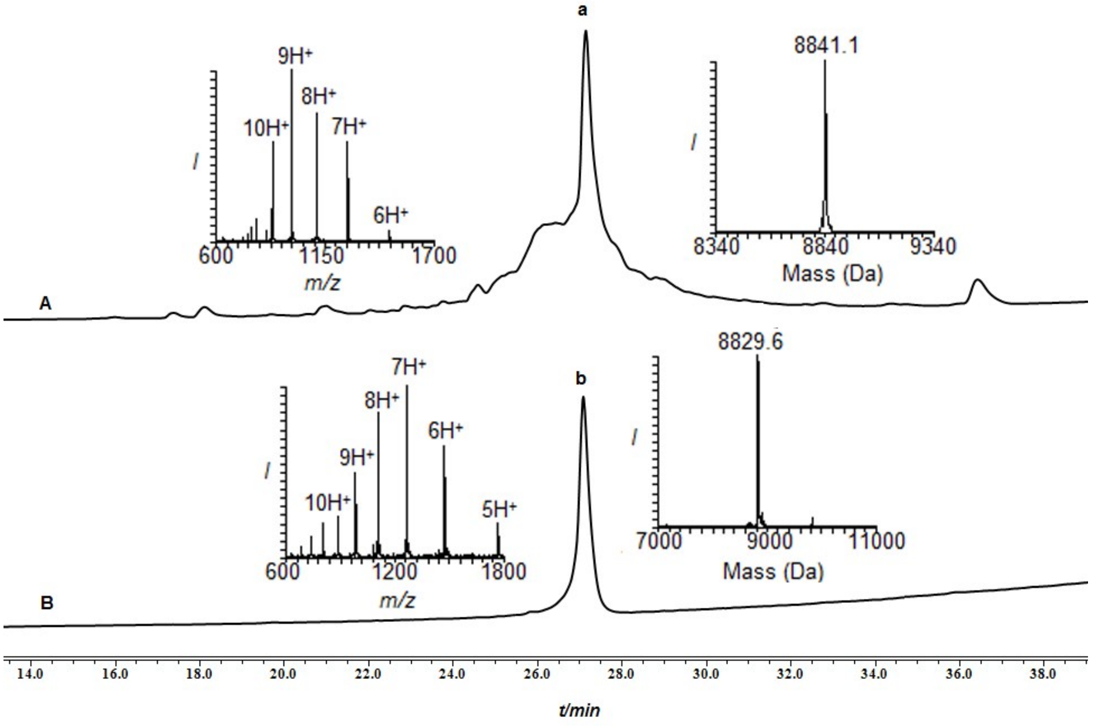

Figure S6: Analytical HPLC and mass traces of the synthesis of Thz opened Ub(K63*)-N-methyl-Cys: A) Peak a corresponds to the crude peptide of Thz containing Ub(K63*)-

N-methyl-Cys with the observed mass of 8841.1 ± 0.2, calcd 8841.8 Da. B) Peak b corresponds to the purified Thz opened Ub-(K63) N-methyl-Cys with the observed mass of 8829.6 ± 0.4 Da, calcd 8828.8 Da.

Synthesis of Ub-MPA (MPA: Mercapto propionic acid) 7:

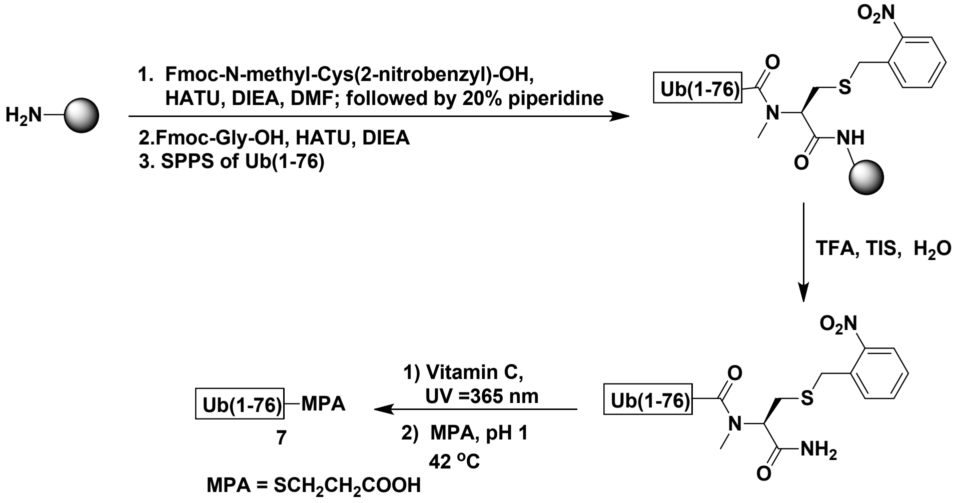

To synthesize Ub-MPA, we first obtained crude Ub-N-methyl-Cys using the above mentioned procedure for Ub(K63*)-N-methyl-Cys, excluding the incorporation of δ-mercaptolysine. Subsequently the crude peptide (50 mg, 5.71×10^{-3}) was dissolved in 6 M Gn•HCl buffer (1.8 mL, 3mM) containing 200 mM phosphate and vitamin C (10 mg, 0.056 mmol). The pH of the resultant reaction mixture was adjusted to ~7.0 followed by irradiation at UV-365 nm for 2 h with subsequent treatment with 20% (v/v) MPA at 42 °C for 20 h at pH 1 yielded Ub-MPA in 16% yield (8 mg).

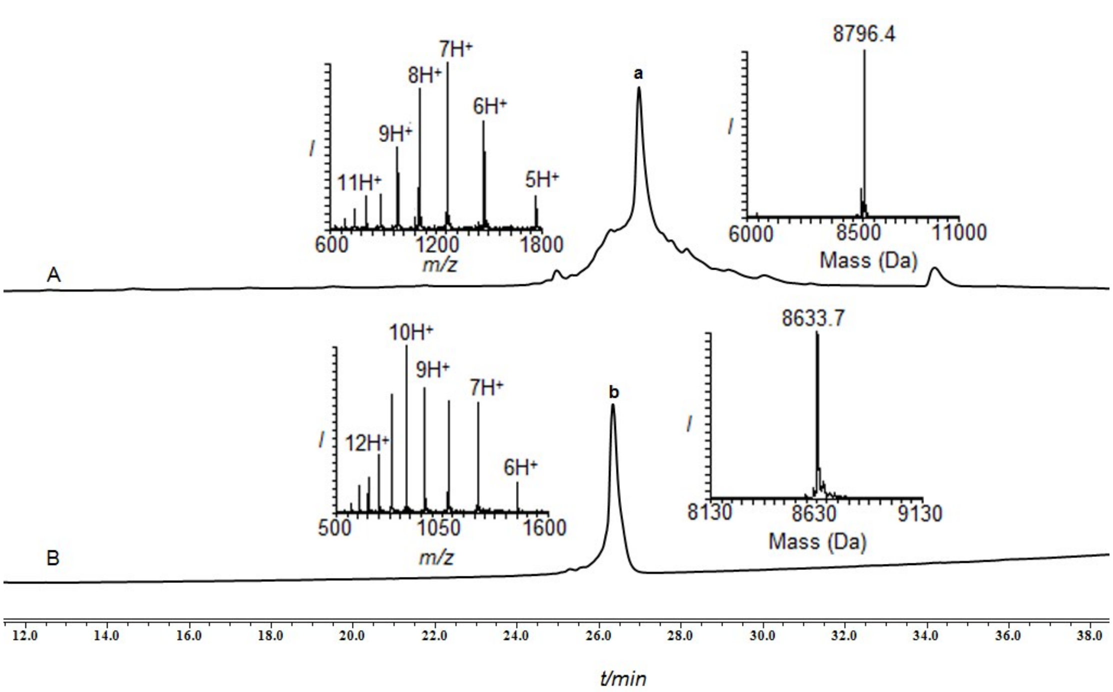

Figure S7: Analytical HPLC and mass traces of the synthesis of Ub-MPA: A) Peak a corresponds to the crude peptide of Ub-N-methyl-Cys with the observed mass of 8,796.4 ± 0.2 Da, calcd 8796.8 Da; B) Peak b corresponds to the purified Ub-MPA after switching N-methyl-Cys to MPA with the observed mass 8,633.7 ± 0.1 Da, calcd 8633.8Da.

Synthesis of di-Ub(K63*)-MPA:

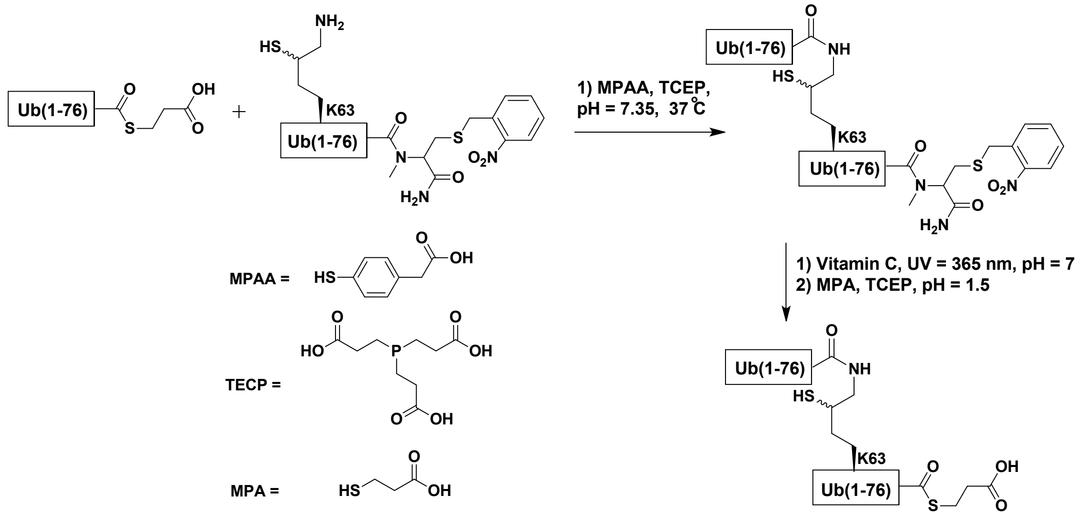

Ub(K63*)-N-methyl-Cys (9.1 mg, 1.05×10^{-3} mmol) and Ub-MPA (10 mg, 1.15×10^{-3} mmol) were dissolved in 6 M Gn•HCl, 200 mM phosphate buffer (580 µL, 2 mM)

containing MPAA (16.8 mg, 100 mM) and TCEP (14.3 mg, 50 mM) at pH 7 and the reaction mixture was kept at 37 ^{o}C for 4 h. The progress of the reaction was monitored by analytical HPLC (C4 column) and a gradient of 5-55% B over 40 min. After 4 h vitamin C (2 mg, 0.013 mmol) was added and the reaction was adjusted to pH ~7.0 followed by irradiation at UV-365 nm for 2 h at room temperature. Further, the mixture was treated with 20% (v/v) MPA at 42 ^{o}C for 20 h at pH 1.5. The progress of the reaction was followed using analytical HPLC (C4 column) with the gradient of 5-55% B over 40 min. For preparative HPLC, the same gradient was used to isolate the di-Ub(K63*)-MPA in 34% yield (~6.5 mg).

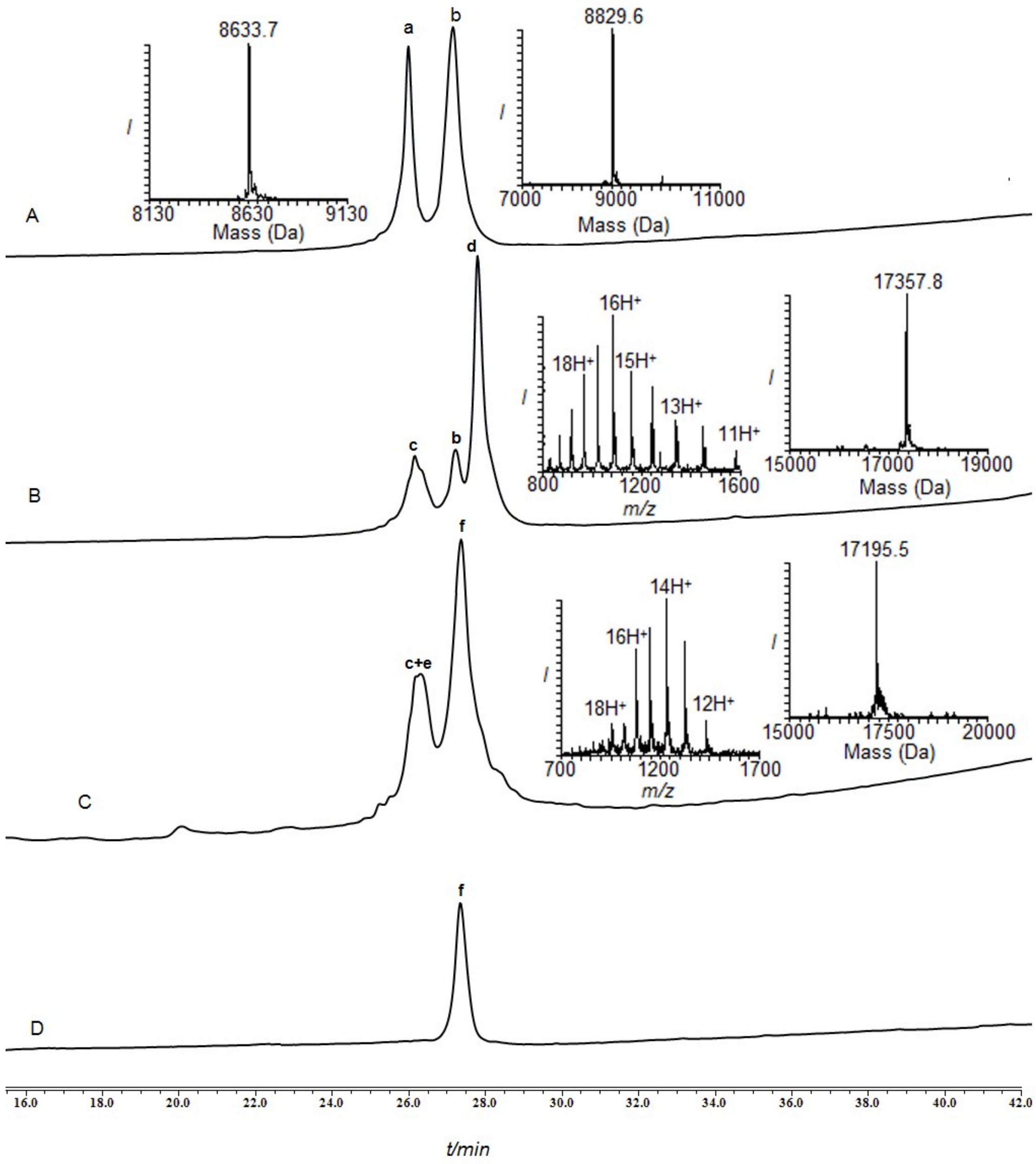

Figure S8. Synthesis of di-Ub(K63*)-MPA: A) Analytical HPLC and mass traces of the ligation between Ub-MPA and, peak a corresponds to Ub-MPA with the observed mass of 8,633.7 ± 0.1 Da, calcd 8633.8Da and peak b corresponds to Ub(K63)-N-methyl-Cys with the observed mass of 8829.6 ± 0.4 Da; calcd 8828.8 Da at time zero. B) Ligation after 4 h, peak c corresponds to hydrolyzed Ub-COOH from Ub-MPA; peak d corresponds to the desired ligation product with the observed mass 17357.8 ± 0.1 Da, calcd 17357.6. C) MPA switching after 16h, peak c+e corresponds to mixture of Ub-COOH and Ub(K63*)-MPA and peak f corresponds to di-Ub(K63*)-MPA with the observed mass of 17195.5 ± 0.3 Da, calcd 17194.6 Da). D) Analytical HPLC of purified di-Ub(K63*)-MPA.

Ligation reaction between Cys-SUMO(2-93) 4 and di-Ub(K63*)-MPA:

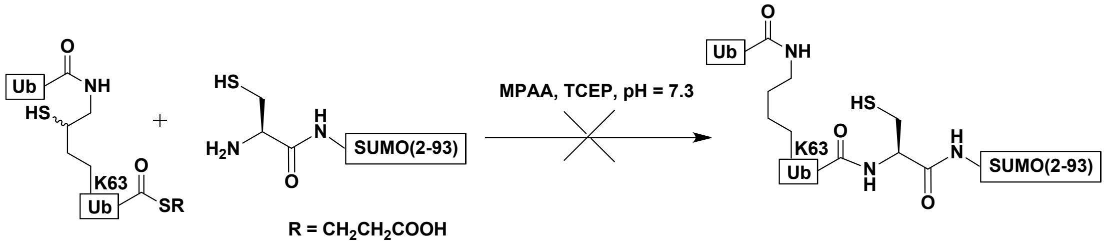

Cys-SUMO(2-93) (4.0 mg, 3.8×10^{-4} mmol) and di-Ub(K63*)-MPA (5 mg, 2.90×10^{-4} mmol) were dissolved in argon purged 6 M Gn·HCl, 200 mM Na_2HPO_4 buffer (260 μL, 1 mM) containing MPAA (4.4mg, 0.026 mmol) and TCEP (3.6mg, 0.0128 mmol) at pH ~7.3. The reaction was incubated at 37 °C for 10 h. The progress of the reaction was monitored using analytical HPLC and C4 column with a gradient of 5-55% over 45 min.

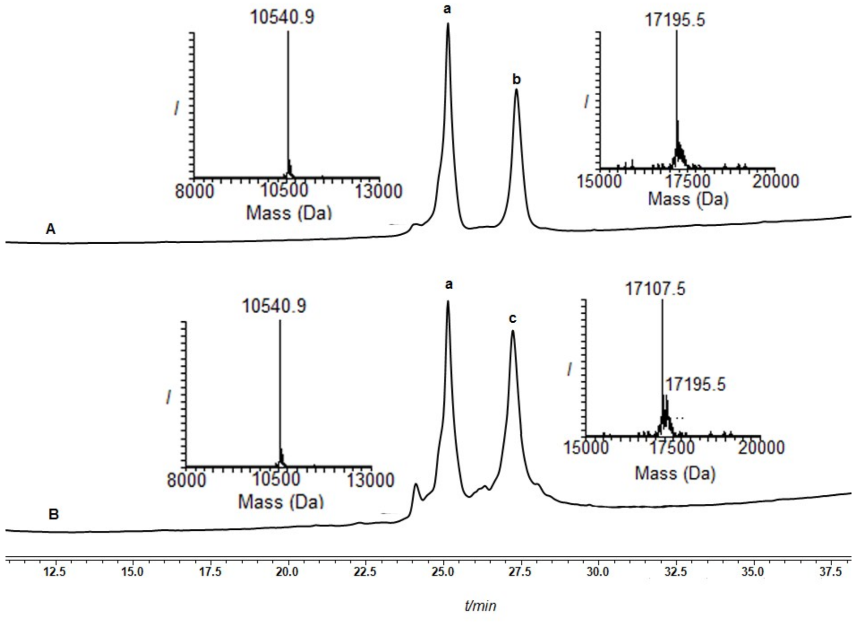

Figure S9. Ligation between di-Ub(K63*)-MPA and Cys-SUMO(2-93): A) Analytical HPLC and mass traces of the ligation between Cys-SUMO(2-93) and di-Ub(K63*)-MPA, peak a corresponds to Cys-SUMO(2-93) with the observed mass of 10540.9 ± 0.6 Da, calcd mass 10541.7 Da and peak b corresponds to di-Ub(K63*)-MPA with the observed mass of 17195.5 ± 0.5 Da, calcd 17194.6 Da at time zero. B) Ligation after 24 h, peak c corresponds to hydrolyzed di-Ub(K63*)-COOH along with unreacted di-Ub(K63*)-MPA with the observed mass 17075.5 Da and 17195.5 Da.

Synthesis of Ub(K63*)-MPA 5:

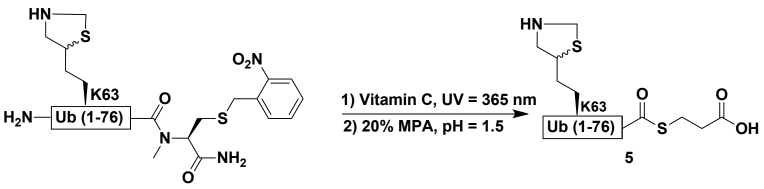

The crude Ub(K63*)-N-methyl-Cys (50mg, 5.6×10^{-3} mmol) was dissolved in 6 M Gn•HCl (1.8 mL, 3 mM) buffer, containing 200 mM phosphate and vitamin C (9.6 mg, 0.052 mmol) and irradiated with UV-365 nm for 2 h. To this mixture MPA was added (20% v/v) and the reaction was kept at 42 ^{o}C, pH ~1.5 for 16 h. HPLC purification using a linear gradient of 5-55% B over 40 min afforded the corresponding thioester in ~16% isolated yield (8 mg).

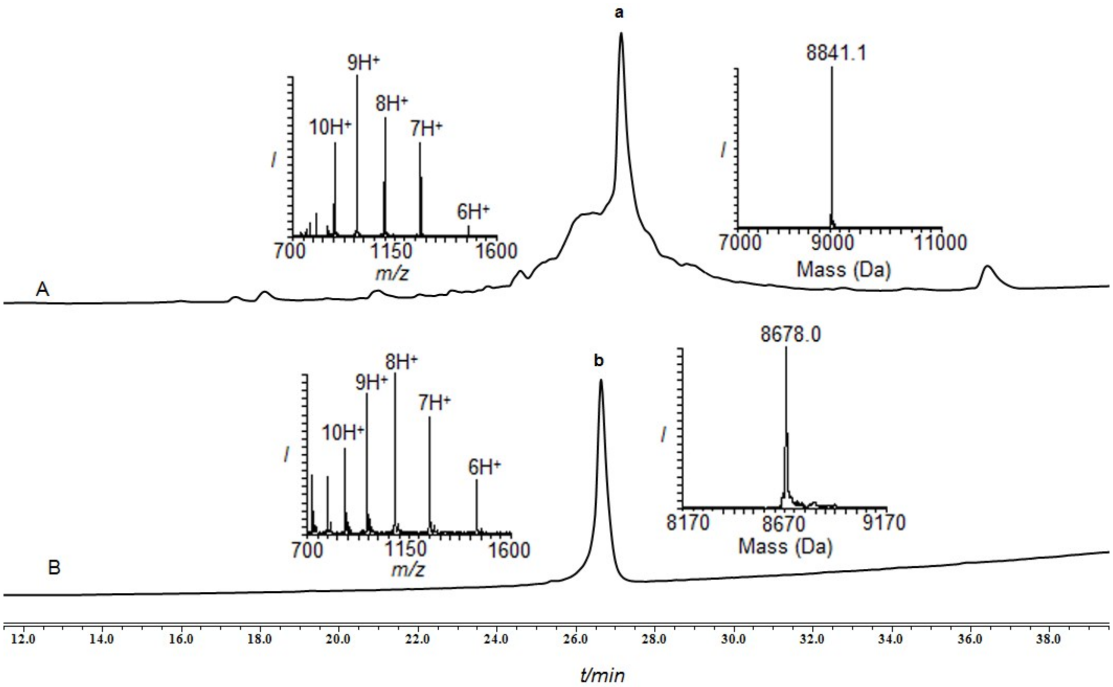

Figure S10. Synthesis of Ub(K63*)-MPA: A) Peak a corresponds to the crude peptide Ub(K63*)-N-methyl-Cys with the observed mass of 8,841.1 ± 0.2 Da, calcd 8,840.8 Da; B) Peak b corresponds to the MPA switched purified product Ub(K63*)-MPA with the observed mass 8,678.0 ± 0.1 Da, calcd 8,677.8 Da.

Synthesis of Ub(K63*)-Cys-SUMO(2-93) 6:

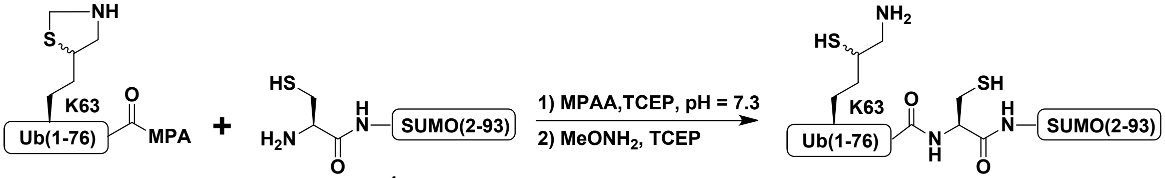

5 4 6

Ub(K63*)-MPA (9.0 mg, 1.04×10^{-3}mmol) and Cys-SUMO(2-93) (10 mg, 9.4×10^{-4} mmol) were dissolved in 6 M Gn•HCl, 200 mM Phosphate buffer, (0.95 ml, 1 mM) containing MPAA (16 mg, 0.094 mmol) and TCEP (12.7 mg, 0.047 mmol) at pH 7.3. The reaction mixture was kept at 37 °C for 3 h. This was followed by the addition of $MeONH_2$ (2.7 mg, 0.03 mmol) and TCEP (5.4 mg, 0.018 mmol) and the reaction was incubated at 42 °C for 12 h. The progress of the reaction was monitored by analytical HPLC using C4 column with a gradient 5-55% B over 40 min. the same gradient was used to isolate the Ub(K63*)-Cys-SUMO(2-93) **6** in 25% yield (5.2 mg).

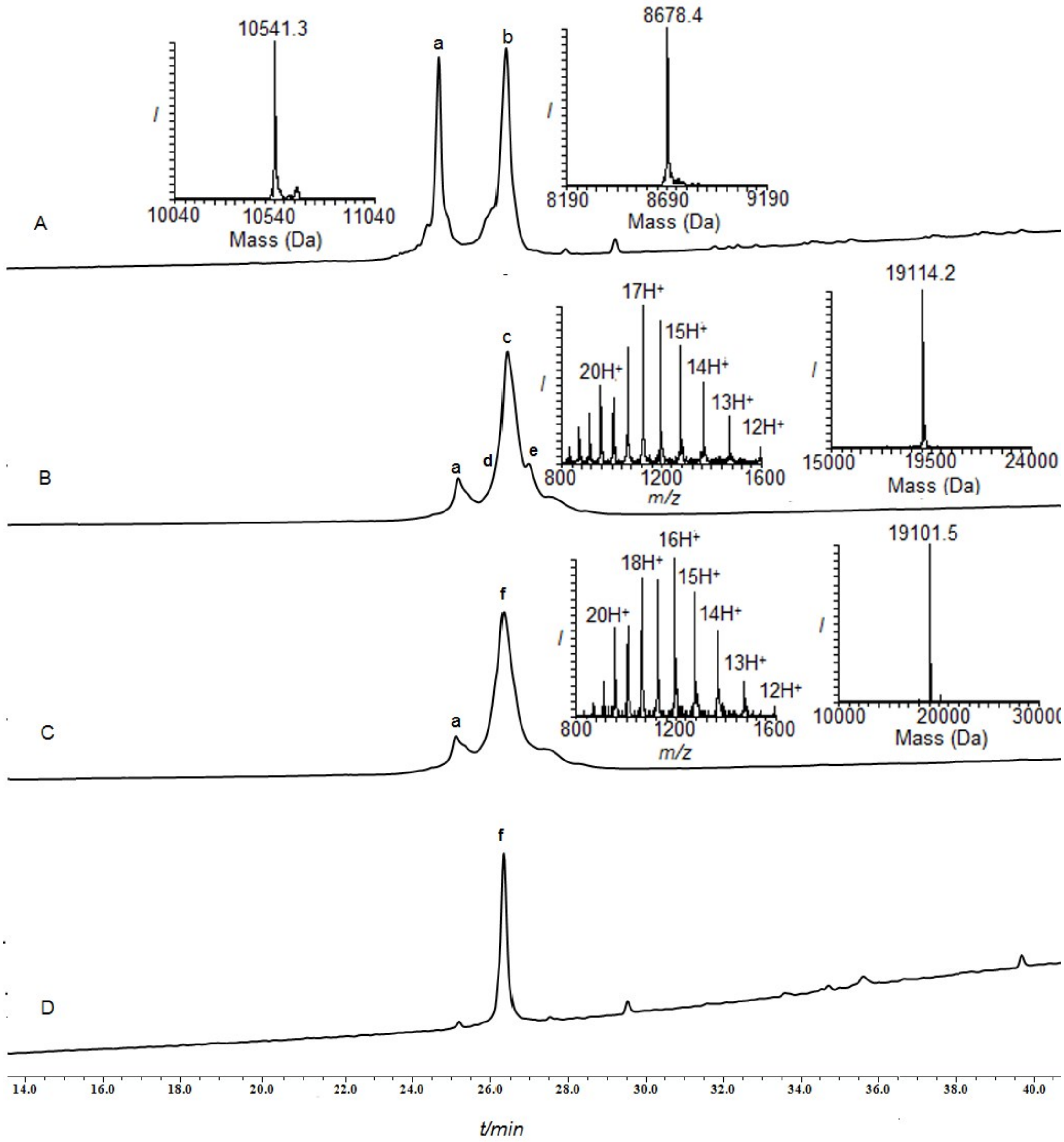

Figure S11: Synthesis of Ub(K63*)-Cys-SUMO(2-93): A) Analytical HPLC and mass traces of the ligation between Cys-SUMO(2-93) and Ub(K63*)-MPA, peak corresponds to with the observed mass of 10,541.3 ± 0.3 Da, calcd 10,541.7 Da and peak b corresponds to Ub(K63*)-MPA with the observed mass of 8,678.4 ± 0.4 Da, calcd 8,677.8 Da at time zero. B) Ligation after 3 h, peak c corresponds to desired ligation product Ub(K63*)-Cys-SUMO(2-93) with the observed mass of 19,114.2 ± 0.2 Da , calcd 19,114.5 Da, peak d corresponds to the Ub(K63*)-COOH, peak e corresponds to unidentified mass. C) Thz-opening after 12h, peak f corresponds to thz-opened Ub(K63*)-Cys-SUMO(2-93) with the observed mass of 19,101.5 ± 0.5 Da, calcd 19102.5 Da. D) HPLC analysis of purified thz-opened Ub(K63*)-Cys-SUMO(2-93).

Synthesis of Ub(K63*)-Cys-SUMO(2-93) 6:

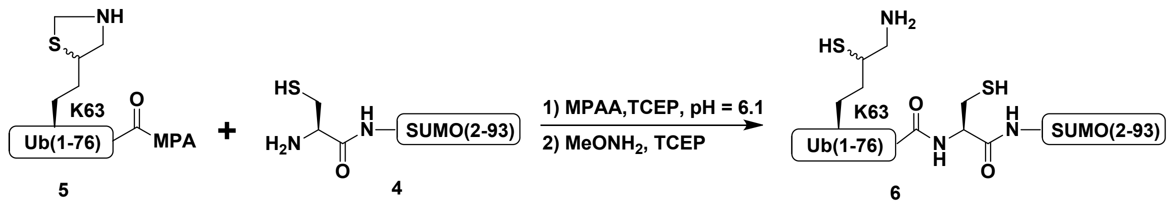

The reaction was performed at pH 6.1 under same molar concentrations of substrates and reagents as described before. The compound SUMO-Ub(K63*) **6** was isolated in 30 % yield (6 mg).

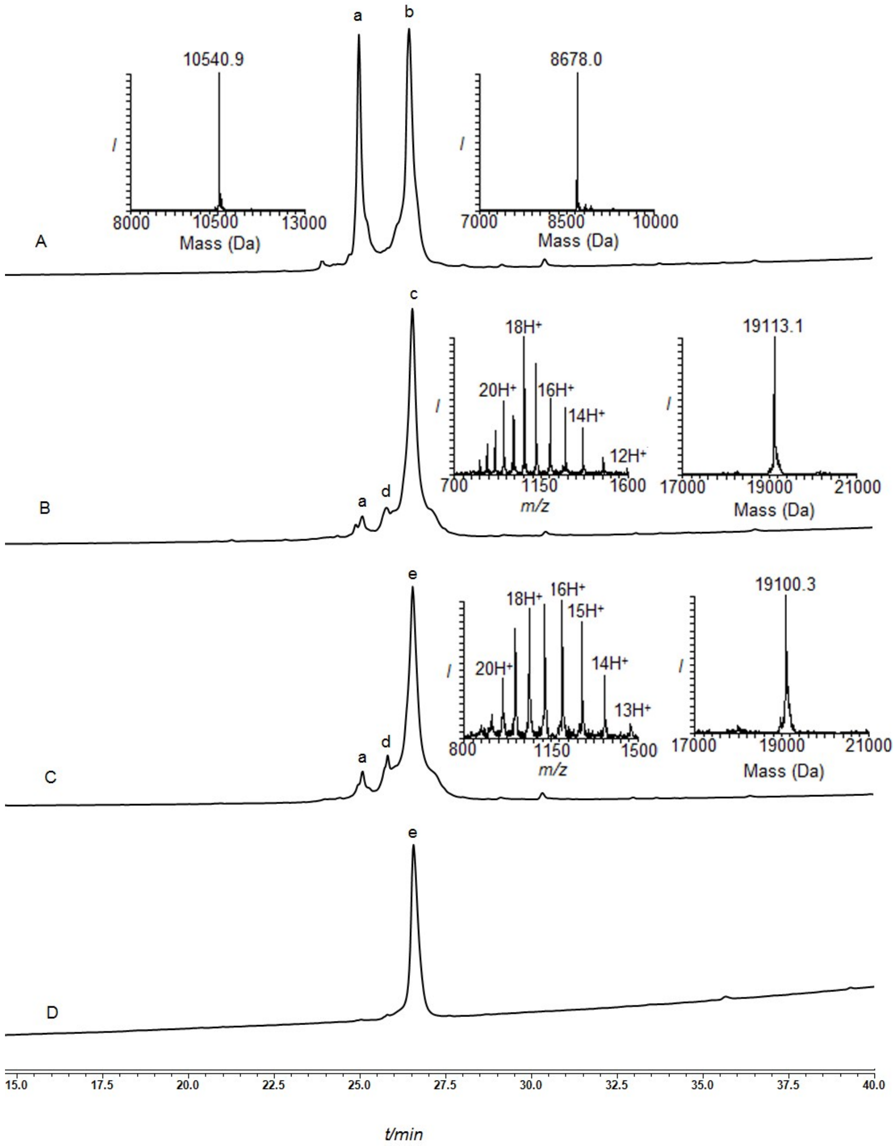

Figure S12: Synthesis of Ub(K63*)-Cys-SUMO(2-93): A) Analytical HPLC and mass traces of the ligation between Cys-SUMO(2-93) and Ub(K63*)-MPA, peak a corresponds to Cys-SUMO(2-93) with the observed mass of 10,540.3 ± 0.5 Da, calcd 10,541.7 Da and peak b corresponds to Ub(K63*)-MPA with the observed mass of 8,678.0 Da; ± 0.4 calcd 8,677.8 Da at time zero. B) Ligation after 5 h, peak c corresponds to desired ligation product Ub(K63*)-Cys-SUMO(2-93) with the observed mass of 19,113.1 ± 0.7 Da, calcd 19,114.5 Da, peak d corresponds to the Ub(K63*)-COOH; C) Thz-opening after 12h, peak e corresponds to thz-opened Ub(K63*)-Cys-SUMO(2-93) with the observed mass of 19,100.3 ± 0.9 Da, calcd 19102.5 Da. D) HPLC analysis of purified thz-opened Ub(K63*)-Cys-SUMO(2-93).

Synthesis of diUb(K63)-Ala-SUMO(2-93) 1:

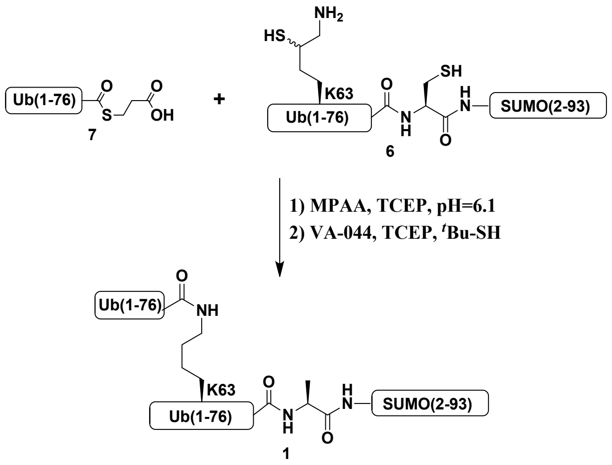

Ub(K63*)-Cys-SUMO(2-93) (5 mg, 2.6×10^{-4} mmol) and Ub-MPA (2.7 mg, 2.6×10^{-4} mmol) were dissolved in 6 M Gn•HCl, 200 mM phosphate buffer (260 µL, 1mM) containing MPAA (4.3 mg, 0.026 mmol) and TCEP (3.7 mg, 0.013 mmol) at pH 6.1. The reaction was incubated at 37 °C for 7 h. The progress of the reaction was monitored by analytical HPLC with a gradient of 5-55% over 40 min using C4 column. After completion of the reaction, the mixture was dialyzed in a Slide-A-Lyzer 3.5K dialysis cassette (Thermo scientific, 0.1-0.5 mL) in 6 M Gn•HCl, 200 mM phosphate buffer (500 mL) for overnight and then subjected to radical desulfurization by adding TCEP (35.7 mg, 250 mM), VA-044 (7.6 mg, 0.023 mmol) and *tert*-butyl thiol (50 µL) and incubated at 37 °C for 6 h. Progress of the reaction was monitored using analytical HPLC C4 column with a gradient of 5-55% B over 40 min. For semi-preparative HPLC, the same

gradient was used to isolate the product di-Ub(K63)-Ala-SUMO(2-93) **1** in ~20% yield (1.5mg).

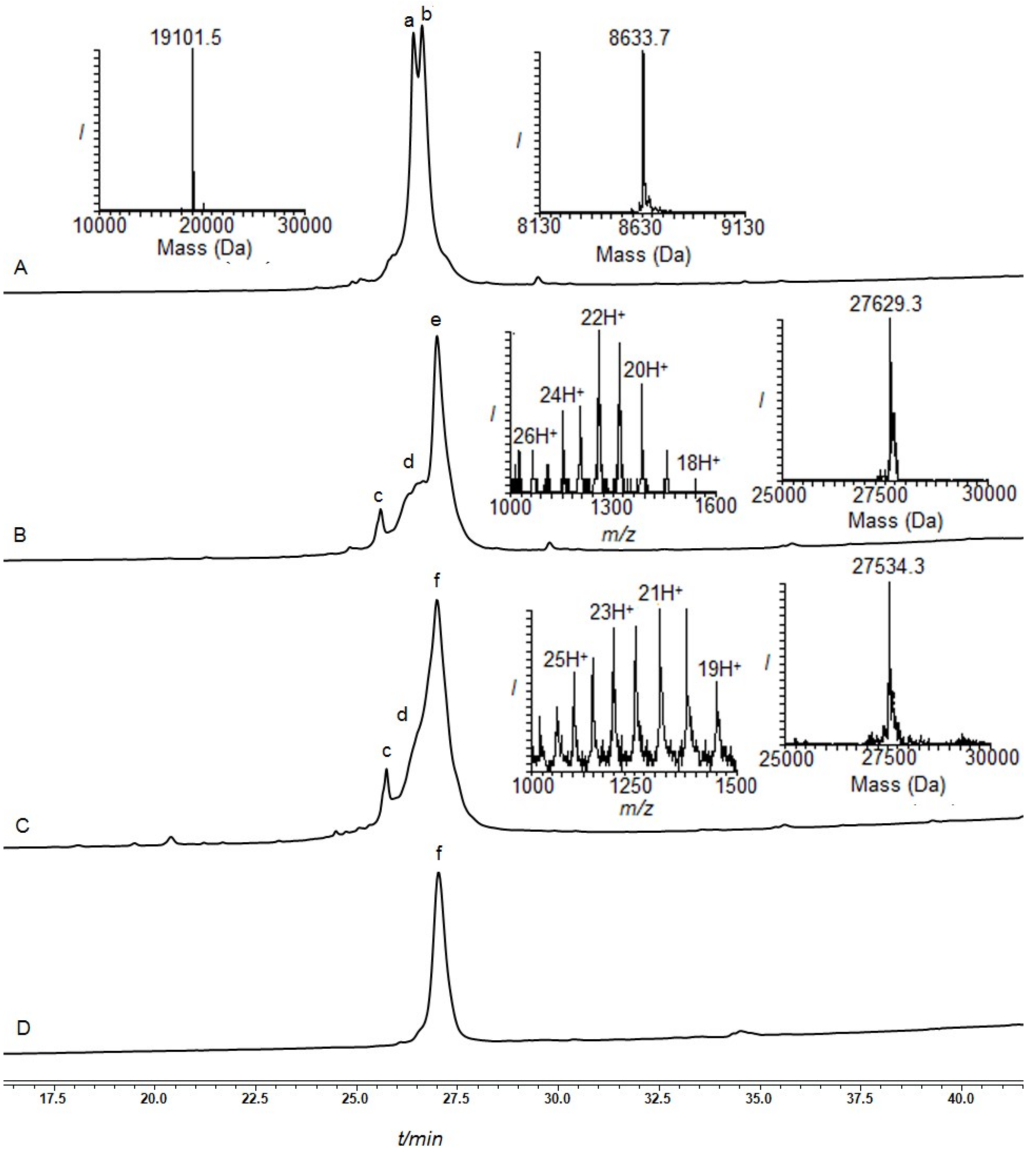

Figure S13: Synthesis of di-Ub(K63)-Cys-SUMO(2-93): A) Analytical HPLC and mass traces of the ligation between Ub(K63*)-Cys-SUMO(2-93) and Ub-MPA, peak a corresponds to Ub(K63*)-Cys-SUMO(2-93) with the observed mass of 19,101.5 ± 0.6 Da, calcd 19,102.5 Da, and peak b corresponds to Ub-MPA with the observed mass of 8,633.7 ± 0.1 Da, calcd 8633.8 Da at time zero. B) Reaction after 7 h, peak c corresponds to unidentified mass, peak d corresponds to the Ub-COOH, peak e corresponds to the desired ligation product di-Ub(K63*)-Cys-SUMO(2-93) with the observed mass of 27,629.3 ± 1.1 Da, calcd 27,631.3 Da. C) Desulfurization after 8h: peak f corresponding to the desired di-Ub(K63*)-Ala-SUMO(2-93) with the observed mass of 27,534.3 ± 0.9 Da, calcd 27535.2 Da. D) Analytical HPLC analysis of di-Ub(K63)-Cys-SUMO(2-93).

Synthesis of model peptide SUMO(49-93)-Dbz-(Arg)$_6$ 10:

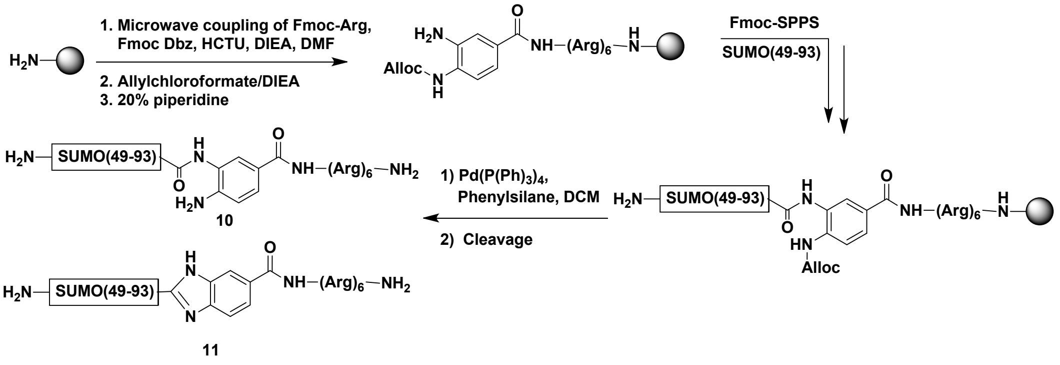

The SPPS of SUMO(49-93)-Dbz-(Arg)$_6$ was carried out on Rink amide resin, Fmoc-protection on preswollen resin was removed by treating with 20% piperidine and all the six Arg and Fmoc-Dbz were coupled (2 cycles) on microwave at 75 ^oC (20 Watt) for 5 min each with 4 equiv of AA, 4 equiv of HCTU and 8 equiv of DIEA. Then, the resin was washed with DMF and DCM, followed by treatment with allyl chloroformate (540μL, 50 equiv) in 3mL of dry DCM for overnight to protect the free amine of Dbz with Alloc. After this the Fmoc was removed from the Dbz and Gly93 of SUMO was coupled(2 cycles) manually on Dbz using 10 equiv of HATU, 20 equiv of DIEA to the initial loading of the resin for 45 min (2 cycles). The remaining amino acids were coupled using peptide synthesizer in presence of 4 equiv of AA, 4 equiv of HCTU and 8 equiv of DIEA of the initial loading of the resin. The Dmb dipeptide Asp-Gly was manually coupled at position Asp63-Gly64 by using 2.5 equiv the dipeptide Fmoc-Asp(OtBu)-(Dmb)Gly-OH with 2.5 equiv of HATU and 5 equiv of DIEA for 2h. The coupling of the amino acids from Phe62 to Glu49 were carried out by double coupling. Cys48 was mutated to Ser. Analytical cleavage was performed using regular cleavage cocktail to ensure complete coupling. Samples were analyzed using analytical HPLC C18 column, with a gradient of 0-60% B over 30 min. After completing the SPPS the resin was washed with DMF and DCM and Alloc deprotection on Dbz was carried out by treating the peptide (0.1 mmol) with a mixture of $Pd(PPh_3)_4$ (24 mg, 0.02 mmol) and phenyl silane (240 μL, 2.2 mmol) in 3 mL of dry DCM for 1 h. Finally, the peptide was cleaved from the resin with the cleavage cocktail as mentioned above for Thz-SUMO(2-45)-Nbz

and purified using preparative HPLC C18 column with a gradient of 0-60% B over 30 min.

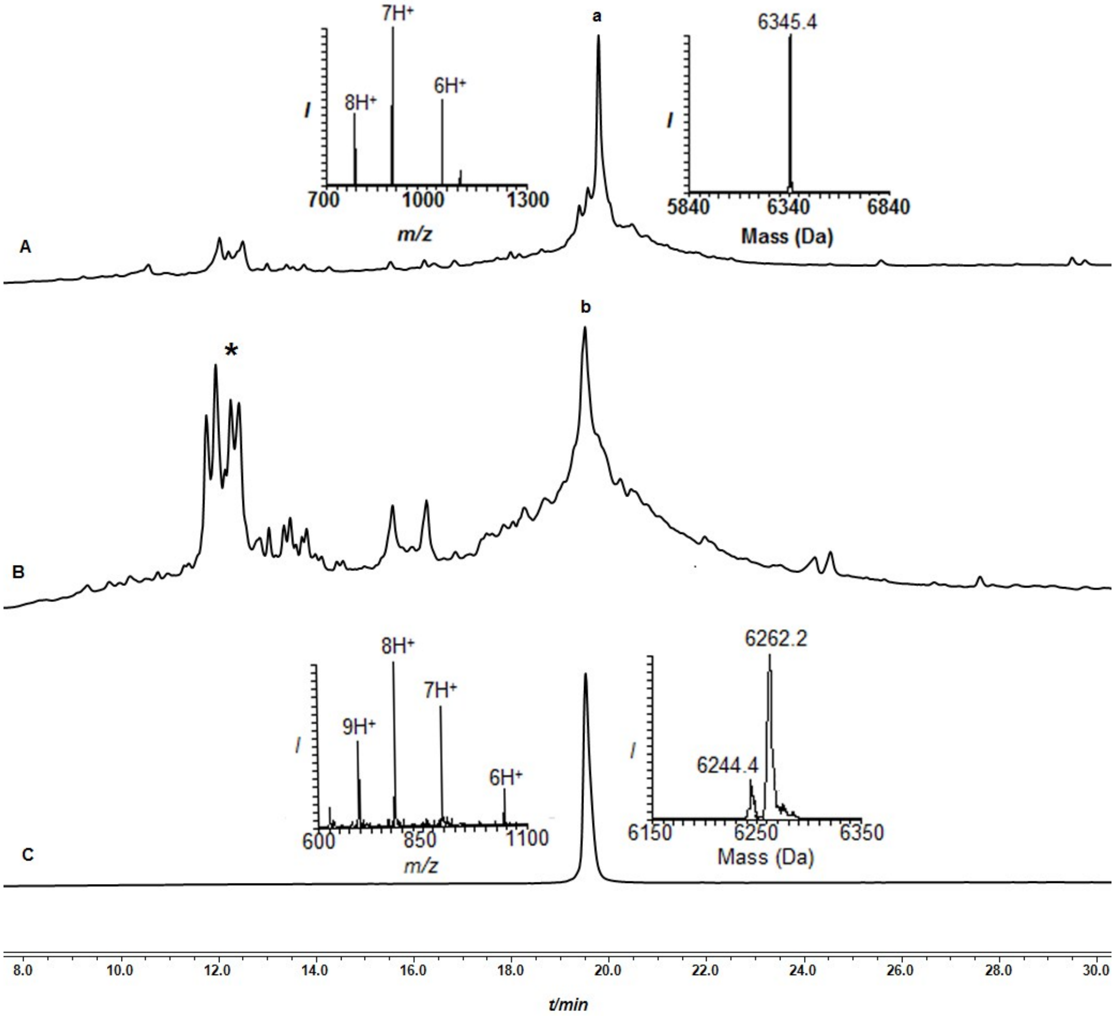

Figure S14. Synthesis of SUMO(49-93)-Dbz-$(Arg)_6$ **10** using alloc protection on Dbz; A) Crude HPLC and mass analysis of SUMO(49-93)-Dbz(Alloc)-$(Arg)_6$: peak a corresponds to SUMO(49-93)-Dbz(Alloc)-$(Arg)_6$ with observed mass 6345.4 ± 0.8 Da, calcd 6347.7. B) Analytical HPLC and mass traces of the crude SUMO(49-93)-Dbz-$(Arg)_6$ after alloc removal: peak b corresponds to the SUMO(49-93)-Dbz-$(Arg)_6$ (**10**) with the observed mass 6262.2 ± 0.2 Da, calcd 6262.7 Da and (~ -18 Da Mass) 6244.4 Da. C) HPLC analysis of purified SUMO(49-93)-Dbz-$(Arg)_6$ **10**.

Cleavage of Dbz-(Arg)$_6$ from SUMO(49-93)-Dbz-(Arg)$_6$:

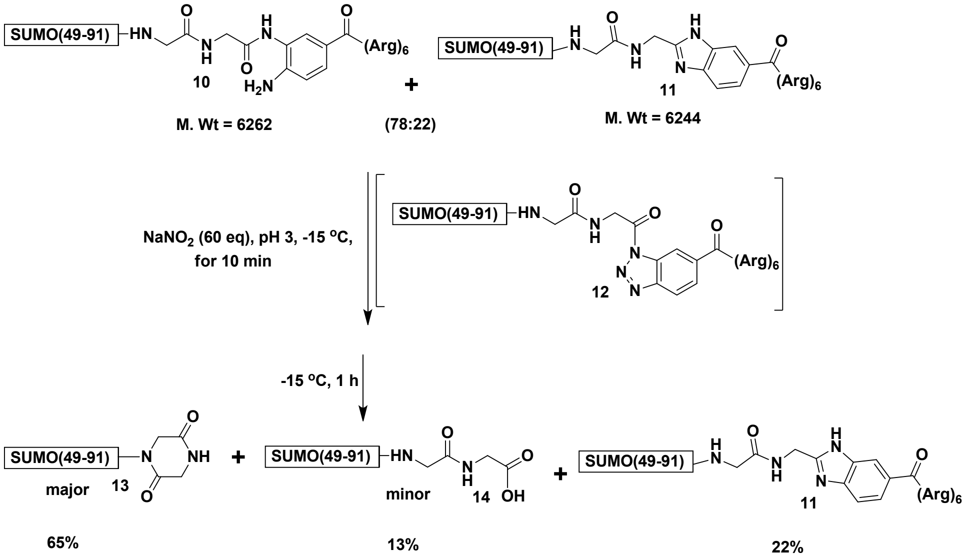

2 mg of SUMO(49-93)-Dbz-(Arg)$_6$ (3.1×10^{-4} mmol) was dissolved in 160 µL of 6 M Gn•HCl, 200 mM phosphate buffer solution containing 120 mM $NaNO_2$ at pH 3 (-15 °C). The reaction was kept at the same temperature for 1 h. Aliquots were taken after every 10 min and analyzed using HPLC and mass spectrometry to follow the progress of the reaction.

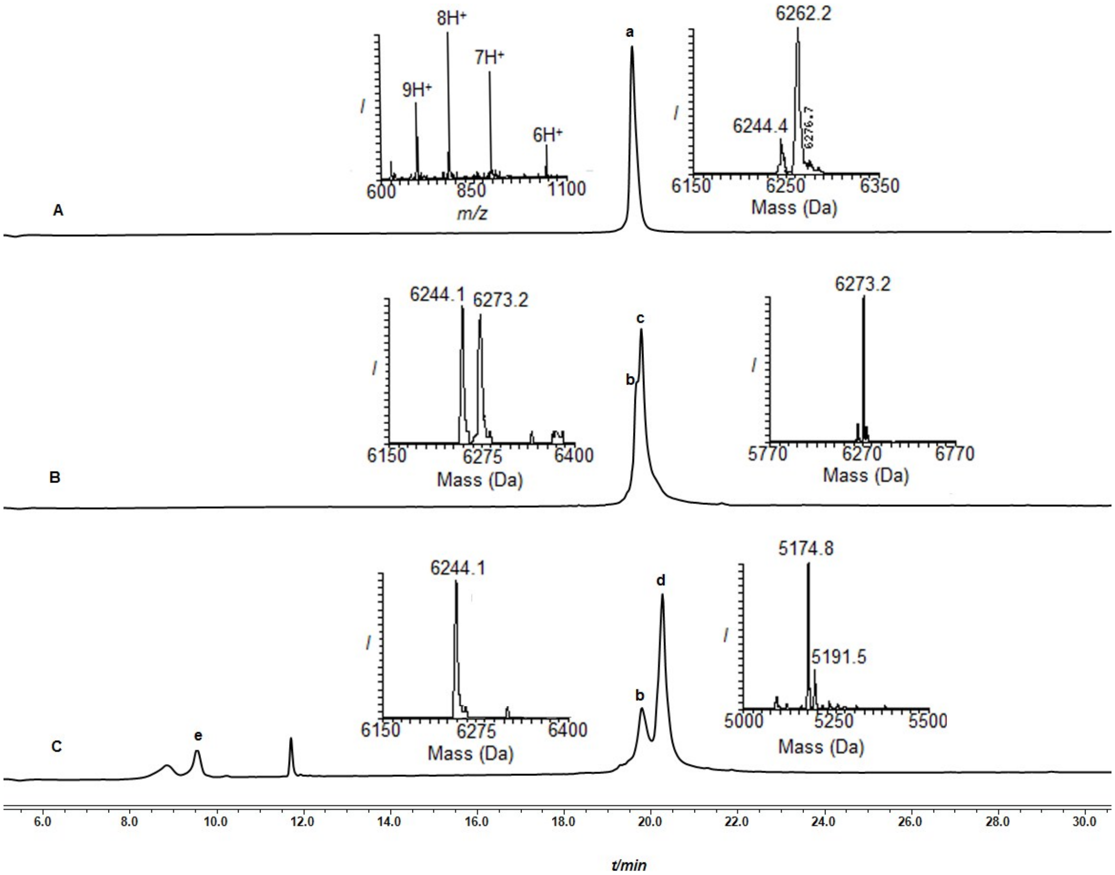

Figure S15: Hydrolysis of SUMO(49-93)-benzimidazole/SUMO(49-93)-Dbz-$(Arg)_6$; A) Peak a corresponds to the mixture of SUMO(49-93)-benzimidazole/SUMO(49-93)-Dbz-(Arg) with the observed masses 6244.4/6262.2, respectively. B) After 10 min of $NaNO_2$ treatment: peak b corresponds to the mixture of SUMO(49-93)-benzimidazole (**11**)/ SUMO(49-93)-benzotriazole (**12**) with the observed masses 6244.1 and 6273.2, respectively; peak c corresponds to SUMO(49-93)-benzotriazole with the observed mass 6273.2 Da ± 0.2; calcd 6273.7 Da. C) After 70 min. of $NaNO_2$ treatment; peak b corresponds to the unreactive benzimidazole (**11**) with the observed mass of 6244.1 ± 0.3 Da, calcd 6244.7 Da from the starting material, peak d corresponds to the minor amount of desired hydrolyzed product SUMO(47-93)-COOH (**14**) with the observed mass 5191.5 ± 0.5 Da calcd 5192.6 and major amount of undesired side product SUMO-(47-91)-piperazine-2,5-dione (**13**) with observed mass 5174.8 ± 0.1 Da calcd 5174.6, peak e corresponds to the benzotriazole containing the Arg tag.

Synthesis of SUMO(49-93)-Dbz-6(Arg) 10 by using proc protection:

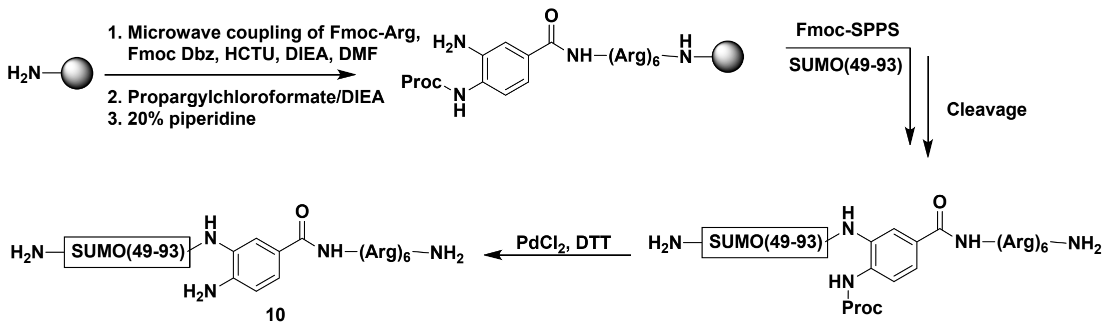

The Proc protected model peptide was synthesized in a similar manner to the Alloc protected peptide. After elongation, the peptide was cleaved and lyophilized. The Proc removal was carried out by dissolving the crude peptide (30 mg, 0.004 mmol) in 6 M Gn•HCl, 200mM phosphate buffer (1.1 mL, 2 mM), followed by the addition of $PdCl_2$ (16 mg, 0.094 mmol) in argon purged 6 M Gn•HCl, 200 mM Na_2HPO_4 buffer ($PdCl_2$ takes ~30 min to dissolve at 37 ^oC). The reaction was kept at 37 ^oC for 30 min. The progress of the reaction was monitored by analytical HPLC with the gradient 0-60% B over 30 min. Subsequently, the reaction mixture was treated with (36 mg, 0.236 mmol, ditiothreitol (DTT) to quench and precipitate the palladium from the reaction mixture. The reaction mixture was centrifuged and supernatant solution was collected. The precipitate was washed twice with 50% acetonitrile/H_2O, centrifuged and the supernatant solutions were combined followed by purification using 0-60% B in 30 min gradient to isolate the SUMO(49-93)-Dbz-6(Arg) **10** in 10% yield (3 mg).

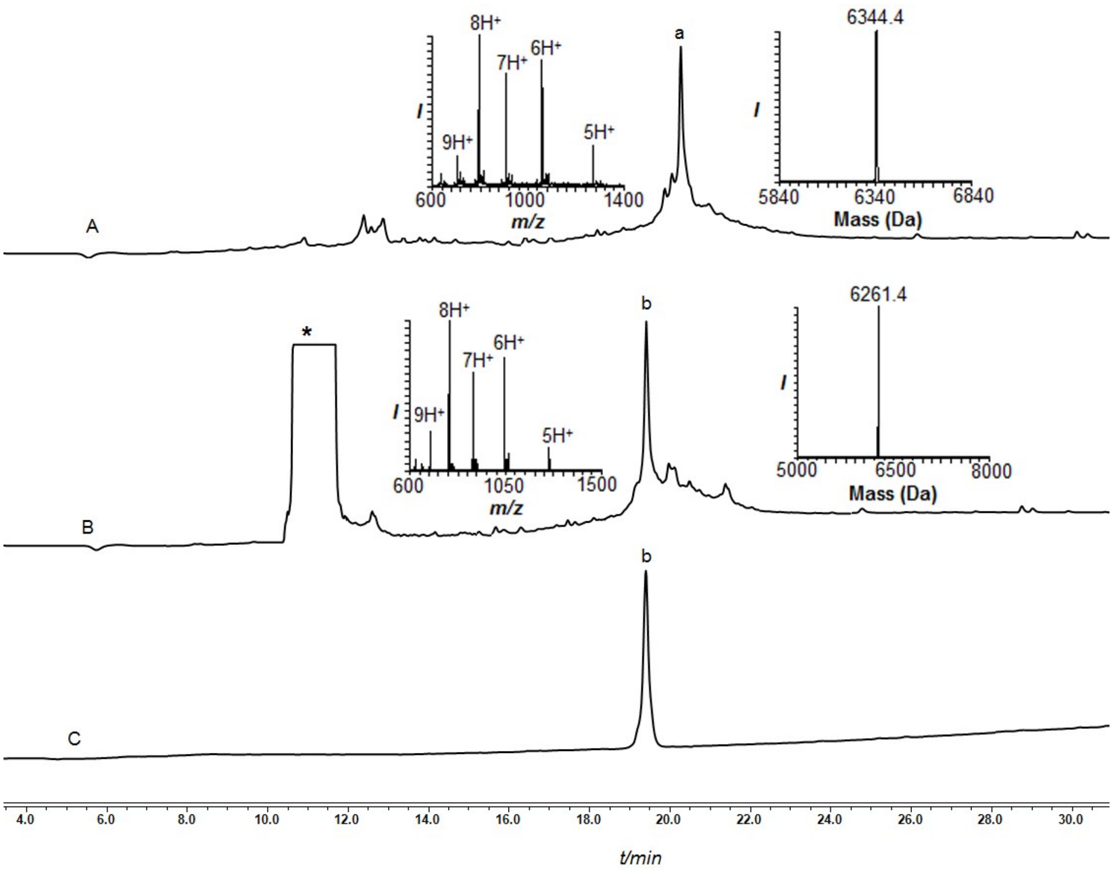

Figure S16: Synthesis of SUMO(49-93)-Dbz-$(Arg)_6$ by using proc protection on Dbz; A) Crude HPLC and mass analysis of SUMO(49-93)-Dbz(proc)-$(Arg)_6$: peak a corresponds to SUMO(49-93)-Dbz(proc)-$(Arg)_6$ with the observed mass of 6344.4 ± 0.6 Da, calcd 6345.7. B) analytical HPLC and mass traces of SUMO(49-93)-Dbz-$(Arg)_6$ after Proc removal: peak b corresponds to the Proc removed SUMO(49-93)-Dbz-$(Arg)_6$ with the observed mass of 6261.1 ± 0.7 Da, calcd 6262.7 Da. C) HPLC analysis of purified SUMO(49-93)-Dbz(proc)-$(Arg)_6$.

Cleavage of Dbz-$(Arg)_6$ linker on model peptide SUMO(49-93):

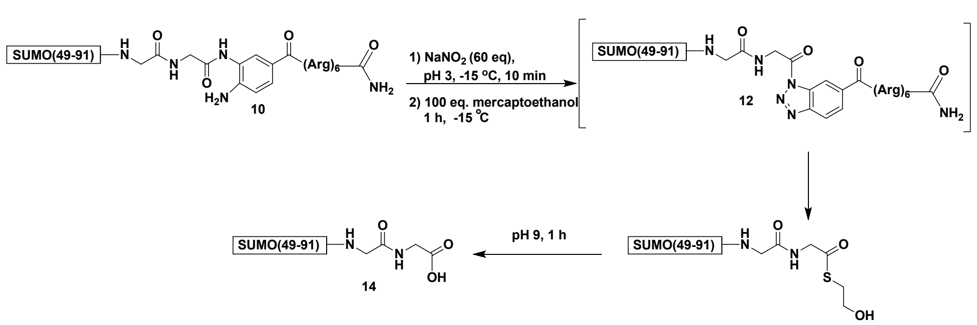

SUMO(49-93)-Dbz-Arg_6 (2 mg, 3.1×10^{-4} mmol) was dissolved in 6 M Gn•HCl, 200 mM phosphate (160 µL, 2 mM) containing 120 mM $NaNO_2$ at pH 3, -15 °C . After 10 min mercapto ethanol (2.5 µL 0.031 mmol) was added and the reaction was continued for additional 1 h. Following this, the reaction mixture was adjusted to pH 9 and kept at room temperature for 1 h. The progress of the reaction was monitored using analytical HPLC using C18 column with a gradient 0-60% B over 30 min.

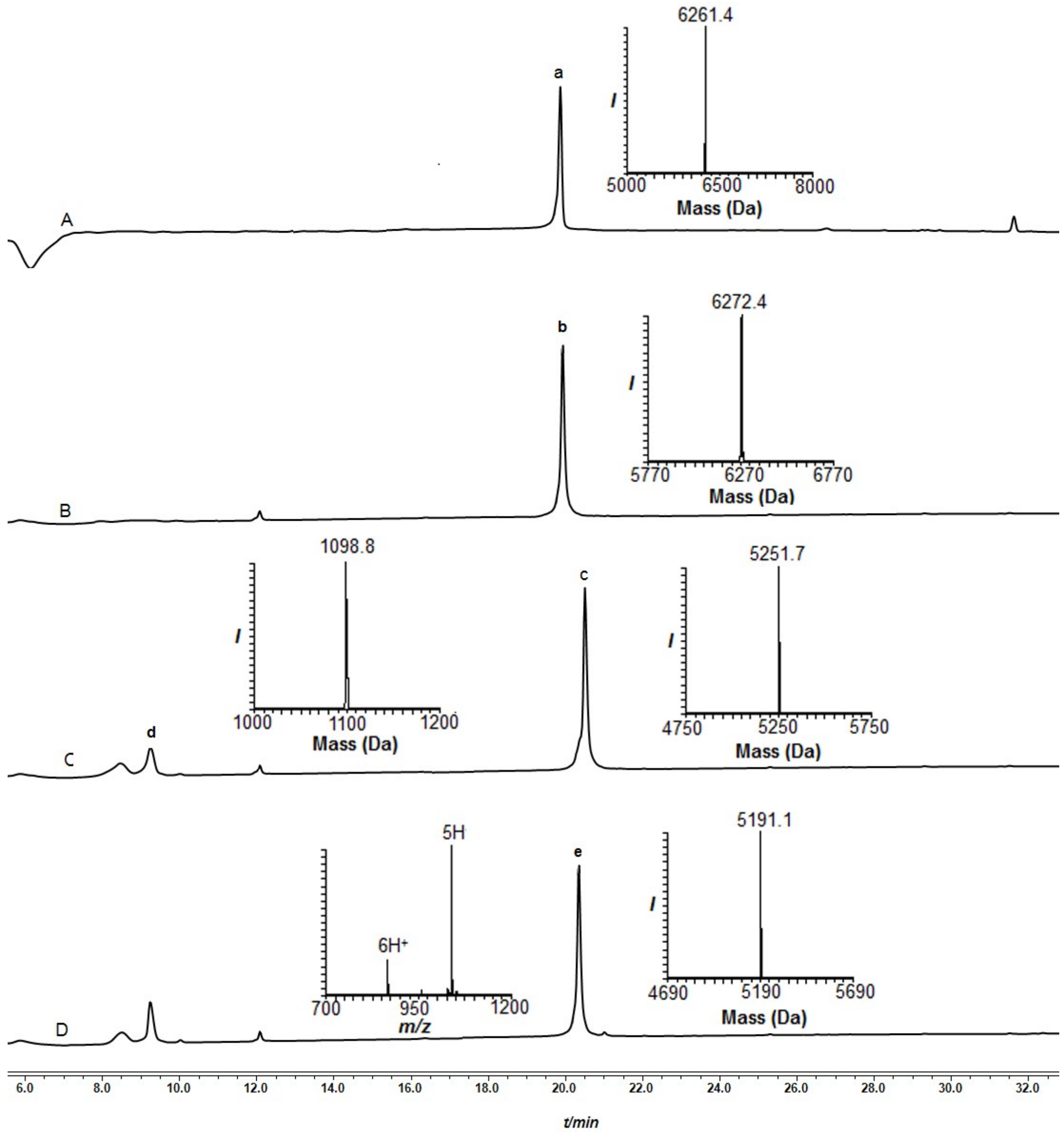

Figure S17: Hydrolysis of SUMO(49-93)-Dbz-$(Arg)_6$; A) Peak a corresponds to SUMO(49-93)-Dbz-$(Arg)_6$ **10** with the observed masses of 6261.4 ± 0.6 Da, calcd 6262.7

Da. B) After 10 min of $NaNO_2$ treatment: peak b corresponding SUMO(49-93)-benzotriazole **12** with observed masses of 6272.4 ± 0.5 Da calcd 6273.7 Da. C) after 1 hour of the mercaptoethanol addition: peak c corresponds to SUMO(47-93)-CO-SCH_2CH_2OH with the observed mass of 5251.5 ± 0.6 Da, calcd 5252.5 Da, peak d corresponds to the benzotriazole containing the Arg tag. D) After 1 h of SUMO(47-93)-CO-SCH_2CH_2OH at pH 9: peak e corresponds to the SUMO(47-93)-COOH **14** with the observed mass 5191.1 ± 0.9 Da, calcd 5192.6 Da.

Synthesis of Cys-SUMO(47-93)-Dbz-$(Arg)_6$ 15:

The procedure described for SUMO(49-93)-Dbz-$(Arg)_6$ using Proc protection was followed. The analytical HPLC was carried using C4 column with a gradient of 0-60% B over 30 min. The same gradient was used to isolate the pure compound in preparative HPLC.

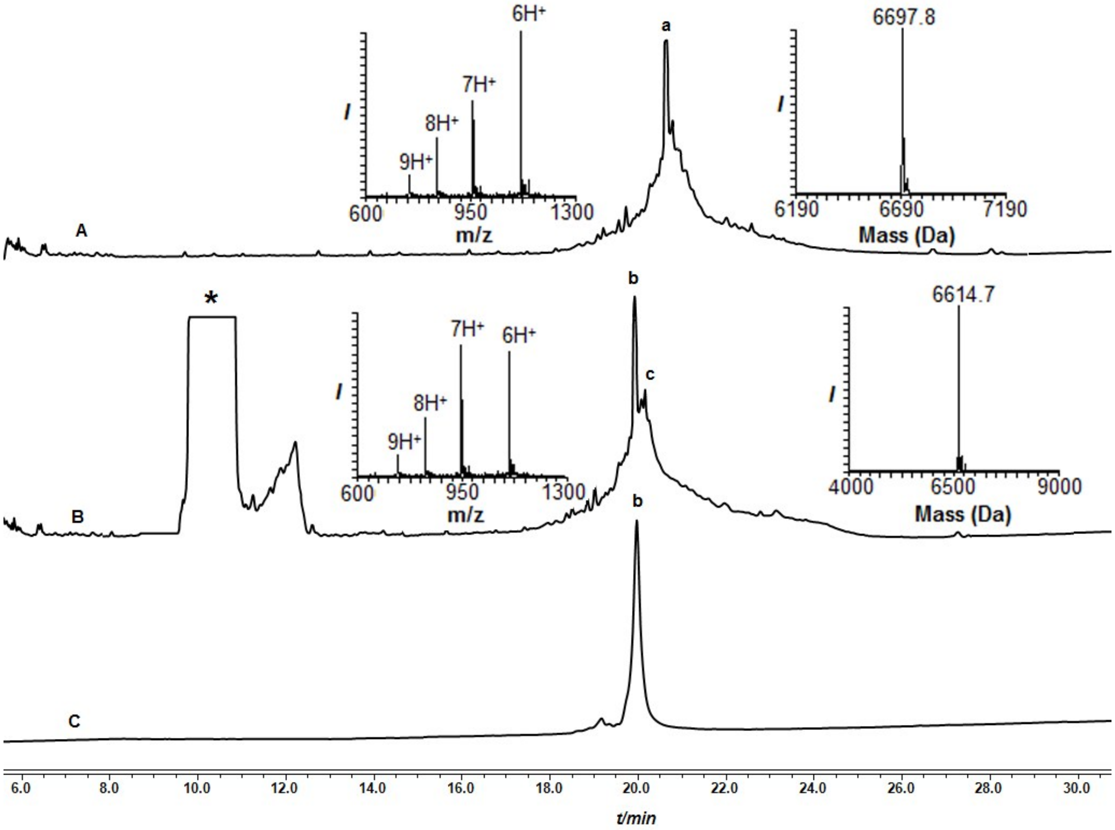

Figure S18: Synthesis of Cys-SUMO(47-93)-Dbz-$(Arg)_6$ **15** by using proc protection on Dbz; A) Crude HPLC and mass analysis of Cys-SUMO(47-93)-Dbz-$(Arg)_6$: peak a corresponds to Cys-SUMO(49-93)-Dbz(proc)-$(Arg)_6$ with the observed mass of 6697.8 ± 1.2 Da, calcd 6699.1 Da. B) analytical HPLC and mass traces of Cys-SUMO(47-93)-Dbz-$(Arg)_6$ after proc removal: peak b corresponds to the Cys-SUMO(46-93)-Dbz-$(Arg)_6$ **15** with the observed mass of 6614.7 ± 1.3 Da, calcd 6617.1 Da, peak c has an

unidentified mass. C) HPLC analysis of the purified Cys-SUMO(47-93)-Dbz(proc)-$(Arg)_6$ **15**.

Synthesis of Cys-SUMO(2-93)-Dbz-Arg_6 :

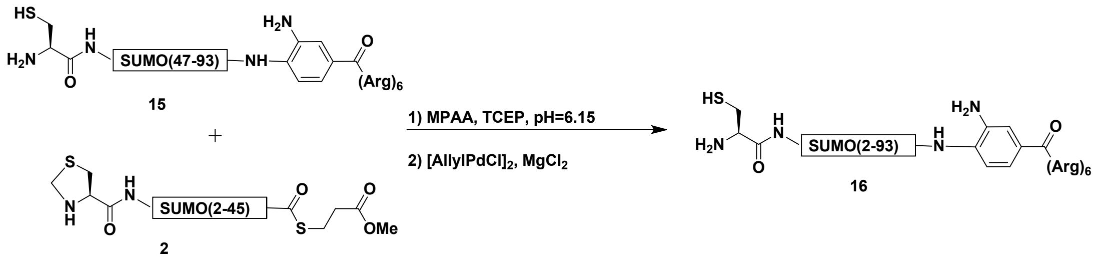

Thz-SUMO(2-45)-MMP (8.5 mg, 1.6×10^{-3} mmol) and Cys-SUMO(47-93)-Dbz-Arg_6 (10 mg, 1.5×10^{-3} mmol) were dissolved in 6 M Gn•HCl containing 200 mM phosphate and of MPAA (25.4 mg, 0.15 mmol) and TCEP (21.6 mg, 0.07 mmol) at pH 6.1. The reaction was kept at 37 °C for 5 h. After completion of the ligation, magnesium chloride (28.7 mg, 0.30 mmol) was added and the reaction mixture was incubated at 37 °C for 10 min. Subsequently, allylpalladium chloride dimer ($[Pd(allyl)Cl]_2$) (16.5 mg, 0.045 mmol) was added and the reaction mixture was kept at 37 °C for 2 h . The progress of the reaction was monitored by analytical HPLC using C4 column with the gradient 0-60%B over 30 min. The product was purified using a C4 semi-preparative column and with the same gradient to obtain Cys-SUMO(2-93)-Dbz-Arg_6 **16** in 35 % yield (6 mg).

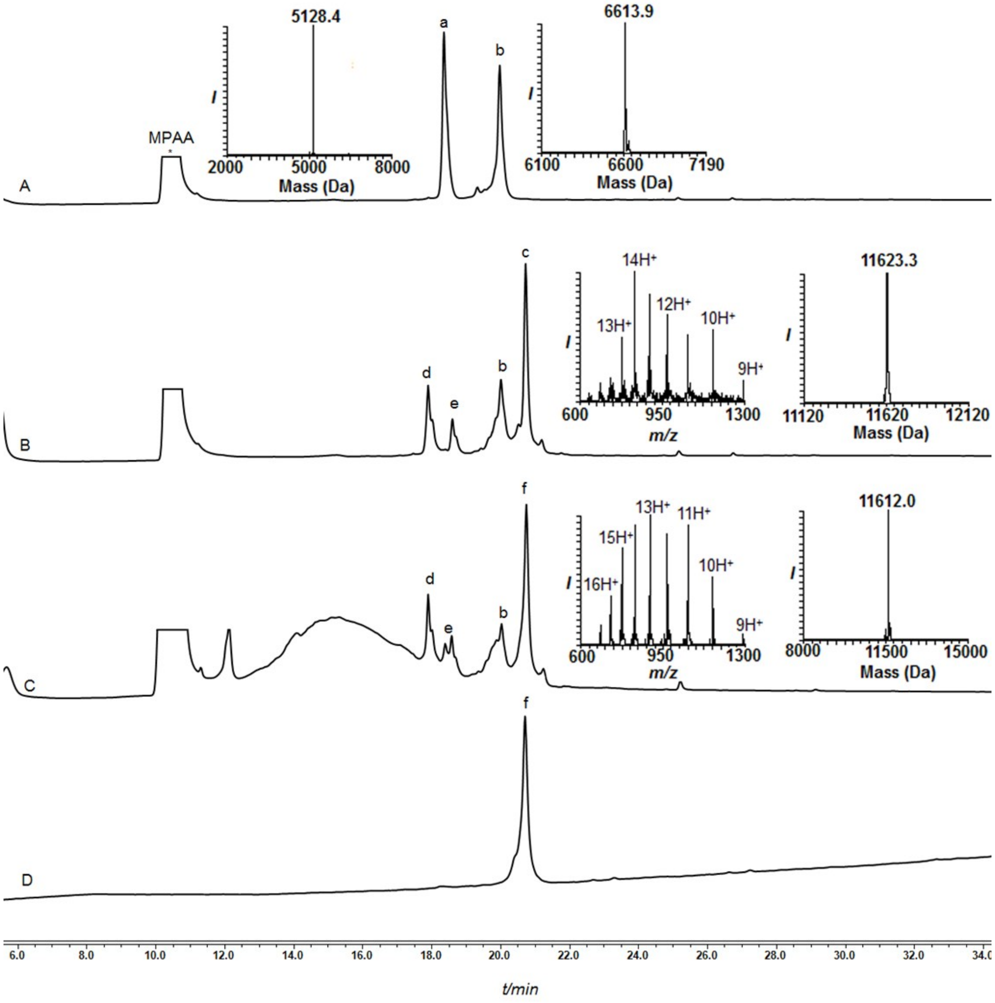

Figure S19: Synthesis of Cys-SUMO(2-93)-Dbz-$(Arg)_6$; A) Analytical HPLC and mass traces of the ligation between Thz-SUMO(2-45)-MMP and Cys-SUMO(47-93)-Dbz-$(Arg)_{6,}$ peak a corresponds to Thz-SUMO(2-45)-MMP with the observed mass of 5128.4 ± 0.1 Da, calcd 5128.7 Da and peak b corresponds to Cys-SUMO(47-93)-Dbz-$(Arg)_6$ with the observed mass of 6613.9 ± 1.9 Da, calcd 6617.1 Da, at time zero. B) Ligation after 5 h: peak c corresponds to the desired ligation product Thz-SUMO(2-93)-Dbz-$(Arg)_6$ with the observed mass of 11,623.3 ± 1.1 Da, calcd 11625.8 Da, peak d corresponds to the Thz-SUMO(2-45)-COOH and peak e corresponds to Thz-SUMO(2-45)-CO-lactam. C) Thz-opening by $[Pd(allyl)Cl]_2$ after 2 h: peak f corresponds to thz-opened Cys-SUMO(2-93)-Dbz-$(Arg)_6$ with the observed mass of 11612.0 ± 0.8 Da, calcd 11613.8 Da. D) Analytical HPLC of purified Cys-SUMO(2-93)-Dbz-$(Arg)_6$.

Synthesis of Ub(K63*)-Cys-SUMO(2-93) -Dbz-Arg$_6$: see comment 23

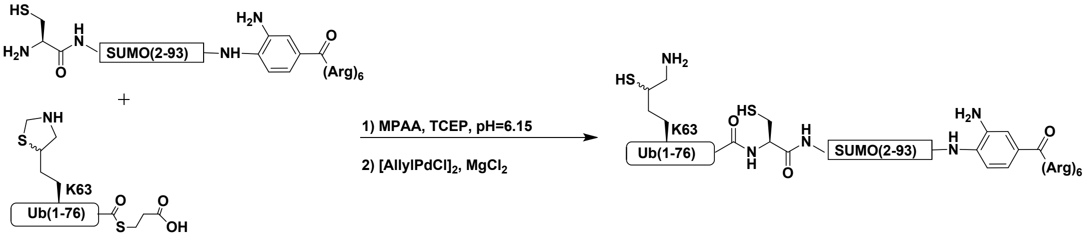

Ub(K63*)-MPA (5.7 mg, 6.6×10^{-4} mmol) and Cys-SUMO(2-93)-Dbz-Arg$_6$ (7 mg, 6.02×10^{-4} mmol) were dissolved in 6 M Gn•HCl (600 μL, 1 mM) containing 200 mM phosphate and MPAA (10 mg, 0.060 mmol) and TCEP (8 mg, 0.030 mmol) at pH 6.1. The reaction was kept at 37 °C for 5 h. After completion of ligation, magnesium chloride (11.4 mg, 0.120 mmol) was added and the reaction mixture was incubated at 37°C, for 10 min. Subsequently, allylpalladium chloride dimer was added ([Pd(allyl)Cl]$_2$) (6.6 mg, 0.018mmol) and the reaction mixture was kept at 37 °C for 2 h. The progress of the reaction was monitored by analytical HPLC using C4 column with a gradient 0-60% B over 30 min. The resultant product Ub(K63*)-Cys-SUMO(2-93) -Dbz-Arg$_6$ was purified using a C4 semi preparative column and with the same gradient to obtain the desired product **17** in 30 % yield (4.2 mg).

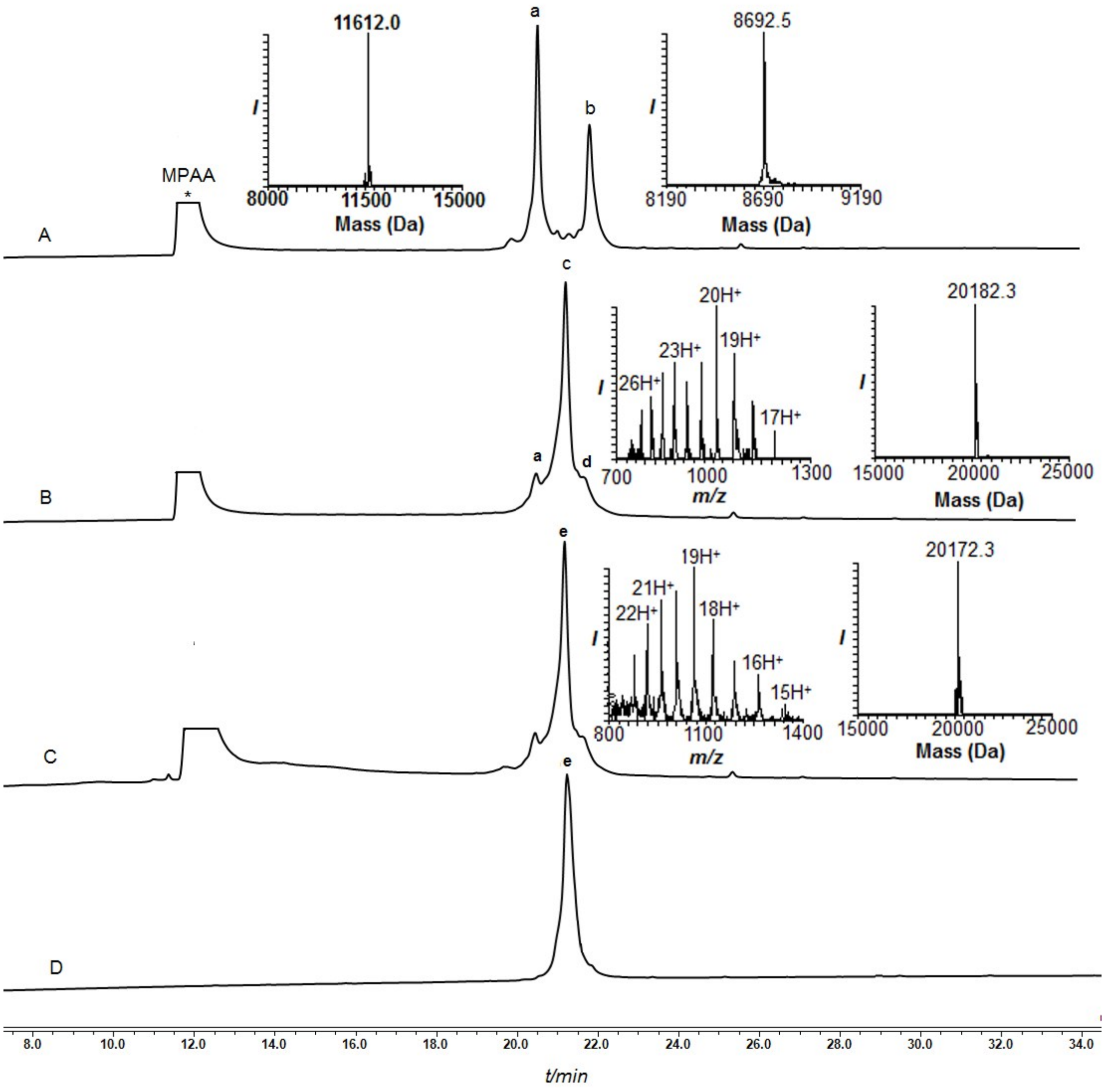

Figure S20: Synthesis of Ub(K63*)-Cys-SUMO(2-93)-Dbz-$(Arg)_6$; A) Analytical HPLC and mass traces of the ligation between Cys-SUMO(2-93)-Dbz-$(Arg)_6$ and Ub(K63*)-MMP, peak a corresponds to Cys-SUMO(2-93)-Dbz-$(Arg)_6$ with the observed mass of 11612.0 ± 0.8 Da, calcd 11613.8 Da, peak b corresponds to Ub(K63*)-MMP with the observed mass of 8692.5 ± 0.4 Da, calcd 8691.6 Da at time zero. B) Ligation after 5 h, peak c corresponds to the desired ligation product Ub(K63*)-Cys-SUMO(2-93)-Dbz-$(Arg)_6$ with the observed mass of 20182.3 ± 2.1 Da, calcd 20186.7 Da, peak d corresponds to the Ub(K63*)-COOH. C) Thz-opening by $([Pd(allyl)Cl]_2$ after 2 h, peak e corresponds to thz-opened Ub(K63*)-Cys-SUMO(2-93)-Dbz-$(Arg)_6$ with the observed mass of 20,172.3 ± 1.9 Da, calcd 20174.7 Da. D) Analytical HPLC of purified Ub(K63*)-Cys-SUMO(2-93)-Dbz-$(Arg)_6$.

Synthesis of di-Ub(K63*)-Ala-SUMO(2-93)-Dbz-Arg$_6$ 8:

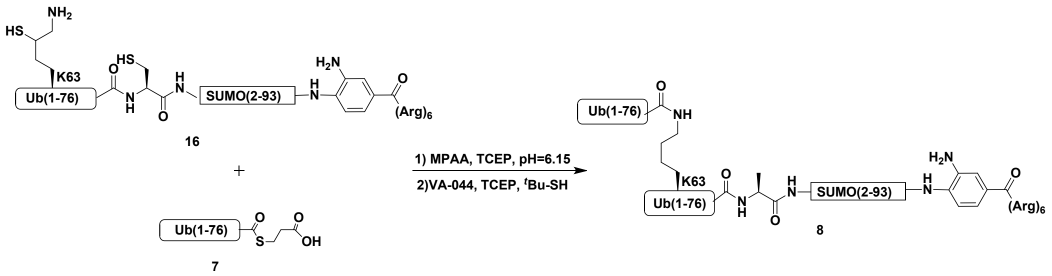

Ub-MPA **7** (2.3 mg, 2.7×10^{-4} mmol) and Ub(K63*)-Cys-SUMO(2-93)-Dbz-Arg$_6$ **16** (5 mg, 2.47×10^{-4} mmol) were dissolved in 6 M Gn•HCl (172 μL, 1mM) containing 200 mM phosphate and MPAA (4.1 mg, 0.024 mmol) and TCEP (3.4 mg, 0.012 mmol) at pH 6.1. The reaction was kept at 37 ^{o}C for 8 h. After completion, the reaction mixture was dialysed in a Slide-A-Lyzer 3.5K dialysis cassette (Thermo scientific, 0.1-0.5 mL) in 6 M Gn•HCl, 200 mM phosphate buffer (500 mL) for overnight and then subjected to radical induced desulfurization by adding TCEP (25 mg, 0.087 mmol), VA-044 (9.6 mg, 0.029 mmol) and *tert*-butyl thiol (30 μL) and incubated at 37 °C for 10 h. Progress of the reaction was monitored by analytical HPLC C4 column with a gradient of 0-60% B over 30 min. For semi-preparative HPLC, the same gradient was used to isolate the product di-Ub(K63*)-Ala-SUMO(2-93)-Dbz-Arg$_6$ **8** in ~30% yield (2.1 mg).

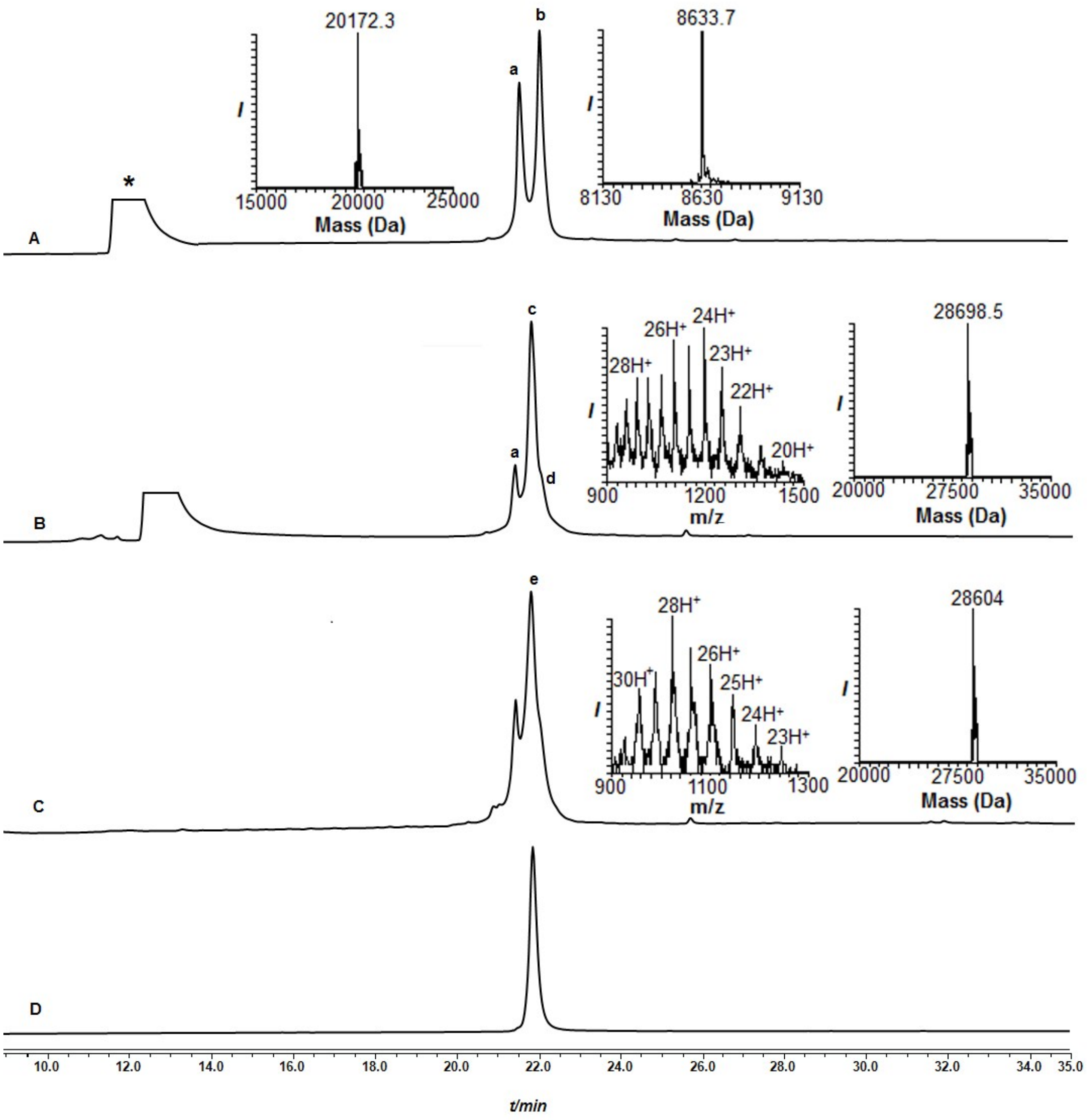

Figure S21: Synthesis of di-Ub(K63)-Ala-SUMO(2-93)-Dbz-(Arg)$_6$; A) Analytical HPLC and mass traces of the ligtion between and Ub-MPA, peak a corresponds to Ub(K63*)-Cys-SUMO(2-93)-Dbz-(Arg)$_6$ with the observed mass of 20,172.3 ± 1.2 Da, calcd 20,174.7 Da, peak b corresponds to Ub-MPA with the observed mass of 8,633.7 ± 0.1 Da, calcd 8,633.8 Da at time zero. B) Ligation after 8 h, peak c corresponds to the desired ligation product di-Ub(K63*)-Cys-SUMO(2-93)-Dbz-(Arg)$_6$ with the observed mass of 28,698.5 ± 2.3 Da, calcd 28,702.5 Da, peak d corresponds to the Ub-COOH. C) Desulfurization reaction after 10 h: peak e corresponds to desulfurized di-Ub(K63*)-Ala-SUMO(2-93)-Dbz-(Arg)$_6$ with the observed mass of 28604 ± 2.1 Da, calcd 28607.3 Da. D) HPLC analysis of purified di-Ub(K63)-Ala-SUMO(2-93)-Dbz-(Arg)$_6$.

Cleavage of Dbz-(Arg)$_6$ tag from di-Ub(K63)-Ala-SUMO(2-93)-Dbz-Arg$_6$ 8:

The obtained di-Ub(K63)-Ala-SUMO(2-93)-Dbz-Arg$_6$ (2.1mg) was treated with $NaNO_2$/mercaptoethanol at pH 3, as we have optimized for the model peptide and the progress of the reaction was monitored by analytical HPLC using C4 column with a gradient of 0-60% B and the same gradient was used to isolate the compound in 61% yield (1.3 mg).

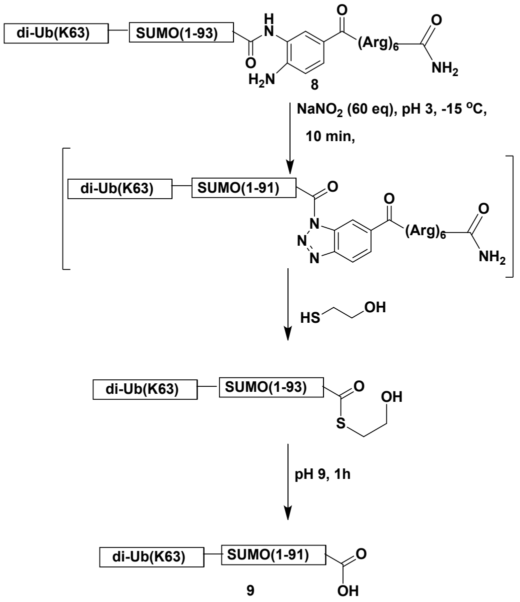

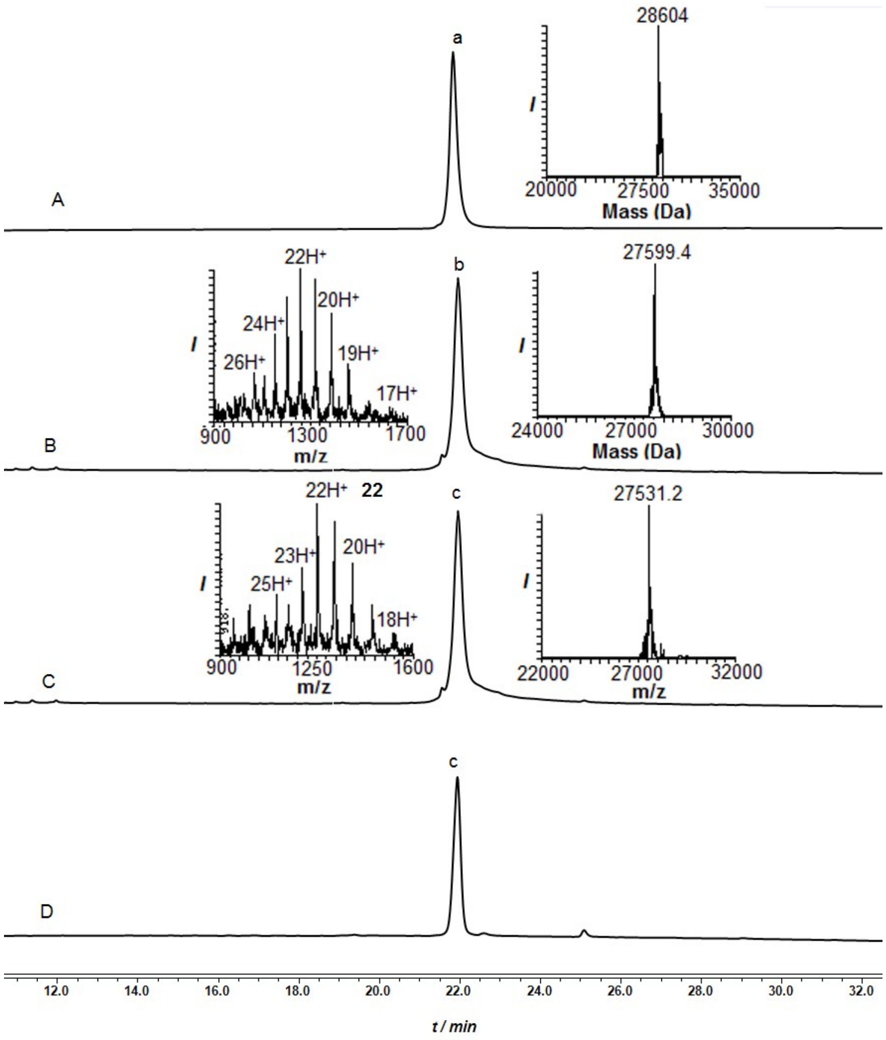

Figure S22: Hydrolysis of di-Ub(K63)-Ala-SUMO(2-93)-Dbz-$(Arg)_6$ **8**; A) Peak a corresponding to di-Ub(K63*)SUMO(2-93)-Dbz-$(Arg)_6$ with observed masses of 28604.0 ± 1.7 Da, calcd 28607.3 Da. B) After 10 min $NaNO_2$ treatment followed by 1 h treatment with mercaptoethanol at -15 ^{o}C (pH =3): peak b corresponds to di-Ub(K63)-Ala-SUMO(49-93)-SCH_2CH_2OH with the observed masses of 27599.4 Da, calcd 27601.5 ± 1.1 Da. C) Reaction at pH 9 after 1 h: peak c corresponds to di-Ub(K63)-Ala-SUMO(2-93)-COOH **9** with the observed mass of 27531.1 ± 2.3 Da, calcd 27535.2 Da.

Scheme for the overall synthesis of of SUMO-2-Lys63-linked diubiquitin hybrid chains using two (phenylactamidomethyl)-Arg$_3$ solubilizing tags:

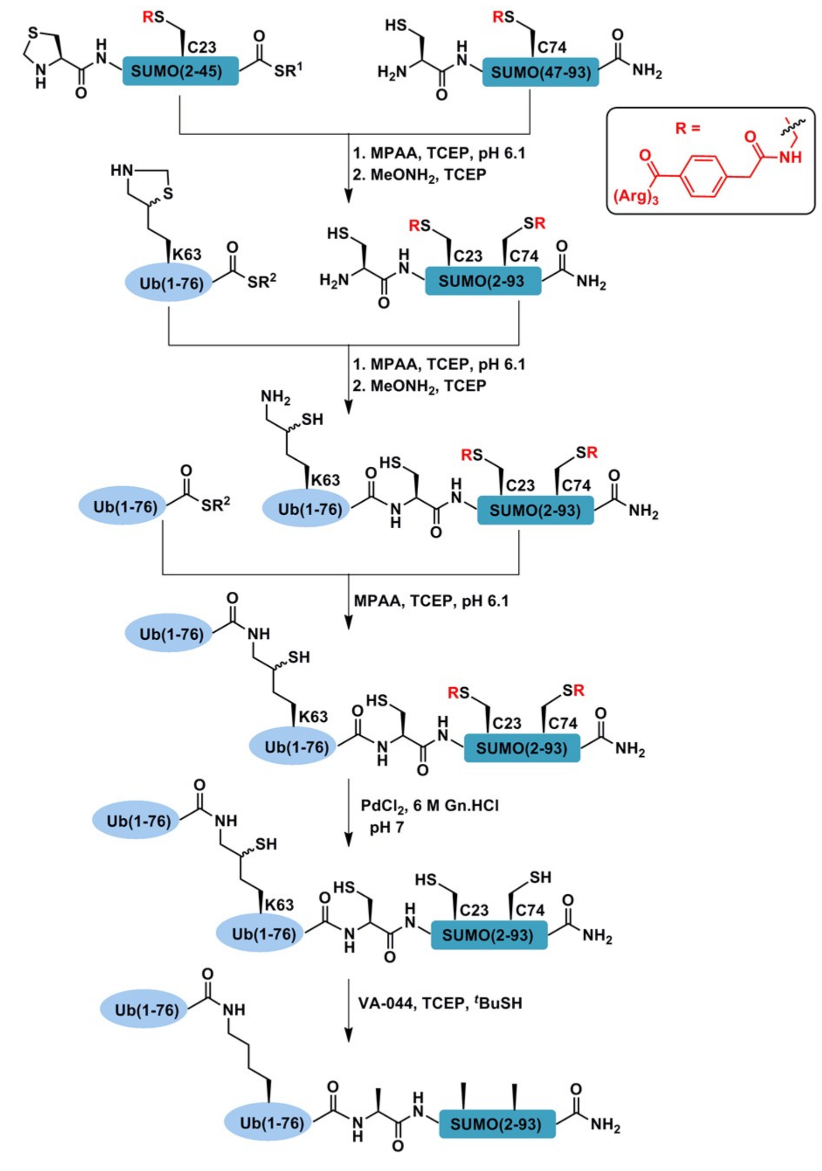

Synthesis of Cys-SUMO-(47-93, A74C*) (C* is cysteine bearing (phenylactamidomethyl)-Arg$_3$) 19:

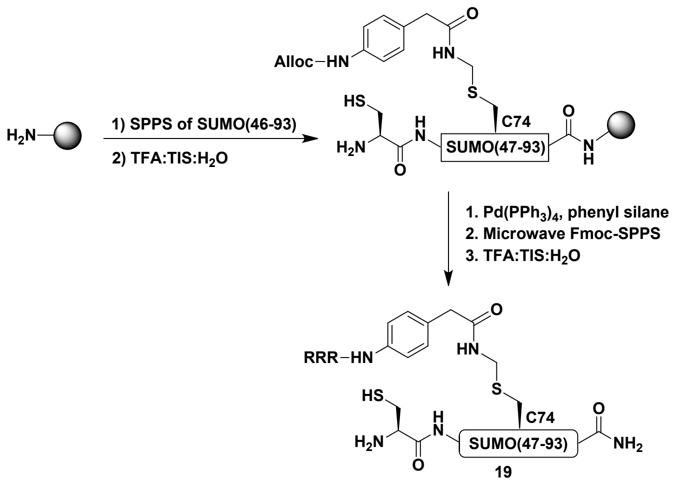

In the synthesis of Cys-SUMO-(47-93, A74C*) the procedure described above for Cys-SUMO-(47-93) was followed. In addition, the phacm linker (Cys-phenylacetamidomethyl-alloc) was coupled manually at Ala74 using 2.5 equiv of HATU and 5 equiv of DIEA for 2 h. Analytical cleavage was performed using regular cleavage cocktail to ensure complete coupling. Samples were analyzed using analytical HPLC C4 column, with a gradient of 0-60% B over 30 min.

Alloc-removal on Phacm linker: For 0.1 mmol scale, Alloc deprotection on Phacm was carried out by treating with mixture of $Pd(PPh_3)_4$ (24 mg, 0.020 mmol) and phenylsilane (240 mL, mmol) in 3 mL of dry DCM for 1 h.

Arg coupling on Phacm: All the three Arg were coupled on microwave as described in the **SUMO(49-93)-Dbz-(Arg)$_6$**:

Finally, the peptide was cleaved from the resin with the cleavage cocktail as mentioned above for Thz-SUMO(2-45)-Nbz and purified using preparative HPLC C18 column with a gradient of 0-60%B over 30 min.

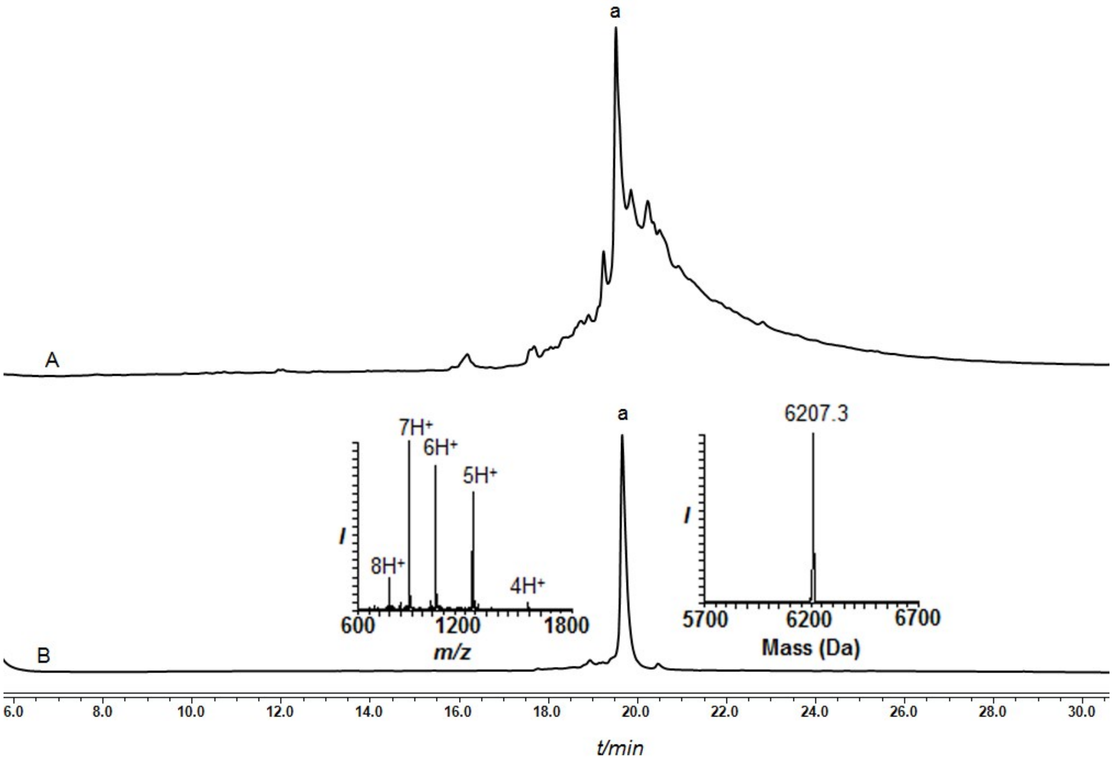

Figure S23: Synthesis of Cys-SUMO-(47-93, A74C*): A) Analytical HPLC of crude Cys-SUMO-(47-93, A74C*). B) Analytical HPLC and mass analysis of purified Cys-SUMO-(47-93, A74C*) with the observed mass of 6207.3 ± 1.1 Da, calcd 6209.8 Da.

Synthesis of Thz-SUMO-(2-45, A23C*)-MMP 18:

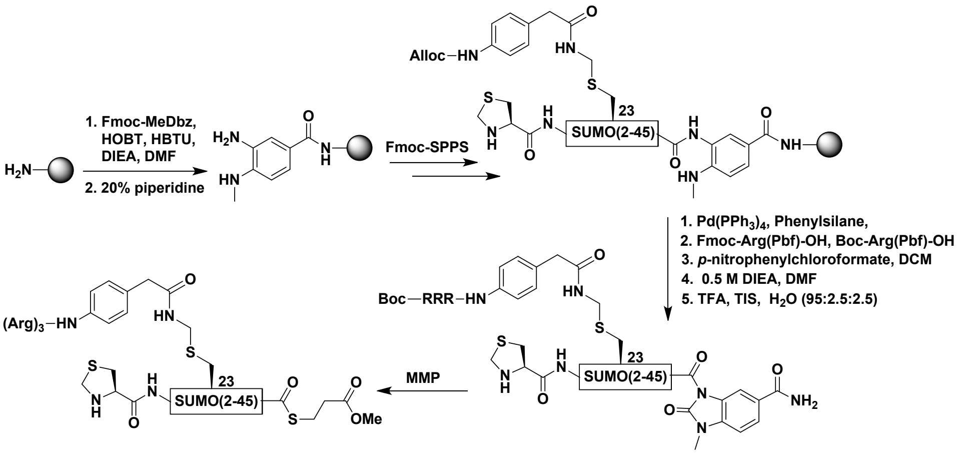

The procedure for Thz-SUMO-(2-45) was followed. In addition, the phacm linker was coupled manually at position 23 using 2.5 equiv of HATU and 5 equiv of DIEA for 2 h. The N-terminal Met was mutated to Thz.

MeDbz cyclization: The procedure used for Thz-SUMO(2-45)-MMP fragment was followed.

Clevage of the peptide from resin: The procedure used for Thz-SUMO(2-45)-Nbz was followed.

Switching of Nbz to MMP thioeser: The procedure used for Thz-SUMO(2-45)-MMP fragment was followed. The product was isolated using C4 column with a gradient of 5-55% over 45 min.

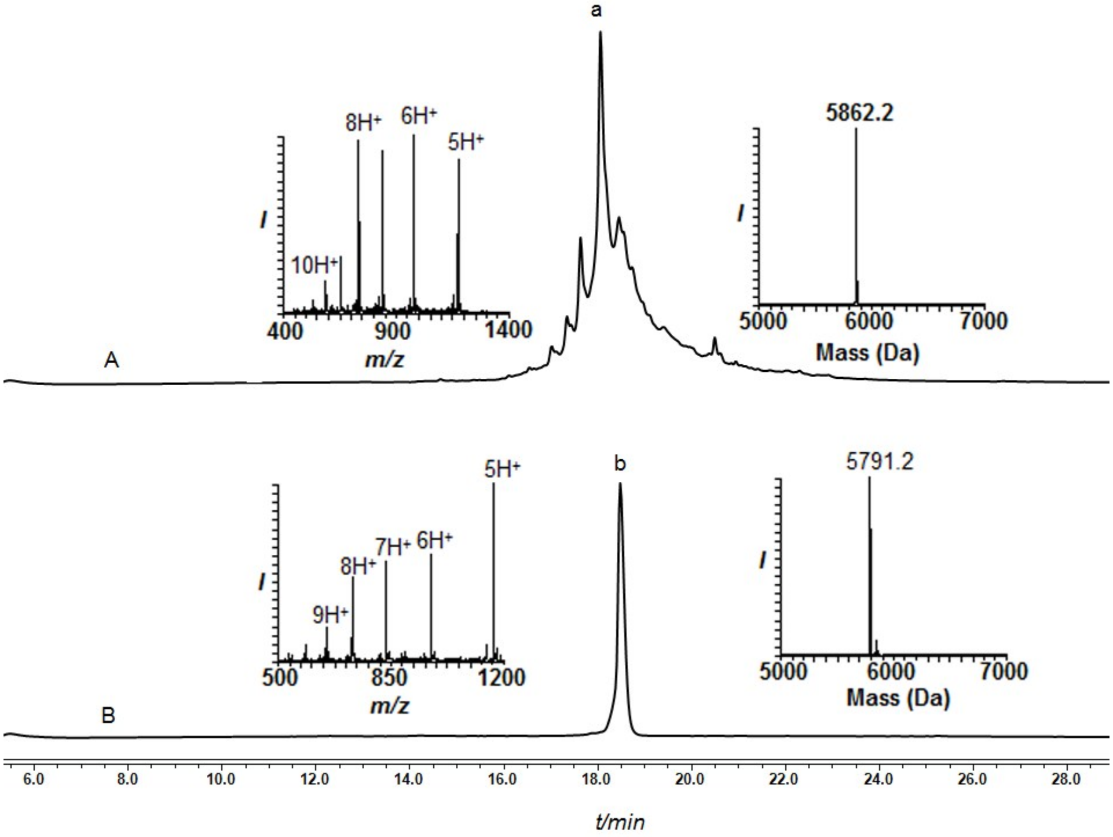

Figure S24: Synthesis of Thz-SUMO-(2-45, A23C*)-MMP A) Analytical HPLC of crude Thz-SUMO-(2-45, A23C*)-MeNbz with the observed mass of 5,862.2 ± 0.3 Da, calcd 5,862.8 Da. B) Analytical HPLC and mass analysis of purified Thz-SUMO-(2-45, A23C*)-MMP with the observed mass of 5791.2 ± 0.3 Da; calcd 5791.8 Da.

Synthesis of Cys-SUMO(2-93) bearing two Phacm tags 20:

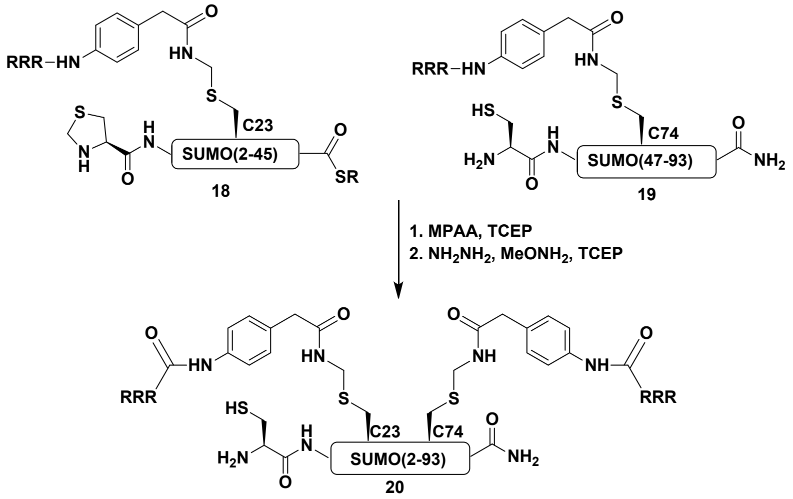

Thz-SUMO-(2-45, A23C*)-SR **18** (9.3 mg, 1.61×10^{-3} mmol) and Cys-SUMO(46-93, A74C*) **19** (10 mg, 1.61×10^{-3} mmol) were dissolved in 6 M Gn•HCl, 200 mM phosphate buffer (900 µL, 2 mM) containing MPAA (30 mg, 0.18 mmol) and TCEP(25 mg 0.09 mmol) at pH 6.2. The reaction was kept at 37 °C for 5 h. After completion of ligation, hydrazine solution (100 µL of 10% v/v hydrazine in Gn•HCl) at pH 9 was added and the reaction was kept at room temperature for 1 h. Subsequently, $MeONH_2$ (5 mg, 0.063 mmol) and TCEP (10.3 mg, 0.03 mmol) were added to the ligation mixture and the reaction was incubated at 37 °C for 5 h. The progress of the reaction was monitored using analytical HPLC and C4 Column with a gradient 5-55% B over 40 min. The resultant product was purified using a C4 semi-preparative column and with the same gradient of solvent system to obtain Cys-SUMO(2-93, A23C*, A74C*) **20** in 51 % yield (9.8 mg).

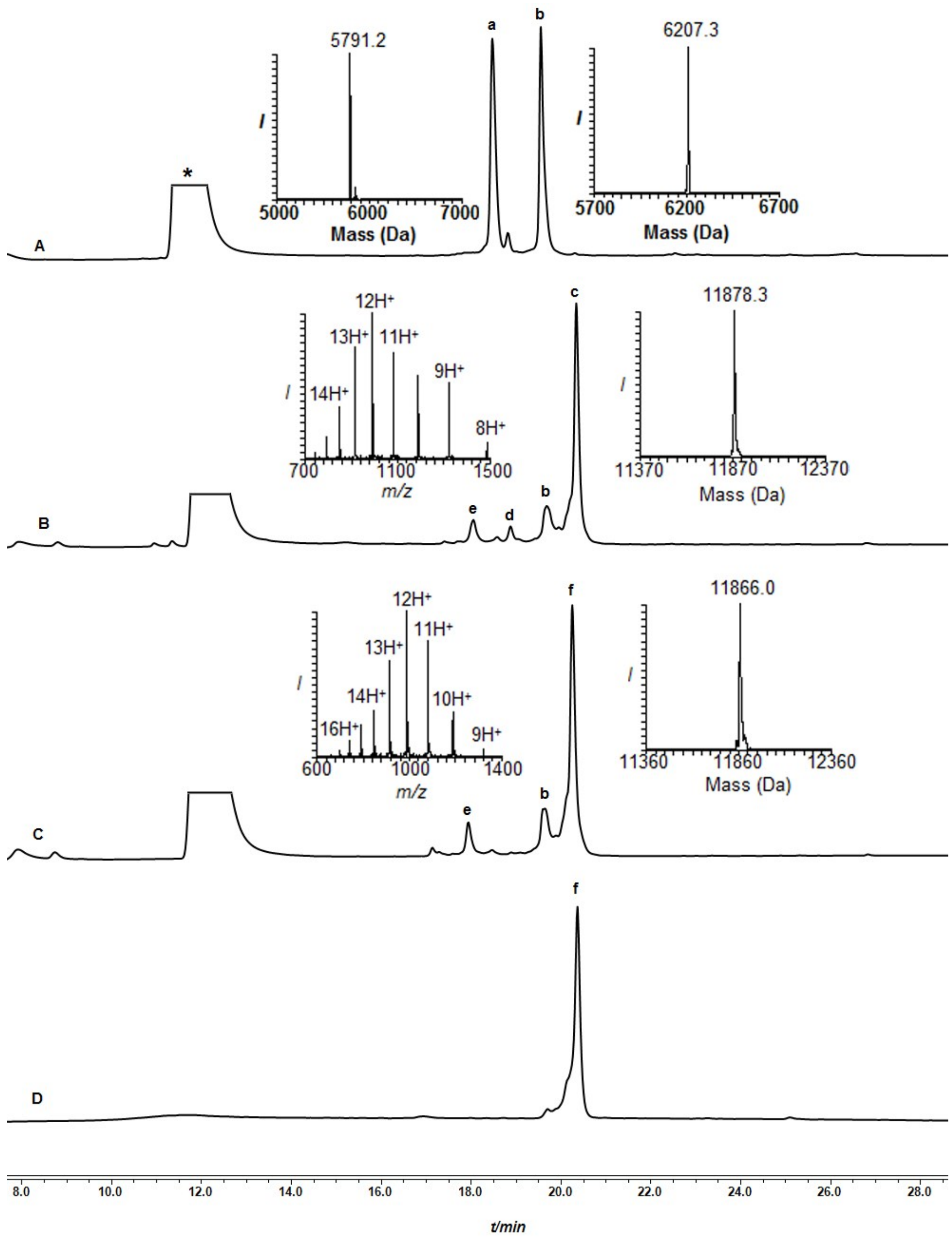

Figure S25: Synthesis of Cys-SUMO-(2-93, A23C*, A74C*). A) Analytical HPLC and mass traces of the ligation between Thz-SUMO(2-45 A23C*)-MMP and Cys-SUMO(47-93 A74C*), peak a corresponds to Thz-SUMO(2-45 A23C*)-MMP with the observed mass of 5791.2 ± 0.3 Da, calcd 5791.8 Da, peak b corresponds to Cys-SUMO(47-93 A74C*) with the observed mass of 6207.3 ± 1.1 Da, calcd 6209.8 Da at time zero; B) Ligation after 5 h, peak c corresponds to desired ligation product Thz-SUMO(2-93 A23C*, A74C*) with the observed mass of 11,878.3 ± 0.8 Da, calcd 11879.4 Da, peak d corresponds to the Thz-SUMO(2-45)-MPAA and peak e corresponds to Thz-SUMO(2-

45)-COOH. C) Thz-opening by NH_2NH_2 & $MeONH_2$ after 6 h, peak f corresponds to thz-opened Cys-SUMO-(2-93, A23C*, A74C*) with the observed mass of 11866.0 ± 1.3 Da, calcd 11867.4 Da. D) Analytical HPLC of purified Cys-SUMO-(2-93, A23C*, A74C*).

Synthesis of Ub(K63*)-Cys-SUMO(47-93) bearing two Phacm tags 21:

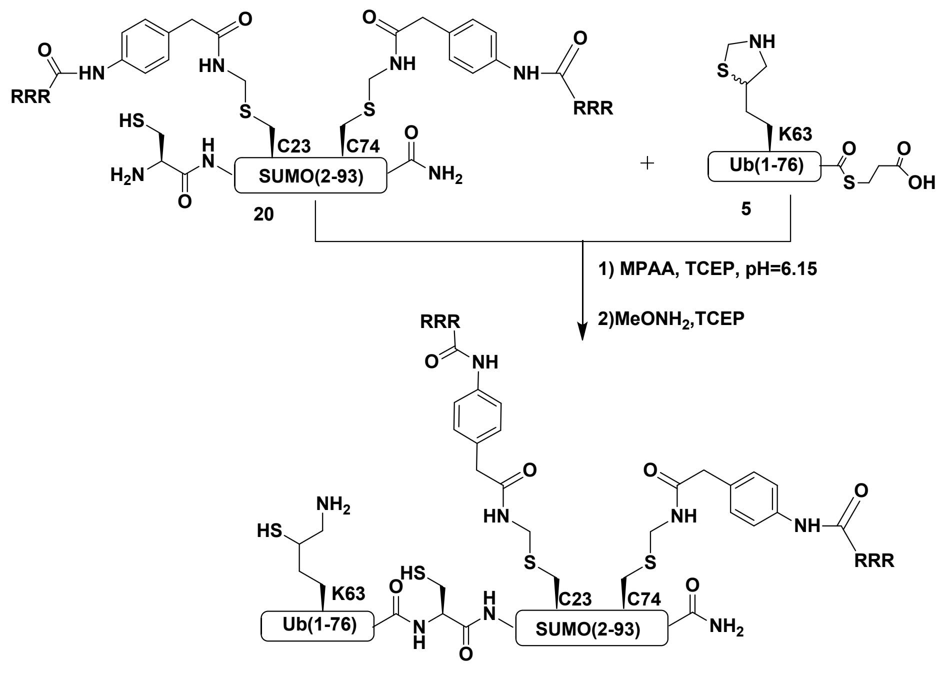

Ub(K63*)-MPA **5** (7.1 mg, 8.25×10^{-4} mmol) and Cys-SUMO(2-93 A23C*, A74C*) **20** (9.8 mg, 8.25×10^{-4} mmol) were dissolved in 6 M Gn**.**HCl, 200 mM Phosphate buffer, (0.825 ml, 1 mM) containing MPAA (13.8 mg, 0.082 mmol) and TCEP (11.8 mg, 0.041 mmol) at pH 6.2. The reaction mixture was kept at 37 ^{o}C for 6 h. This was followed by the addition of $MeONH_2$ (2.0 mg, 0.024 mmol) and TCEP (3.5 mg, 0.012 mmol) and incubation of reaction at 42 ^{o}C for 12 h. The progress of the reaction was monitored by analytical HPLC using C4 column with a gradient 0-60% B over 30 min. The same gradient was used to isolate the Ub(K63*)-Cys-SUMO(2-93 A23C*, A74C*) **21** in 53% yield (9.0 mg)**.**

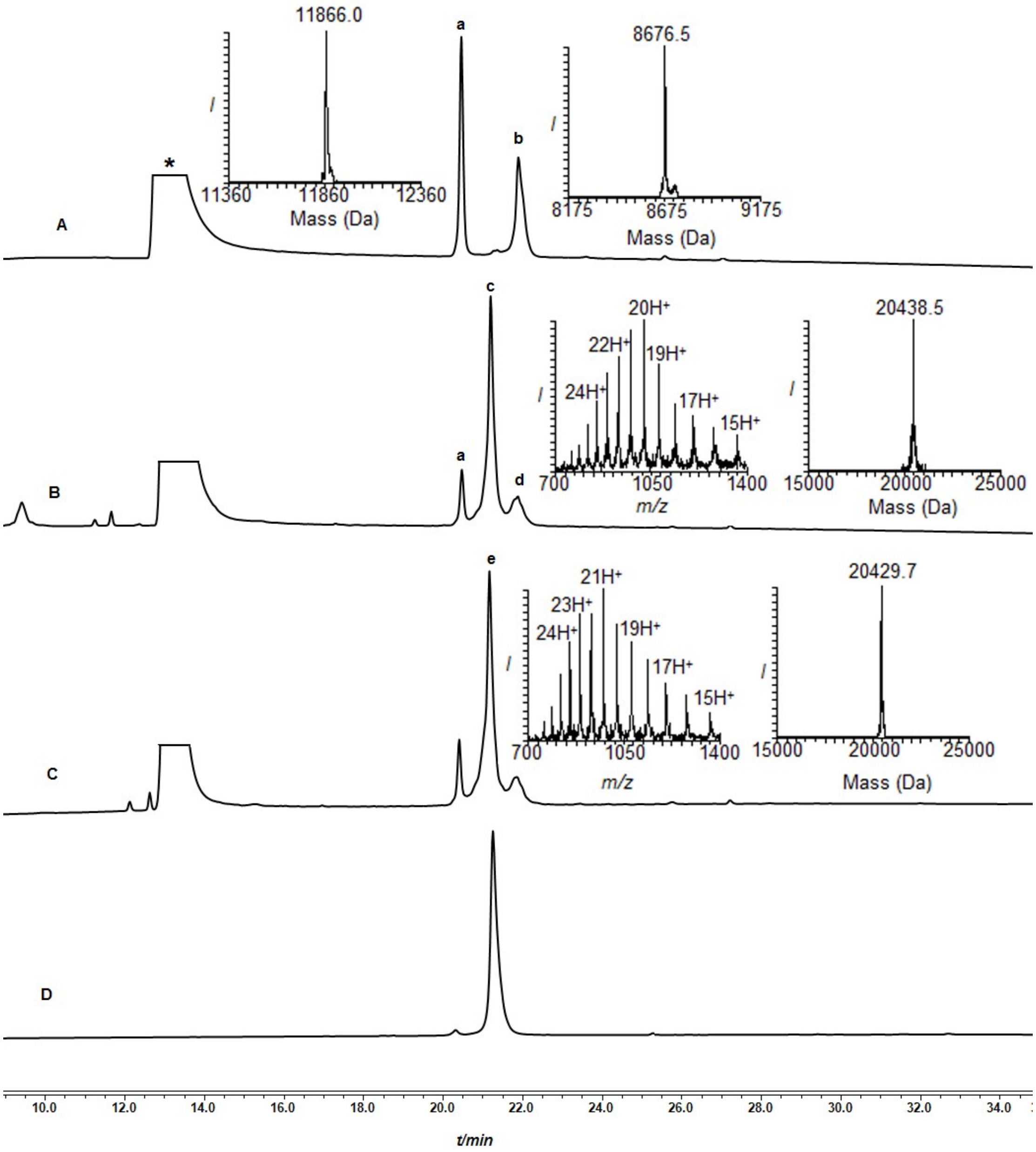

Figure S26: Synthesis of Ub(K63*)-Cys-SUMO-(2-93, A23C*, A74C*). A) Analytical HPLC and mass traces of the ligation between Cys-SUMO-(2-93, A23C*, A74C*) and Ub(K63*)-MPA, peak a corresponds to Cys-SUMO-(2-93, A23C*, A74C*) with the observed mass of 11866.0 ± 1.3 Da, calcd 11867.4 Da, peak b corresponds to Ub(K63*)-MPA with the observed mass of 8676.5 ± 0.2 Da, calcd 8676.8 Da, at time zero. B) Ligation after 5 h: peak c corresponds to desired ligation product Ub(K63* Thz)-Cys-SUMO-(2-93 A23C*, A74C*) with the observed mass of 20.438.5 ± 3.6 Da, calcd 20440.2 Da, peak d corresponds to the Ub(K63*)-COOH. C) Thz-opening by $MeONH_2$ after 12 h: peak e corresponds to thz-opened Ub(K63*)-Cys-SUMO-(2-93, A23C*, A74C*) with the observed mass of 20429.7 ± 3.4 Da, calcd 20428.2 Da. D) Analytical HPLC of purified Ub(K63*)-Cys-SUMO-(2-93 A23C*, A74C*) **21**.

Synthesis of di-Ub(K63)-Cys-SUMO(2-93) bearing two Phacm tags 22.

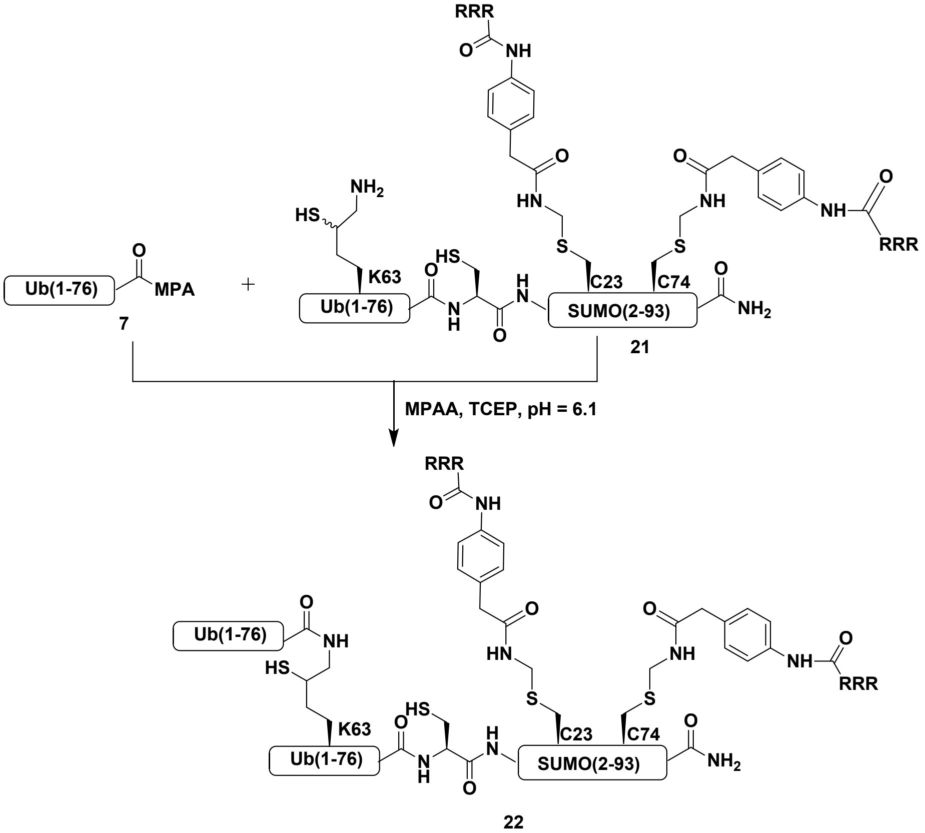

Ub(K63*)-Cys-SUMO(2-93, A23C*, A74C*) **21** (9 mg, 4.4×10^{-4} mmol) and Ub-MPA **7** (3.8 mg, 2.6×10^{-4} mmol) were dissolved in 6 M Gn•HCl, 200 mM phosphate buffer (440 µL, 1mM) containing MPAA (4.3 mg, 0.026 mmol) and TCEP (3.7 mg, 0.013 mmol) at pH 6.1. The reaction was incubated at 37 °C for 7 h. The progress of the reaction was analyzed by HPLC using the gradient 0-60% B over 30 min and the same gradient was used to isolate the di-Ub(K63)-Cys-SUMO(2-93, A23C*, A74C*) **22** in 40 % yield (5.1 mg).

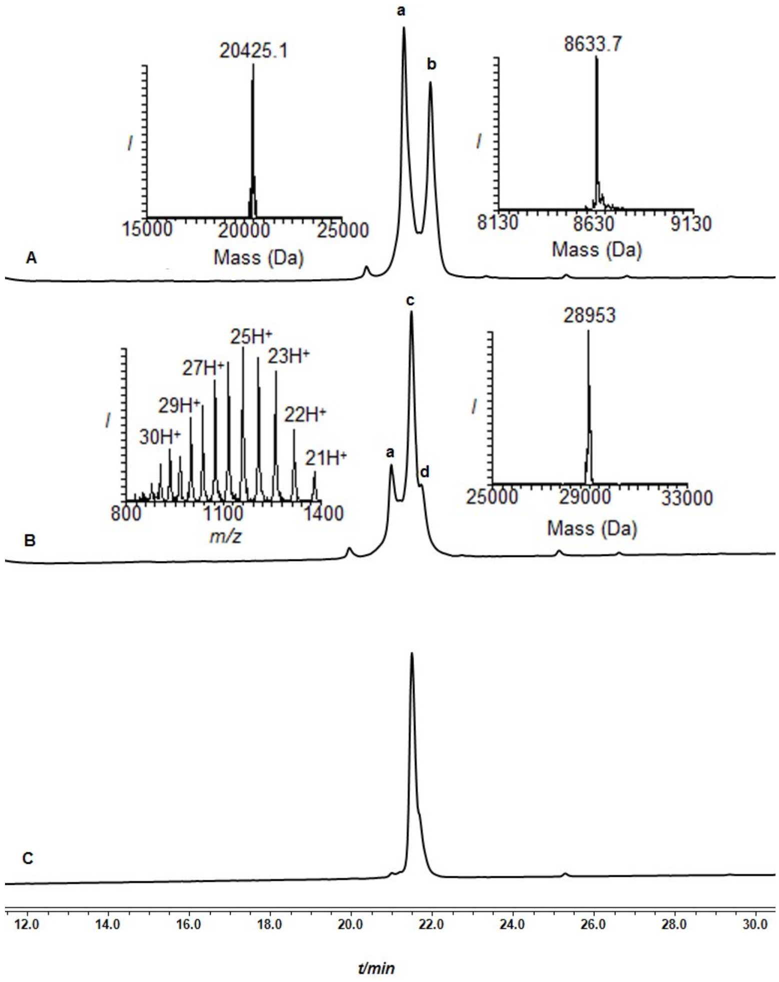

Figure S27: Synthesis of di-Ub(K63*)-Cys-SUMO-(2-93, A23C*, A74C*). A) Analytical HPLC and mass traces of the ligation between Ub(K63*)-Cys-SUMO(2-45, A23C*, A74C*) and Ub-MPA, peak a corresponds to Ub(K63*)-Cys-SUMO(2-45, A23C*, A74C*) with the observed mass of 20425.1 ± 2.8 Da, calcd 20428.2 Da, peak b corresponds to Ub-MPA with the observed mass of 8633.7 ± 0.2 Da, calcd 8633.9 Da, at time zero. B) Ligation after 7 h: peak c corresponds to desired ligation product di-Ub(K63*)-Cys-SUMO-(2-93 A23C*, A74C*) with the observed mass of 28,953 ± 3.2 Da, calcd 28,957.0 Da, peak d corresponds to Ub-COOH. C) Analytical HPLC of purified di-Ub(K63)-Cys-SUMO-(2-93 A23C*, A74C*).

One pot Phacm removal and desulfurization for the synthesis of di-Ub(K63)-Ala-SUMO-(2-93)

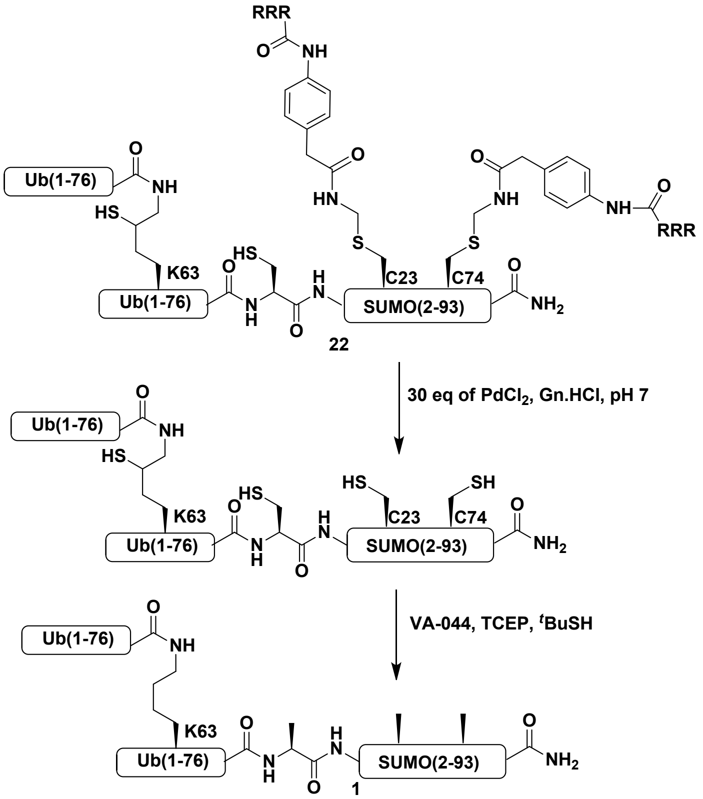

di-Ub(K63)-Cys-SUMO(2-93, A23C*, A74C*) **22** (5.1 mg, 1.76×10^{-4} mmol) was dissolved in 6 M Gn•HCl, 200 mM phosphate buffer (170μL, 1mM) containing 40 mM of $PdCl_2$ and the reaction mixture was kept at 37 °C for 2 h. Progress of the reaction was monitored by taking aliquot from the reaction mixture and treated with a small amount of DTT, followed by centrifugation and injection of the supernatant into analytical HPLC (C4 column) using a gradient of 0-60% B over 30 min. After completion of the reaction, 150 equiv of DTT was added and the resulting precipitate was separated by centrifugation. The precipitate was washed with Gn•HCl buffer twice and the combined supernatant solution was dialyzed in a Slide-A-Lyzer 3.5K dialysis cassette (Thermo scientific, 0.1- 0.5 mL) in 6 M Gn•HCl, 200 mM phosphate buffer (500 mL) for overnight. Subsequently, the reaction mixture was desulfurized by treating with 28.5 mg TCEP, 10 mg (100 equiv) VA-044 and 40 μL *t*BuSH for 10 h. Progress of the reaction

was monitored by analytical HPLC using C4 column with a gradient of 0-60% buffer B over 30 min. For semi-preparative HPLC, the same gradient was used to isolate the di-Ub(K63)-Ala-SUMO-(2-93) in ~39% isolated yield (2 mg).

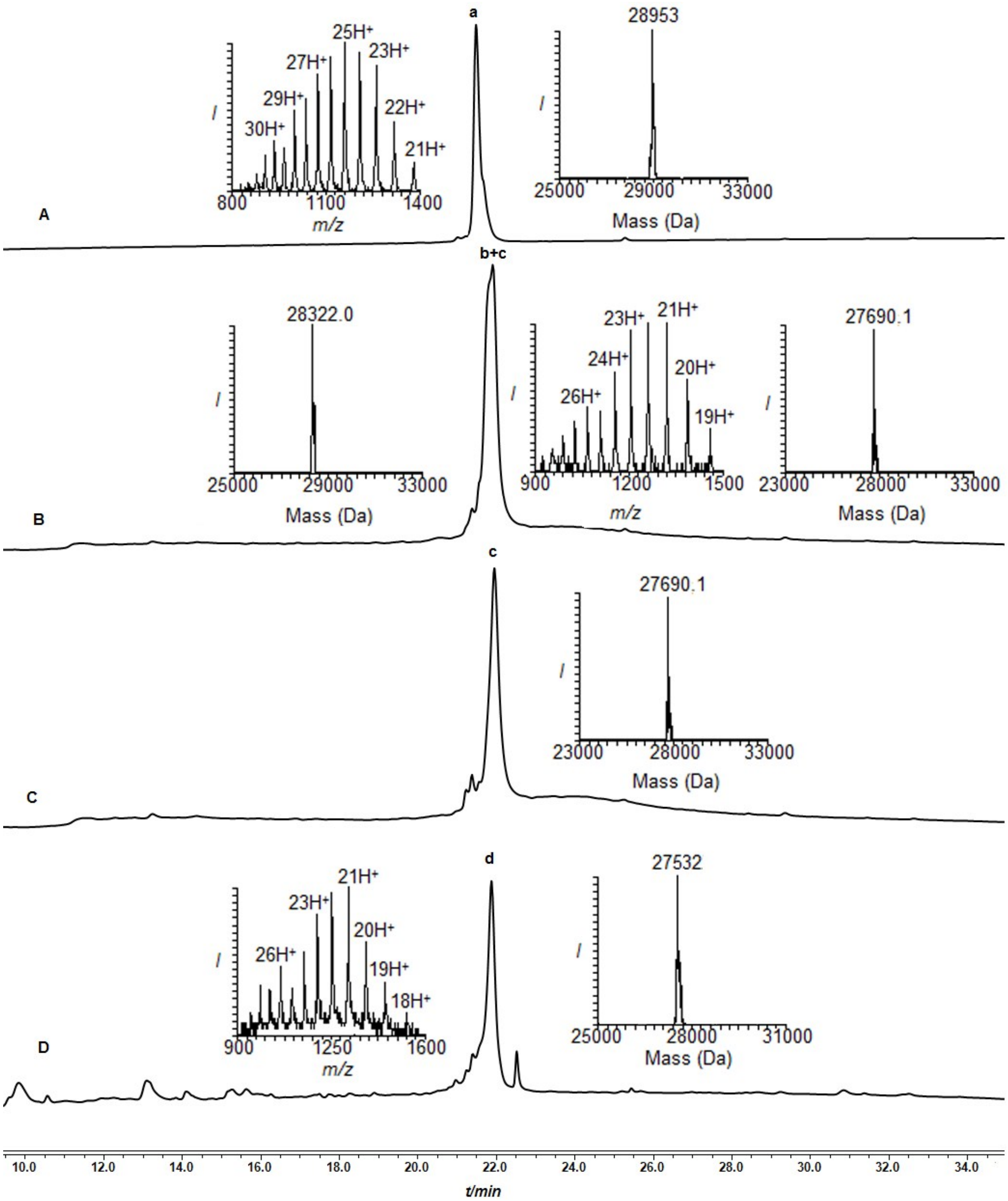

Figure S28: Removal of solubilizing tag from di-Ub(K63)-Cys-SUMO-(2-93 A23C*, A74C*) using $PdCl_2$. A) Analytical HPLC and mass trace of the of di-Ub(K63)-Cys-

SUMO-(2-93 A23C*, A74C*) and $PdCl_2$, peak a corresponds to di-Ub(K63)-Cys-SUMO-(2-93 A23C*, A74C*) with the observed mass of 28,953 ± 3.2 Da, calcd 28,957.0 Da, at time zero. B) Removal after 1 h: peak b+c correspond to removal of one and two tags with the observed masses of 28322.0 Da and 27,690.1 Da respectively. C) Removal after 2 h: peak c corresponds to a complete removal of two tags with the observed mass of 27,690.1 ± 3.5 Da, calcd 27695.5 Da. D) After 8 h of desulfurization of the dialyzed reaction mixture: peak d corresponds to a complete removal of two tags with the observed mass of 27,532.0 ± 1.1 Da, calcd 27535.2 Da.

HPLC overlay of Ub(K63)-SUMO-2 purification in the 3 different strategies:

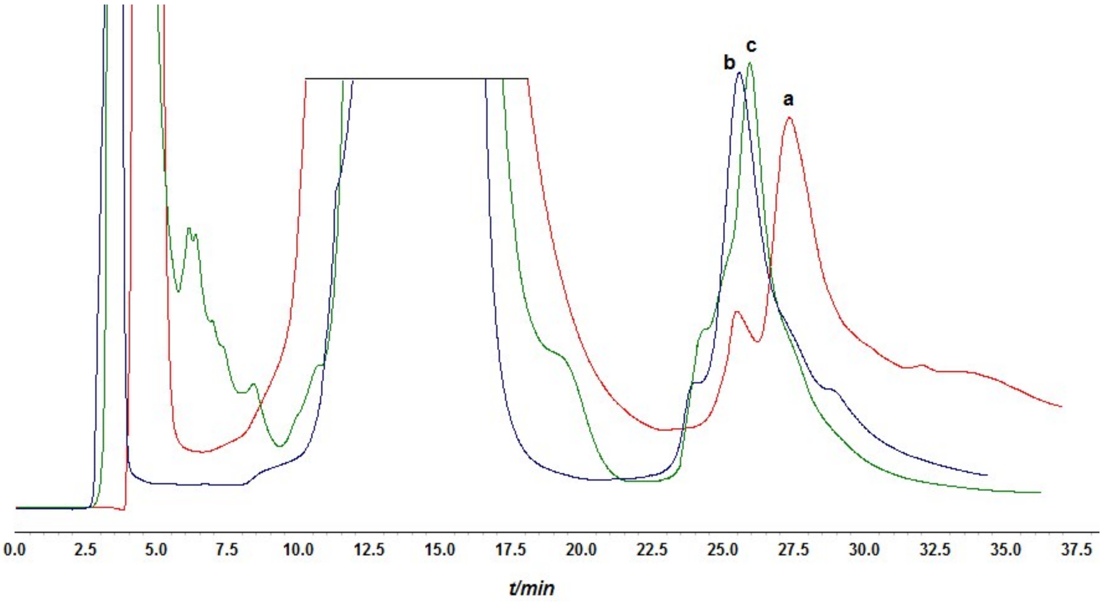

Figure S29: a) Peak corresponding to Ub(K63*)-Cys-SUMO(2-93), b) Peak corresponding to Ub(K63*)-Cys-SUMO(2-93)-Dbz-$(Arg)_6$, c) Peak corresponds to Ub(K63*)-Cys- SUMO-(2-93, A23C* & A74C*).

Circular dichroism spectra of SUMO, SUMO-Ub, SUMO-diUb and K63-diUb:

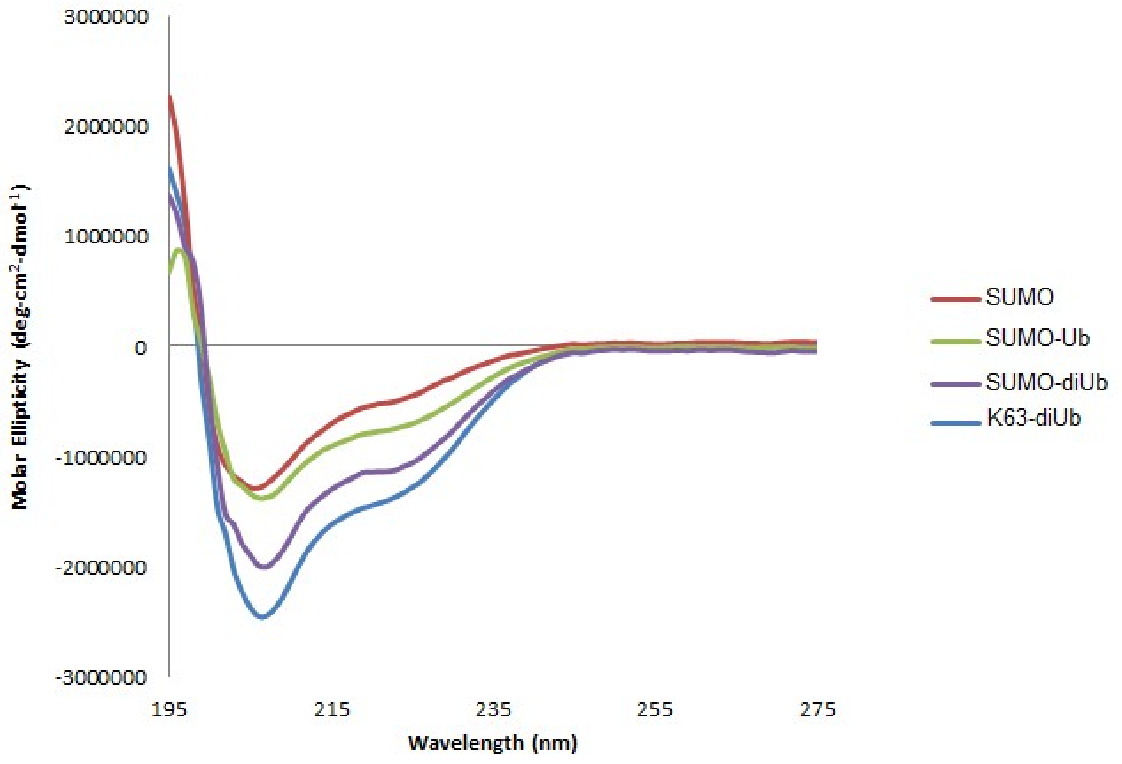

Circular dichroism spectra and mass analysis of di-Ub(K63)-Lys11-SUMO-2:

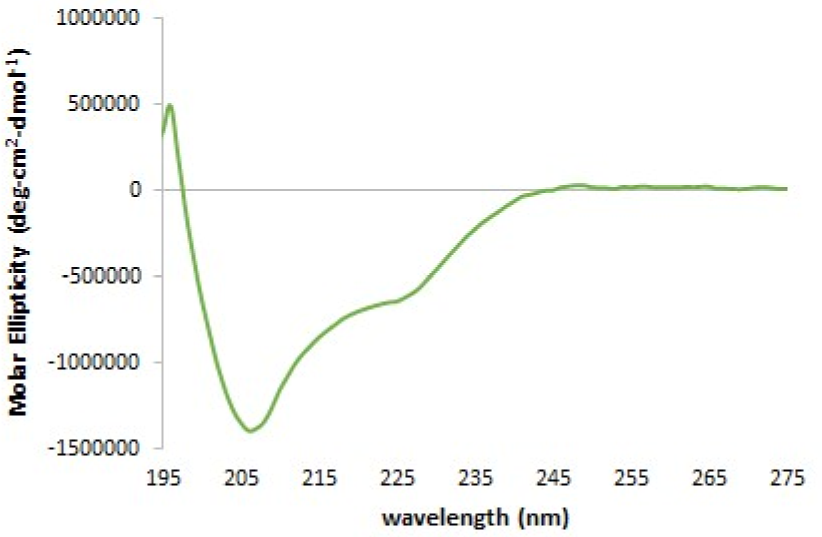

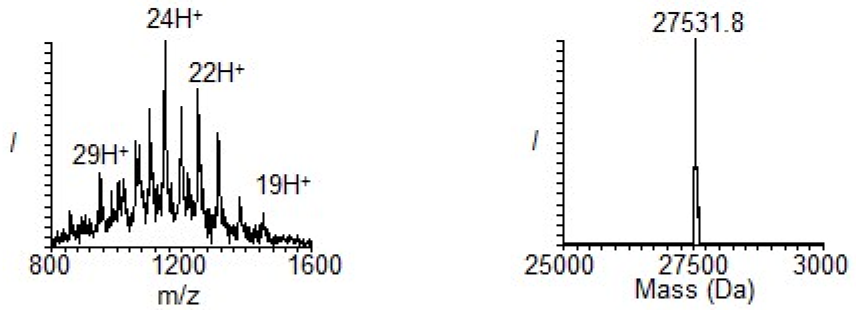

Circular dichroism spectra and mass analysis of di-Ub(K63)-Lys33-SUMO-2:

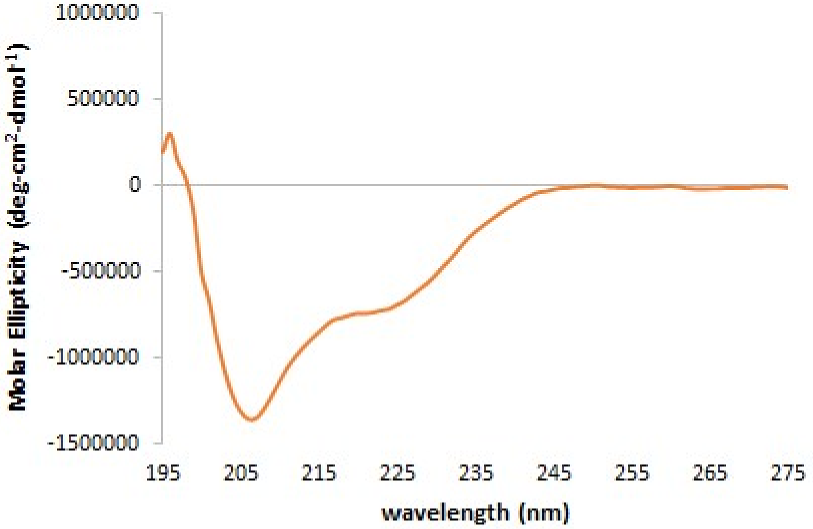

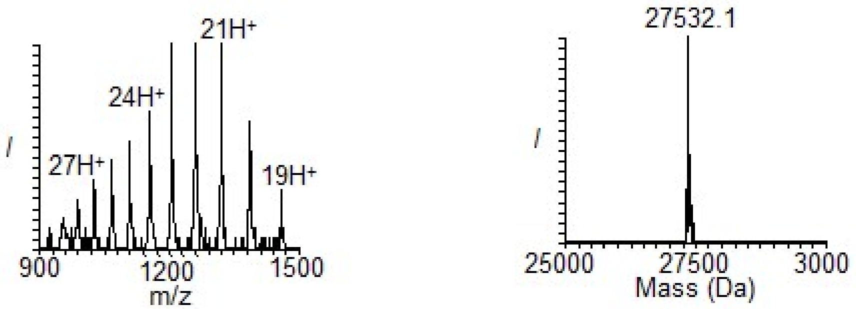

Circular dichroism spectra and mass analysis of di-Ub(K63)-Lys42-SUMO-2:

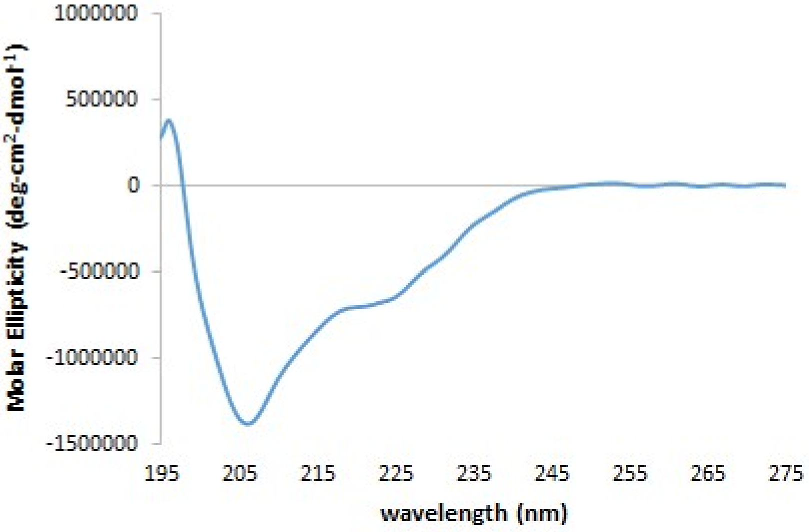

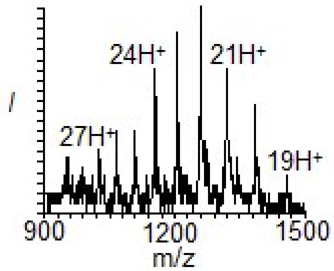

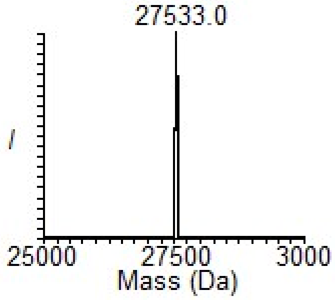

References

1. J. B. Blanco-Canosa, B. Nardone, F. Albericio and P. E. Dawson, *J. Am. Chem. Soc.*, 2015, **137**, 7197-7209.
2. L. A. Erlich, K. S. Kumar, M. Haj-Yahya, P. E. Dawson and A. Brik, *Org. Biomol. Chem.*, 2010, **8**, 2392-2396.
3. S. K. Maity, G. Mann, M. Jbara, S. Laps, G. Kamnesky and A. Brik, *Org. Lett.*, 2016, **18**, 3026-3029.
